# The impact of telemonitoring on correct drug use, complications and quality of life among patients with multiple myeloma (ITUMM): A study protocol for an open-label, parallel-group, randomized controlled trial

**DOI:** 10.1371/journal.pone.0307177

**Published:** 2024-08-26

**Authors:** Job F. H. Eijsink, Paul A. F. Geerts, Karin Kamminga, Mireille A. Edens, Cornelis Boersma, Maarten J. Postma, Jan Gerard Maring, Peter G. J. ter Horst

**Affiliations:** 1 Department of Clinical Pharmacy, Isala, Zwolle, The Netherlands; 2 Department of Health Sciences, University of Groningen, University Medical Center Groningen, Groningen, The Netherlands; 3 Department of Internal Medicine, Isala, Zwolle, The Netherlands; 4 Department of Innovation and Science, Isala, Zwolle, The Netherlands; 5 Department of Management Sciences, Open University, Heerlen, The Netherlands; 6 Department of Economics, Econometrics & Finance, Faculty of Economics, University of Groningen, Groningen, The Netherlands; Royal College of Surgeons in Ireland, IRELAND

## Abstract

**Introduction:**

Multiple myeloma (MM) is the second most common hematologic malignancy. MM is associated with significant morbidity due to its end-organ destruction and is a disease of the older population. Although survival rates for MM have improved over the last decade, due to an increase in treatment options, the disease remains incurable. Expensive (oral) agents are widely used in MM patients; however, tools for supporting patients in complex treatment regimens are scarce. To investigate if a tool will support MM patients and healthcare professionals, the MM e-coach was developed and tested. The aim of this study is to study the impact of telemonitoring on adherence, complications and quality of life in patients with MM (ITUMM study).

**Methods:**

A two-arm open-label parallel-group randomized controlled trial will be conducted between March 2021 and June 2024 to compare the telemonitoring (MM e-coach) with standard MM care. This study aimed to recruit 150 patients with recently diagnosed multiple myeloma (RDMM), starting first or second line of treatment. Blinded primary outcome is adherence by pill count after start of treatment at 1–3 months. Secondary outcomes are patient reported outcomes: GFI, EQ-5D-5L, EORTC-QLQ-C30, SDM-Q-9, MARS-5, single item questions, PREMs, adverse events, OS and PFS. Patient reported outcomes were developed and integrated in the e-coach MM to regularly measure digitized outcomes of MM patients from time of RDMM until 12 months post-diagnosis. Online measurements will be performed at baseline (0), 3, 6, 9 and 12 months.

**Ethics and dissemination:**

Ethics approval has been granted by the Ethics Committee of the Isala klinieken in The Netherlands (No. 201111) at 25 February 2021. Study results will be disseminated to the relevant healthcare communities by publication in peer-reviewed journals, and at scientific and clinical conferences.

**Study registration number:**

ClinicalTrials.gov number: NCT05964270 and ABR number: NL75771.075.20.

## Introduction

Multiple myeloma (MM) is one of the most prevalent hematological malignancies globally, and is associated with substantial morbidity and mortality [1]. It originates from the malignant proliferation of plasma cells in the hematopoietic tissue of the bone marrow and potentially leads to tissue and organ damage [2]. Patients with MM usually have hypercalcaemia, renal failure, anaemia, or osteolytic bone lesions [2].

In Europe, the age-standardized incidence of MM is reportedly approximately 5 cases per 100,000. The median age at diagnosis is approximately 65–70 years, with 37% of patients being <65 years of age [1]. Incident cases from 1990 to 2016 increased by 126% globally [3]. In the Netherlands the incidence of MM is about 1500 new diagnoses on 18 million citizens each year [4]. Despite the significant improvement in patients’ survival over the last two decades, only 10–15% of patients achieve or exceed expected survival compared with the matched general population [5].

Although survival rates for MM have improved over the last years, due to improved treatment options, the disease remains incurable [1]. Expensive agents are widely used in MM treatment due to their proven efficacy in different MM treatment lines [6, 7]. Treatment regimens are complex and new MM (combination) regimens are expected to be added in the coming years [8–10]. A recently diagnosed MM (RDMM) patient often uses 10–15 oral drugs a day, including necessary concomitant drugs to prevent or treat infection, thrombosis, nausea, and pain. Such treatment schedules may be difficult to understand for patients [11–13]. The number of elderly patients with MM will probably increase as result of the improved survival rates that are associated with novel treatments combined with an increasing life expectancy of the general population [14, 15]. Age-related changes in physiology combined with comorbid conditions, disability or frailty have important implications for the treatment of MM patients [15].

The concept of value-based healthcare (VBHC) is defined as a shift from volume-driven healthcare to patient value [16]. Outcomes representative for the individual at micro level are crucial, but also on macro level, to identify the patient value for a population [17]. Furthermore, patient involvement is important in developing a VBHC care pathway, with focus on the needs of patients and the ensuing beneficial results for each patient [18]. For this study we have defined VBHC outcomes, by using an approach that is aligned with patient-centered MM care, including the use of well-defined outcome measures. Patient-reported outcomes expressed in patient-reported outcome measures (PROMs) and patient-reported experiences measures (PREMs) are leading in the concept [19, 20]. A dynamic way of VBHC development and implementation of patient-reported outcomes in hospital based interventions are crucial and leads to an equal and proactive role in a multidisciplinary team of healthcare professionals [18, 21].

There is an ongoing trend towards shifting care to an outpatient setting following intensive cancer therapy [22]. Telemonitoring is a viable alternative tool for care and has been encouraged by the rising costs of care, scarcity in healthcare professionals, rapid advances in communication and diagnostic technology, and the wider availability of low‐cost, patient‐friendly telecare equipment [23–26]. Patient involvement and shared decision making (SDM) seemed to be unknown to most patients and healthcare professionals in the development and implementation of care interventions [27, 28]. Tools such as e-health applications, to support implementation of SDM practices could be very helpful.

**Fig 1:**
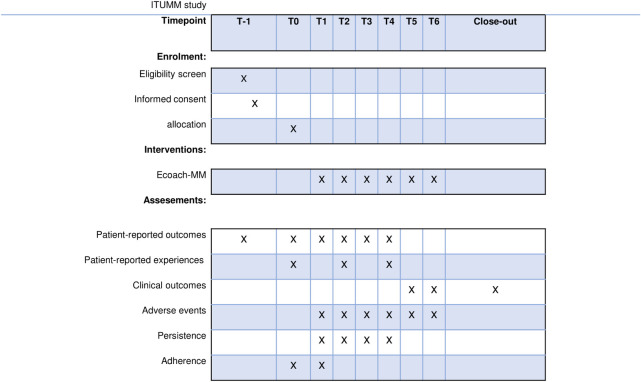
SPIRIT schedule of enrolment, interventions and assessments of the ITUMM study.

**Fig 2:**
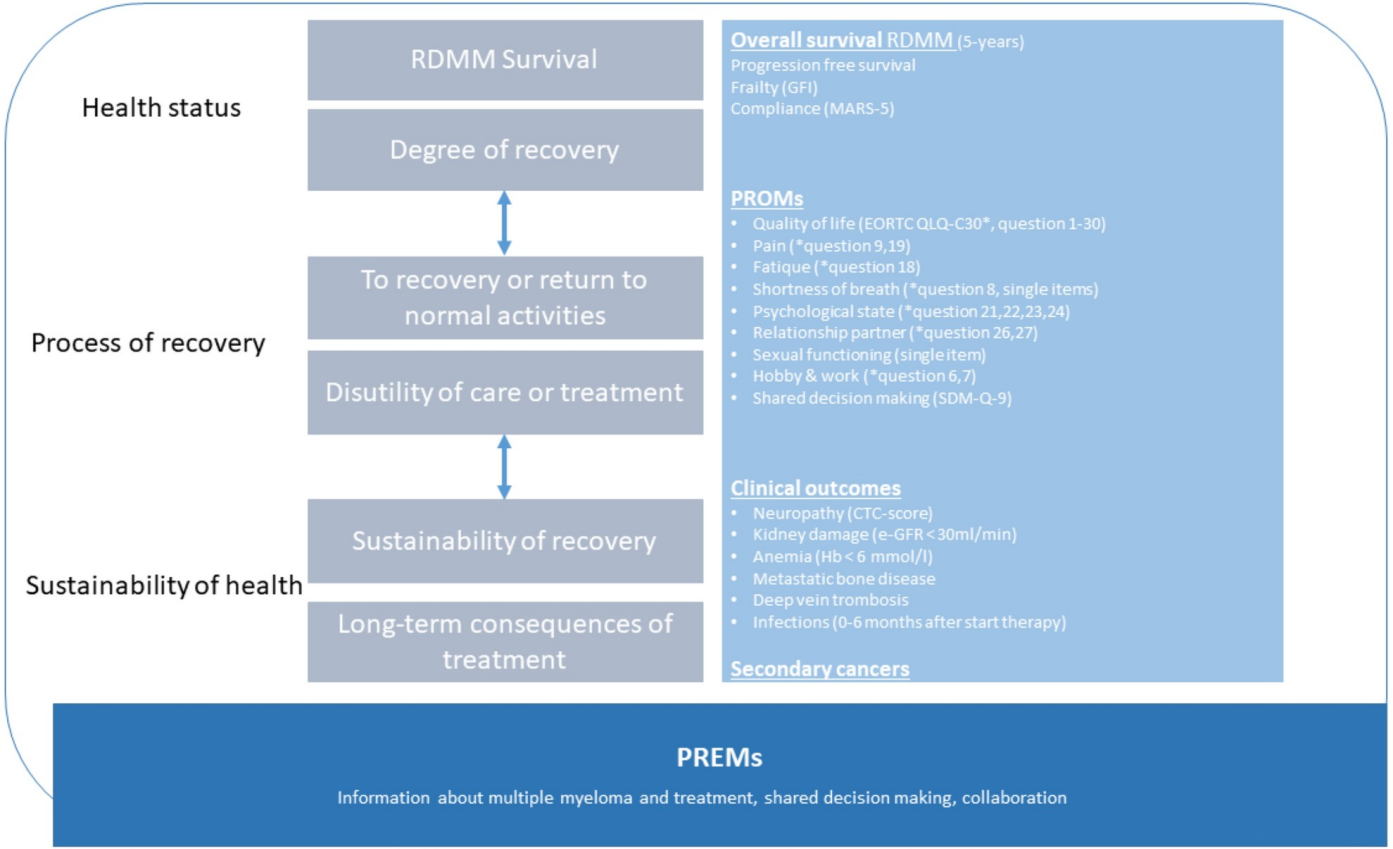
VBHC outcome set in the ITUMM study. GFI: groninger frailty index is a questionnaire in which the dimensions physical and psychosocial frailty are measured. The questions relate to the situation of the past month or the situation before the patient became RDMM.; EORTC-QLQ-C30: The EORTC QLQ-C30 is a 30 item questionnaire and is one of the most widely used health-related quality of life (HRQOL) questionnaires in cancer research; MARS-5: The Medication Adherence Report Scale is a 5 item measurement, aimed to develop a questionnaire measure of patients ’adherence to medication’; SDM-Q-9: is a 9-item questionnaire and measures the extent to which patients are involved in the process of decision-making from the perspective of the patient PROMs: patient reported outcome measurements; PREMs: patient reported experiences measurements.

In this study (Fig 1) we developed a multimodal patient-centered MM e-coach between September 2019 and June 2020. The content of the intervention consist of eight modules and was tested in a pilot study between June 2020 and August 2020 (F-ITUMM) [21]. The e-coach is digitally managed, following three phases of the tiers of VBHC as presented in Fig 2. The tiers are classified in health status, process of recovery and sustainability of health [29]. Furthermore, all medication information plus current dose and frequency per unit time are integrated in the e-coach. Reminders are sent if a session or a medication unit time is exceeded. Feasibility was tested for patients as well as healthcare professionals. The F-ITUMM trial concluded that the MM e-coach has the potential to support both RDMM patients and healthcare professionals during MM treatment, and is a potential application to improve adherence [21].

The World Health Organization (WHO) identified incorrect intake or non-adherence to therapy as one of the main causes of morbidity, mortality and the increasing health costs in the world [30]. In patients with hematological malignancies, studies have reported adherence results rates in patients with chronic myeloid leukemia of 20–53%, in patients with acute lymphoid leukemia non-adherence rates of 6–35% and patients with MM of 30–67% [31–33] Factors reported to be associated with non-adherence included increasing age, higher comorbidity, polypharmacy, and a lack of social support [34]. Adherence to long-term therapy for chronic illnesses in developed countries is close to 50% [35, 36]. With regard to oral anticancer agents, adherence rates range from 40–100% and also puts financial strain on the healthcare system [37]. Depending on disease and healthcare system, an estimated 30–50% of patients do not use intended or intended, their medication as prescribed [31, 38].

The aim of this study is to evaluate the adherence of oral MM agents using a MM e-coach compared to standard MM care. The blinded primary endpoint is drug adherence at 3 months after start of oral MM treatment. The secondary endpoints are: adherence after 12 months, medication persistence (PDC), groninger frailty index (GFI), Quality of life (EQ-5D-5L, EORTC-QLQ-C30), shared decision making (SDM-Q-9), adherence (MARS-5), single item questions and PREMs will be analyzed. Differences in clinical outcomes: progression-free survival, overall survival, adverse events, hospital admissions (hospitalization linked to oncology treatment) and hospital costs are additional outcomes.

### Methods

#### Study design

**Fig 3:**
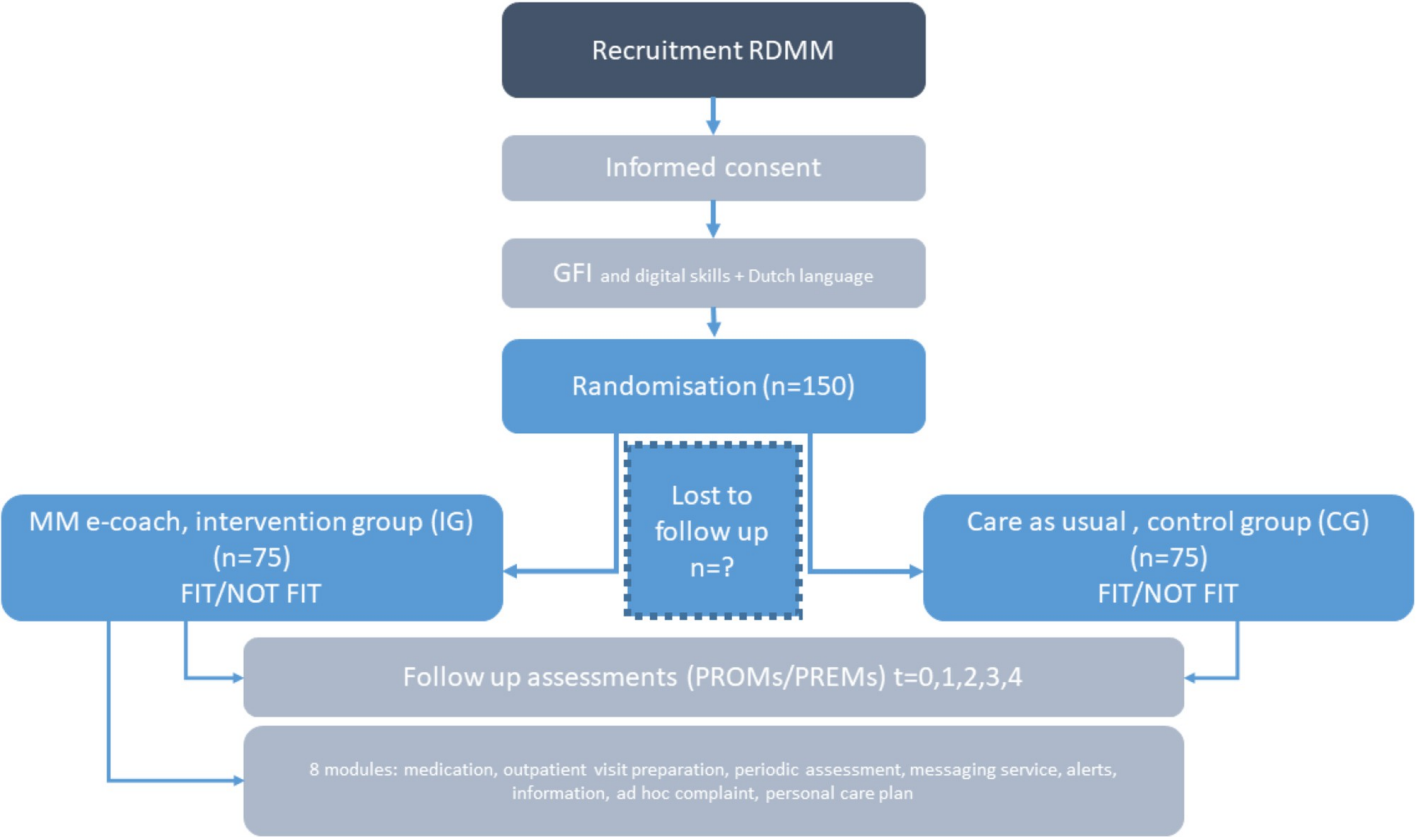
Flow diagram of the study procedure. RDMM: recently diagnosed multiple myeloma patient, GFI: groninger frailty index.

A two-arm open-label parallel-group randomized controlled trial (RCT) will be conducted between March 2021 and June 2024, at a supra regional cancer center (referred to as ‘het oncologisch centrum Isala’), which provides hematology services to a population with 0.5 million adherence and a population of 1 million referral, in the Northern-East region of The Netherlands. This protocol study is reported according to the SPIRIT (Standard Protocol Items: Recommendations for Interventional Trials) 2013 Statement [39]. This study has been approved by the Ethics Committee of the Isala klinieken in The Netherlands (No. 201111). ITUMM–study registration ClinicalTrials.gov number: NCT05964270 and ABR number: NL75771.075.20. Written informed consent will be obtained from all subjects. This study is registered in the general assessment and registration form number: NL75771.075.20 trial register in the Netherlands and Clinical.Trials.gov number: NCT05964270. The final report of the RCT will be conducted and reported in accordance with the Consolidated Standards of Reporting Trials (CONSORT) Statement for pilot RCTs as well as the guidelines for executing and reporting internet intervention research [40]. A flow diagram of the study is presented in Fig 3.

### Eligibility criteria

In order to be eligible to participate in this study, a participant (patient) must meet all of the following criteria: signed informed consent, >18 years; RDMM patients with first-line or second-line treatment and able to complete patient-reported outcome measures and experiences, have minimal digital skills to check if they are able to interact with an e-coach, and can read and understand Dutch. Participants who meet any of the following criteria will be excluded from participation in this study: psychiatric illness requiring secondary-care intervention; too ill to engage with the intervention in the opinion of the clinical care team; and no perspective of >12 months survival.

### Participants

A participant with RDMM is a new diagnosed patient with first relapse or refractory to first line of therapy with start of the treatment within 1 month. A RDMM participant could be ‘FIT’ or ‘NOT FIT’. A ‘FIT-patient’ is eligible for hematopoietic stem-cell transplantation, which affects the scope of treatment options and survival rate compared to a ‘NOT FIT patient’. In addition, a frailty index score has been included in the RCT (Figs 1 and 2), which is planned in a multidisciplinary meeting shortly after a diagnosis [41, 42]. In the RCT, the age limit of 70 years is used with a high frailty index as ‘NOT FIT’, all patients who are younger and score well on the frailty index are therefore eligible for a transplantation and a wider scope of treatment options. Furthermore, when a patient is on the borderline of the criteria, both the hematologist and the multidisciplinary meeting (in this case a team of physicians, nurse practitioners and pharmacist), should decide whether this patient can be marked as a ‘FIT-patient’ and will consequently be treated in the ‘FIT-group’.

Every potential participant of the study is eligible for first-line or second-line treatment. Second-line treatment is treatment for a disease or condition after the initial treatment (first-line treatment) has failed, or has side effects that cannot be tolerated. These patients will then be treated for MM according to local standard of care, including supportive care (infection prophylaxis, thrombosis prophylaxis).

### Recruitment

In this study, following diagnosis, eligible patients will receive a brief overview of the study protocol and a patient information folder. They will then be introduced to the principal investigator for further discussion. Patients can indicate their interest by returning the reply slip. Patients will be contacted by phone within 1 week after diagnosis to discuss whether they want to participate. After signing (1–2 weeks after receiving patient information folder) off informed consent and handing the form to the PI, patients will be randomized and included in the study. Recruitment started in March 2021 and is still ongoing until the target sample size will be reached or after 20 months of inclusion, even if the target sample size could not be reached.

### Study procedure

The preparation phase consists of training the study team, informing the treatment team (pharmacist, nurses and hematologists) and selection of potential study participants. The recruitment of participants including providing the study participant with an informed-consent letter informing him/her of current practices and how to use the e-coach. Participants will be informed that their participation is voluntary, after which they will be required to sign a statement of informed consent that meets the requirements of 21 CFR 50, local regulations, ICH guidelines, Health Insurance Portability and Accountability Act requirements (where applicable), and the IRB/IEC or study site. The participant must personally sign and date the IRB/IEC informed consent before the study-specific procedures can be started. After signing, the randomised allocation (Fig 2) of participants will be conducted. In the execution phase, participants of the intervention group (IG) will get a login for the e-coach MM, and access to eight modules: medication, outpatient visit preparation, periodic assessment, messaging service, alerts, information, ad hoc complaint, personal care plan [21]. IG participants information is collected on a web platform that automatically invites enrolled patients. The e-coach MM is available 24/7. The link to the preoperative questionnaire is sent by SMS and/or email within 24 hours after enrolment. Participants in the CG (control group) will only get a login for a ‘dummy version’ without the modules, besides the periodic assessments (questionnaires), at the same time as the IG. Healthcare professionals can access the web platform to monitor the surveys by checking their enrolment and response rates from the electronic patient record in the hospital. Participants can leave the study at any time for any reason if they wish to do so, and without any consequences. The last phase is evaluation and analysis during and after the study period, from June to December 2024.

### From diagnosis to 3 months for IG

- Preparation of treatment plan and personal care plan with input from the Groningen Frailty Index (GFI) in the multidisciplinary team in the IG and CG.
- Outpatient visit preparation (patient).
- Preparation of the outpatient visit preparation in the e-coach and personal care plan (hematologist).
- Preparation of the ad hoc complaints and medication in the e-coach (nurse).
- (Actively) follow-up of signals (red flags), messages and monitoring (nurse and hematologist).
- Pill count (clinical pharmacy).
- All periodic assessments, single item questions and clinical outcomes are measured in the IG and CG.

### At 3 months (t = 1) for IG

- Outpatient visit preparation (patient).
- Preparation of the outpatient visit preparation in the e-coach and personal care plan (hematologist).
- Preparation of the ad hoc complaints and medication in the e-coach (nurse).
- (Actively) follow-up of signals (red flags), messages and monitoring (nurse and hematologist).
- Pill count (clinical pharmacy).
- All periodic assessments, single item questions and clinical outcomes are measured in the IG and CG (Table 3).

3–6 months (t = 2), same as at 3 months, with exclusion of the pill count.

6–9 months (t = 3), same as at 3 months, with exclusion of the pill count.

9–12 months (t = 4), same as at 3 months, with inclusion of the pill count.

Follow-up of PFS at 12 and 36 months, and OS at 60 months.

### Randomisation

Randomised allocation of participant to two groups will be conducted by an independent research assistant, who is not involved in other processes of the study, with blinded envelopes. Randomisation scheme is developed by the PI in Excel. Based on numbers 1–75 and 76–150, envelopes are created with ‘FIT’ and ‘NON-FIT’ added, these envelopes are placed near the hematology department. The nurse can, if the patient has given consent, pick up the envelope labelled with ‘FIT’ or ‘NON-FIT’ at the next appointment, assigning the patient to either intervention or control group. Randomization envelopes were created by the PI, which was not involved in the actual randomization process. Available codes for block randomization were used. The nurses randomizing patients can only see the stratum, i.e. “FIT” and “NON-FIT”, and the sequence number of randomization envelopes. Envelopes are closed and not transparent. Therefore concealment of allocation is guaranteed and randomised allocation was stratified based on patient fitness ‘FIT-patient’ or ‘NOT FIT- patient’ with an allocation ratio of 1:1 [43]. Participants, health-care professionals, and staff who assess outcome measures will not be masked to treatment allocation.

### Blinded primary outcome

Primary outcome of this study is the adherence with oral MM treatment, defined as “the extent to which a person’s behavior in taking medication or making lifestyle changes agrees with recommendations from a healthcare provider” [30]. Medication adherence refers to the act of confirming to the recommendations made by the provider with respect to timing, dosage and frequency of medication taking [44, 45]. Adherence is measured in this study over a time period of 3 months and reported as a percentage (Table 1) by pill counts. The pill count methodology avoids the effect of improved behavior, because patients would generally not be aware that their refill rates would eventually be reviewed at the pharmacy [36, 46, 47]. Pill count is conducted by the trial department of the clinical pharmacy at the hospital, by four different qualified trial assistants. Pill count is blinded for both groups.

**Table 1:** Primary outcome calculation.

Adherence per patient
Month 1: 100%- [missed doses / divided total count of pills] *100 = adherence in month 1 (30 days)
Month 2: 100%- [missed doses / divided total count of pills] *100 = adherence in month 2 (60 days)
Month 3: 100%- [missed doses / divided total count of pills] *100 = adherence in month 3 (90 days)
**Overall adherence (%) = (months 1 + 2 + 3) / divided 3))**
Adherence difference
**Month 1 = +/- 30 days (intervention group) vs. month 3 = +/- 90 days (intervention group)**
**= difference intervention group**
**Month 1 = +/- 30 days (control group) vs. month 3 = +/- 90 days (control group)**
**= difference control group**
**Difference intervention group–difference control group = adherence difference in 3 months**

### Secondary outcomes

#### Persistence

The Proportion of Days (PDC) calculates the ratio [number of days covered by medication] / [total number of days in the designated period]; and the PDC can be calculated for multiple simultaneous medications within a specific period. For calculations of the PDC used in multi-therapies, see Table 2. Adherence is defined as percentage of dosages taken as prescribed, and persistence over the period the medication was taken [45, 48].

**Table 2:** PDC calculation.

**Proportion of Days Covered (PDC) per patient**
Month 1: [n days in period covered / n days in the month] * 100%
Month 2: [n days in period covered / n days in the month] * 100%
Month 3: [n days in period covered / n days in the month] * 100%
**Months 4–12**
**Total mean PDC: Month [1+2+3+4+5+6+7+8+9+10+11+12] / [divided 12 months]**

#### Medication Adherence Rating Scale (MARS-5)

An important secondary outcome (Table 3) is the patient-reported MARS-5 score [49]. Adherence percentage as reported by the patients themselves could be for example higher compared to adherence by pill count. The MARS-5 consists of 5 items describing non-adherent behaviors (“I forgot to take the medicine / I altered the dose of medicine / I stopped taking the medicine for a while / I decided to miss out a dose / I took less than instructed”). Patients are asked to evaluate how often they adopt each behavior on a 5 point scale, ranging from ‘always’ to ‘never’ (1–5 points). The total score on the scale ranges from 5 (lowest adherence) to 25 points (maximal adherence) [49, 50]. At one MARS-5 sum score of 21 or a score of 4 on each individual item, the patient is considered adherence to therapy.

**Table 3:** Patient-reported outcomes.

Patient reported outcomes								
**Questionnaires**	T-1	T0	T1	T2	T3	T4	T5	T6
GFI	✔							
EQ-5D-5L		✔				✔		
EORTC QLQ-C30		✔	✔	✔	✔	✔		
SDM-Q-9		✔				✔		
MARS-5		✔	✔	✔	✔	✔		
PREMs		✔		✔		✔		
**Single item questions**								
Pain		✔	✔	✔	✔	✔		
Neuropathy			✔	✔	✔	✔		
Shortness of breath		✔	✔	✔	✔	✔		
Sexual functioning				✔		✔		
**Clinical outcomes**								
Progression-free survival							✔	
Overall survival								✔

GFI: groninger frailty index is a questionnaire in which the dimensions physical and psychosocial frailty are measured. The questions relate to the situation of the past month or the situation before the patient became RDMM.; EORTC-QLQ-C30: The EORTC QLQ-C30 is a 30 item questionnaire health-related quality of life questionnaires in cancer research; MARS-5: The Medication Adherence Report Scale is a 5-item measurement, aimed to develop a questionnaire measure of patients ’adherence to medication’; SDM-Q-9: is a 9-item questionnaire and measures the extent to which patients are involved in the process of decision-making from the perspective of the patient PROMs: patient-reported outcome measurements; PREMs: patient-reported experiences measurements.

#### Quality of Life (EQ-5D-5L and EORTC-QLQC30)

In this study we use two patient-reported Quality of Life scores. Quality of life (QOL) is important in patients with advanced cancer. The European Organisation for Research and Treatment of Cancer (EORTC) QLQ-C30 is a general QOL tool used in cancer patients [51].

The EORTC QLQ-C30 is a more general QOL instrument designed for patients with cancer. It contains five functional, three symptom scales, a global health status/QOL scale, and a number of single item questions assessing additional symptoms commonly reported by cancer patients and perceived financial impact of the disease. In total there are 30 items on the questionnaire. Each item in the EORTC QLQ-C30 is rated from 1 (not at all) to 4 (very much) in severity, except for the overall QOL scale, which is rated from 1 (very poor) to 7 (excellent).

The EQ5D-5L comprises five dimensions: mobility, self-care, usual activities, pain/discomfort and anxiety/depression [52]. Each dimension has 5 levels and is asked at t = 0 and 12 months. The EQ5D-5L results will be used for a cost-effectiveness analysis, after the ITUMM-study.

#### Shared decision making (SDM-Q-9)

The SDM-Q-9 is a self-reported questionnaire, in this study we used the Dutch version [53]. It contains two open-ended questions “For what reason did you visit your doctor?” and “What decision has been made (e.g. which treatment)? This is followed by nine closed questions for which a maximum of 6 points can be scored per question. The total score (maximum of 45 points) represents the level of perceived SDM. In this study the SDM-Q-9 is asked at t = 0, 6 and 12 months form the e-coach. We will use the SDM-Q-9 as an explorative outcome in this study.

#### MM-specific patient reported outcomes and experiences

Development of specific questions was determined during the development of the new MM care pathway with involvement from patients and healthcare professionals. PROM questionnaires (Table 3) are patient-reported outcomes and are asked at different times over the year, dependent on the possible side effects of MM treatment and psychological/social aspects (Fig 1 and Table 3). PREM questionnaires are patient-reported experiences and are asked at t = 0, 6 and 12 months from the e-coach. The questionnaire included 4 items concerned with experience, from the general patient satisfaction survey in the hospital. PREMs are: shared decision-making, collaboration between hospital practitioners, information about the use of medication and information about the hospital (Fig 2 and Table 3).

#### MM-specific clinical outcomes

Further, we will study explorative parameters: Progression-Free Survival (36 months), mortality (after 60 months), number of adverse events (grades III and IV), and number of hospital admissions (hospitalization linked to oncology treatment). Anemia (hemoglobin), kidney damage (eGFR), metastatic bone disease and deep-vein thrombosis. The measures start at t = 0 (diagnosis of MM) until 60 months.

#### Sample size/power calculation

The sample size of this study is based on the primary outcome regard to the pill count at t = 3 months after randomization. Pill count is a continuous measure expressed in percentages and has a normal distribution. Due to the unpredictability of the disease and the vulnerability of this group of patients, we used a conservative approach to sample-size calculation, based on an unpaired *t*-test and was performed using SPSS SamplePower3.0. Calculation for pill count [14, 54] Vulnerability and frailty are factors that could have an impact on our inclusion rate; therefore, we calculated a conservative sample size. At t = 3 months, we hypothesized a minimal clinically relevant difference of 10%, standard deviation 20% [55], power 80%, and 2-tailed alpha 5%. This calculation results in 64 patients in each arm. Taking into account an attrition rate of 15%, yields 75 patients to be included in each arm. Therefore, in total 150 patients will be included in this study [56–59]. Duration of the trial is 20 months of inclusion plus 12 months’ time per participant in the study (each month 8 participants).

#### Data collection and analyses

The collection, storage and processing of personal data in this study are carried out in accordance with the applicable data protection regulations of the federal states in connection with the European General Data Protection Regulation (GDPR) during the study, all data recorded electronically or in the hospital will be stored at the respective hospital servers and can be accessed or decrypted only by authorized users in the hospital environment. Data from the e-coach will exported with passwords per quarter, to the hospital pseudo-anonymized and stored on a secured server in an ITUMM-folder. Then all Data will be merged and recorded using digital data-collection forms from the hospital and the e-coach MM and will be entered in a research platform (research manager) for subsequent statistical analyses (SPSS). The data will be handled confidentially and pseudo-anonymously. Analyzed pseudo-anonymous results of the study are first shared internally with the research group and then discussed/shared with other stakeholders, especially the patients and relatives of the patients in a letter with an overview of the results and in a “mirror session” to discuss and explain the results with patients. In a “mirror session”, an independent facilitator invites 5 to 10 patients to share their experiences with an organization in a closed semicircle, while organizational representatives listen without engaging in discussion. The purpose is solely to gather feedback.

In this study, addressing missing data is essential to maintain the integrity of our findings. We will employ intent-to-treat analysis, encompassing all randomized participants irrespective of adherence, to uphold the principles of randomization and provide a conservative estimate of treatment effects. Additionally, per-protocol analysis will be conducted to evaluate treatment efficacy among strictly adherent participants, recognizing its potential for selection bias. Various methods, including imputation techniques and sensitivity analyses, may be utilized to handle missing data effectively and ensure the robustness of our conclusions.

#### Statistical analyses

- Descriptive statistics:

Categorical data will be presented as n (%). Continuous data will be presented as mean (standard deviation/ sd) or median (interquartile range) dependent on the distribution. Distributions will be assessed by plots (histogram) and Shapiro-Wilk tests.

- Inferential statistics:

The difference in pill count three months after randomization will be tested using linear regression with study arm and stratum as independent variables.

Concerning repeated measures, i.e. questionnaires, estimated means with 95% confidence intervals obtained by repeated measures mixed modelling will be plotted. For analyses, study arm will be evaluated as the primary determinant. The questionnaires (GFI, SDM-Q-9, EQ-5D-5L, EORTC-QLQ30, MY MARS-5) will yield numerical/continuous variables. Longitudinal courses of questionnaire outcomes will be visualized by plotting estimated means with 95% confidence intervals obtained by linear mixed model analysis. Linear mixed model analysis will be performed with time point as fixed effect. Covariance structures will be selected based on Akaike information criterion. A two-tailed alpha of 5% will be used with SPSS (IBM) version 28 and STATA (StataCorp) version 18.

#### Secondary study parameters and explorative other study parameters

Normally distributed continuous variables will be analyzed using the independent *t*-test. In case of non-normality in the data the non-parametric Mann-Whitney *U*-test will be used. The one-way analysis of variance (ANOVA) will be used to determine whether there are any statistically significant differences between the means of the four groups while adjusting for multiple testing.

## Supporting information

S1 Checklist (DOC)

S1 File (DOCX)
