# Supplementary material for: The impact of telemonitoring on correct drug use, complications and quality of life among patients with multiple myeloma (ITUMM): A study protocol for an open-label, parallel-group, randomized controlled trial
Source: PLoS One. 2024 Aug 26;19(8):e0307177. doi: 10.1371/journal.pone.0307177 (PMC11346735; doi:10.1371/journal.pone.0307177)
Supplement: S1 File — (DOCX) [file pone.0307177.s002.docx]

**RESEARCH PROTOCOL**

**The Impact of Telemonitoring on correct drug Use, complications and quality of life among patients with Multiple Myeloma (MM)**

**(ITUMM)**

A multicentre study in the Netherlands

**PROTOCOL TITLE** ‘The **I**mpact of **T**elemonitoring on correct drug **U**se, complications, and quality of life among patients with **M**ultiple **M**yeloma (MM)’

| **Protocol ID** | **ITUMM – study (The EudraCT number 2020-005267-31)**  **ABR number: NL75771.075.20** |
| --- | --- |
| **Short title** | **Randomized Controlled Trial of telemonitoring among patients with multiple myeloma** |
| **Version** | **10.0** |
| **Date** | **July 2021** |
| **Coordinating project leader(s)**  **And investigator(s)**  **Dutch: projectleider(s) en onderzoeker(s)** | ***Dr. P.G.J. ter Horst, Hospital Pharmacist***  ***Clinical Pharmacy Department, Isala Hospital***  ***Dokter van Heesweg 2, 8025 AB Zwolle***  ***Tel: 038-424-7244***  [***p.g.j.terhorst@isala.nl***](mailto:p.g.j.terhorst@isala.nl)  ***Dr. J. G. Maring, Hospital Pharmacist-Clinical Pharmacologist***  ***j.g.maring@isala.nl***  **Dr. P.A.F. Geerts, Internist-oncologist**  **Isala Oncology Centre**  **Dokter van Heesweg 2, 8025 AB Zwolle**  **Tel: 038-424-7039**  **p.a.f.geerts@isala.nl** |
| **Principal investigator (in Dutch: hoofdonderzoeker)** | ***J.F.H. Eijsink, Pharmacist and PhD-student***  ***Clinical Pharmacy Department, Isala Hospital***  ***Dokter van Heesweg 2, 8025 AB Zwolle***  ***Tel: 038-424-5693***  ***j.f.h.eijsink@isala.nl*** |
|  |  |
| **Co-investigators** | **Dr. J.L.L.M. Coenen , Internist-oncologist (because of retirement no longer be a practicing hematologist).**  **Dr. E. Kneppers, Internist-oncologist**  **Isala Oncology Centre**  **Dokter van Heesweg 2, 8025 AB Zwolle**  **Tel: 038-424-5116**  [**j.l.l.m.coenen@isala.nl**](mailto:j.l.l.m.coenen@isala.nl)  **e.kneppers@isala.nl** |
| **Co-investigators** | **Prof. Dr. Maarten Postma, UMCG sector F, health sciences**  **Dr. Cornelis Boersma, UMCG sector F, health sciences** |
| **Subsidising party** | ***Isala academy (I&W)***  ***Gebouw M (Mondriaan)***  ***Dokter van Deenweg 1***  ***8025 BP Zwolle*** |
| **Pharmacy** | ***Clinical Pharmacy Department, Isala Hospital***  ***Dokter van Heesweg 2, 8025 AB Zwolle***  ***Tel: 038-424-4255*** |
|  |  |

**TABLE OF CONTENTS**

[1. SUMMARY 7](#_Toc78459182)

[2. INTRODUCTION AND RATIONALE 9](#_Toc78459183)

[2.1 Multiple Myeloma (MM) 9](#_Toc78459184)

[2.2 Telemonitoring 10](#_Toc78459185)

[2.3 From concept to implementation 11](#_Toc78459186)

[2.4 Pilot study (F-ITUMM study) 12](#_Toc78459187)

[3. OBJECTIVES 13](#_Toc78459188)

[4. STUDY DESIGN 14](#_Toc78459189)

[4.1 Randomized Controlled Trial (N=150) 14](#_Toc78459190)

[5. STUDY POPULATION 15](#_Toc78459191)

[5.1 Inclusion criteria 15](#_Toc78459192)

[5.2 Exclusion criteria 16](#_Toc78459193)

[5.3 Sample Size Calculation 16](#_Toc78459194)

[6. NON-INVESTIGATIONAL PRODUCTS 17](#_Toc78459195)

[6.1 The e-coach MM 17](#_Toc78459196)

[6.2 Name and description of non-investigational product(s) 17](#_Toc78459197)

[7. METHODS 18](#_Toc78459198)

[7.1 General 18](#_Toc78459199)

[7.2 Primary outcome 18](#_Toc78459200)

[7.3 Secondary outcomes: (Appendix S3) 20](#_Toc78459201)

[7.4 Randomisation, blinding and treatment allocation 25](#_Toc78459202)

[8. Study procedures 26](#_Toc78459203)

[8.1 Plan 26](#_Toc78459204)

[Premature termination of the study 27](#_Toc78459205)

[9. Data analysis 28](#_Toc78459206)

[9.1 Primary study parameter: 28](#_Toc78459207)

[9.2 Secondary study parameters and explorative other study parameters 28](#_Toc78459208)

[10. ETHICAL CONSIDERATIONS 29](#_Toc78459209)

[10.1 Regulation statement 29](#_Toc78459210)

[10.2 Recruitment and consent 29](#_Toc78459211)

[10.3 Incentives (if applicable) 29](#_Toc78459212)

[11. ADMINISTRATIVE ASPECTS, MONITORING AND PUBLICATION 29](#_Toc78459213)

[11.1 Handling and storage of data and documents 29](#_Toc78459214)

[11.2 Amendments 30](#_Toc78459215)

[11.3 Safety reporting for Amgen products: 30](#_Toc78459216)

[11.4 Annual progress report 31](#_Toc78459217)

[11.5 Temporary halt and (prematurely) end of study report 31](#_Toc78459218)

[11.6 Public disclosure and publication policy 31](#_Toc78459219)

[12. APPENDICES 33](#_Toc78459220)

[12.1 S1 F-ITUMM Study (abstract) 33](#_Toc78459221)

[12.2 S2 Medication 34](#_Toc78459222)

[12.3 S3 Questionnaires 87](#_Toc78459223)

[12.4 S4 PROMs and PREMs 94](#_Toc78459224)

[12.5 S5 Clinical outcomes 95](#_Toc78459225)

[12.6 S6 Procedures 97](#_Toc78459226)

[12.7 S7 Pill count 100](#_Toc78459227)

[12.8 S8 Informed consent ITUMM study 102](#_Toc78459228)

[12.9 S9 Information letter ITUMM study for patients 102](#_Toc78459229)

[13. REFERENCES 105](#_Toc78459230)

**LIST OF ABBREVIATIONS AND RELEVANT DEFINITIONS**

| MM  RDMM  CAU  VBHC  EMR  EORTC-QLQc30 | | Multiple Myeloma  Recently Diagnosed Multiple Myeloma  Care As Usual  Value-Based Health Care  Electronic Medication Registration  European Organisation for Research and Treatment of Cancer quality of life physical functioning subscale |
| --- | --- | --- |
| EQ-5D-5L  MARS-5  SDM-Q-9  PDC  RCT  WHO  CEA  PFS  OS | | The 5-level EQ-5D version was introduced by the EuroQol Group in 2009 to improve the instrument’s sensitivity and to reduce ceiling effects.  Medication Adherence Report Scale-5  Shared Decision Making questionnaire-9  Portion Days Covered  Randomized Controlled Trial  World Health Organization  Cost-Effectiveness Analysis  Progression free survival  Overall survival |
|  |  |  |
|  |  |  |

# ****SUMMARY****

**Rationale:** Multiple myeloma (MM) is the uncontrolled division of malignant plasma cells in bone marrow tissue. It is the second most common haematological cancer in the Western world. About 1000 people in the Netherlands are diagnosed with MM each year [1]. There are around 4000 patients with this disease in the Netherlands. It mainly concerns people over 60 years old (80%) and there are slightly more men than women with MM [34]. A recent study has shown that the median survival varies from 37.5 months (95% confidence interval: 34.8–41.8 months) in the first line to 9.2 months (95% CI: 6.2–12.3 months) in the 4th line treatment regimens [2]. Although the intention to compliance, for complex MM drugs regimens, seems higher than other chronic diseases [3]. The complexity of the treatment regimens requires continuous effort and attention from the patient when taking the medication. An e-coach could possibly contribute to compliance [4,5].

**Objective**: The objective of this study is to compare the impact of a multicomponent self-management tool, using an e-coach *versus* care as usual. Using the MM e-coach may expected to increase patients compliance on correct drug use, improving the Quality of Life, reduce complications and MM related treatment costs.

**Endpoints:** The primary endpoint is compliance by pill count after 3 months, of recently diagnosed MM (RDMM) patients, started with an indication for a first-line or second-line treatment. Secondary endpoints are the compliance by pill count after 12 months , Persistence by Portion Days Covered (PDC), Medication Adherence Rating Scale (MARS-5), Quality of Life (EQ-5D and EORTC-QLQC30), Shared Decision Making (SDM-Q-9), Other explorative study parameters are: single items for MM specific Patient Reported Experience (PREMS), and MM specific Patient Reported Outcomes (PROMS), according to the concept of Value-Based Health Care (VBHC). (like single item “sexual functioning” or single item: “pain”), adverse events (grade III-IV), hospital admission related to the treatment of MM, hospital costs related to the treatment of MM. Additional explorative endpoints are; progression free survival (PFS) (36 months) and overall survival (OS) (at 5 years).

**Study design:** This study contains a multi centre, randomized controlled trial (RCT). RDMM patients scheduled for infusion- and oral-based (chemo) therapy will be included in the study. Patients will be randomly divided over two arms: the intervention group will be treated with e-coach assistance; the control group will receive care as usual.

**Study population:** Patients will be treated for MM according to local standard of care including supportive care (infection prophylaxis, thrombosis prophylaxis) in the first-line or second-line treatment. Patients will be screened on minimal digital skills to check if they are able/or can be trained to interact with an e-coach and whether they can read the Dutch language.

**E-coach:** The intervention consists of intensified support on correct intake of medication by using an e-coach combined with e-consulting. The e-coach consists of a medication assistance part coupled to the EMR/oncology prescription system, a diary part (to register disease burden and scores), e-learnings and it provides the possibility to connect directly with health care professionals (nurse specialist/physician assistant/medical oncologist/pharmacist) by e-consulting. Feedback to the professionals is given based on e-coach alerts, medication adherence questionnaire outcomes and pill counts, with lower potentially avoidable hospital admissions.

**Relevance for practice:**

There is potential and, from a safety perspective, medical need for multi-component intervention strategies to improve the medication adherence in patients treated with complex oral-based chemotherapy schedules.

The MM e-coach project must deliver a blueprint for future disease specific e-coaches to monitor complex pharmacotherapy at home.

- E-coach MM in a broader context (other hospitals and care outside the hospital)
- E-coaching in other diseases, with complex drugs regimes.
- A shift from second line (hospital) to first line (care at home and the general practitioner).

# INTRODUCTION AND RATIONALE

## Multiple Myeloma (MM)

MM is a malignant disease characterized by clonal proliferation of plasma cells. Plasma cells help you fight infections by making antibodies that recognize and attack germs. MM causes cancer cells to accumulate in the bone marrow, where they crowd out healthy blood cells. Rather than produce helpful antibodies, the cancer cells produce abnormal proteins that can cause complications. The myeloma cells continue trying to produce antibodies, as healthy plasma cells do, but the myeloma cells produce abnormal antibodies that the body cannot use. Instead, the abnormal antibodies (monoclonal proteins or M proteins) build up in the body and cause problems such as damage to the kidneys. Cancer cells can also cause damage to the bones that increases the risk of broken bones [3]. Signs and symptoms of MM can vary and, early in the disease, there may be none; bone pain, especially in the spine or chest, nausea, fatigue, frequent infections, weight loss and anemia.

Treatment for multiple myeloma is indicated when the disease is classified as symptomatic.

[35]. Although survival rates for MM have improved as treatment options have increased, the disease remains incurable. Daratumumab and/or carfilzomib, plus dexamethasone improves Progression Free Survival (PFS) in relapsed/refractory MM. Prototypic drugs thalidomide, bortezomib, and lenalidomide have each been approved for the treatment of this disease by targeting both MM cells and the bone marrow microenvironment. Although benefit was first shown in relapsed and refractory disease, improved overall response, duration of response, and progression-free and overall survival can be achieved when these drugs are part of first-line regimens. These expensive (oral) agents are widely used in MM patients because of their demonstrated efficacy in different lines of MM treatment. As indicated above, treatment regimens are complex and new MM (combination) regimens will be added in the coming years [40,41]. A first-line therapy is therefore not diverge in complexity from a second-line treatment, with the assumption that second-line patients have been aware of the disease and treatment options for a longer period of time, but on the other hand, patients may also already be familiar with comorbidity and related therapies that we also have no insight into [42,43]. A RDMM patient uses 10-15 oral drugs a day, including co-medication (a second medication used to alleviate the side effects of another).

Compliance to long-term therapy for chronic illnesses in developed countries is close to 50% [6,7]. Concerning oral anticancer agents, Bassan et al. reported compliance rates ranging from 40% to 100% [8]. Poor compliance not only affects the patient’s health, but it also puts financial strain on the healthcare system. The World Health Organization (WHO) identified incorrect intake or insufficient adherence to therapy as one of the main causes of morbidity, mortality and the increasing health costs in the world [9]. It is estimated that between 30-50% of patients, depending on the disease and health system, do not use the medication as prescribed [10-14].

A pilot study from Mauro et al. assed the effect of an intervention on medication adherence in patients with multiple myeloma that are new to lenalidomide therapy. described that sixteen participants in each arm completed the study; 4 patients in each arm were lost to follow-up. Median adherence by a smart pill bottle was improved for the intervention group compared with the control group (median = 100% vs. 87.4%; P = 0.001) a difference of +/- 10% [38].

In absence of large adherence studies in MM we have also viewed at adherence to oral tyrosine Kinase inhibitor (TKI’s) in chronic Myeloid Leukemia (CML). In CML the subject of adherence has been studies extensively. A recent publication by, Tran et al. improved adherence by CML patients adherence to TKI as well as achieved better clinical outcomes by medication management service. In addition, a significantly higher proportion of participants in the intervention group achieved major molecular response at 6th month (58.5% vs 35.9%; p = 0.010), but not at 12th month (66.2% vs 51.6%; p = 0.092) [39].Incidence

In 2016, there were about 130,000 cases of MM in the worldwide translating to an age-standardized incidence rate of 2.1 per 100,000 persons. Incident cases from 1990 to 2016 increased by 126% globally [15]. MM caused 98,437 deaths globally a year, with an age-standardized incidence ratio of 1.5 per 100,000 persons. In the Netherlands prevalence of MM is 4000. The incidence in Isala is 50-75 patients a year and a prevalence in Isala of 250 patients [1]. It mainly concerns patients over 60 years old (80%) and there are slightly more men than women with MM. A study [3] has shown that the median survival varies from 37.5 months (95% confidence interval: 34.8–41.8 months) in the first line to 9.2 months (95% CI: 6.2–12.3 months) in the 4th line treatment regimens.

In Europe, the age-standardized incidence of MM has been reported to be approximately 5 cases per 100,000. The median age of patients at diagnosis is approximately 65–70 years with 37% of patients being younger than 65 years of age. MM is extremely rare in those less than 30 years of age with a reported frequency of 0.02% to 0.3% and appears to occur slightly more frequently in men. In general, MM is not considered to be a genetic disease.

## Telemonitoring

There is an ongoing effect towards shifting care to the outpatient setting following intensive cancer therapy. Telemonitoring allows a clinician to monitor patient reported outcomes, on a time dependent basis, physiological variables could also be measured in the home setting. This provides a means to keep patients with MM under close supervision, which could improve compliance, reduce the rate of admission to hospital and accelerate discharge. Interest in telemonitoring as a viable alternative for the provision of care has been stimulated by the rising costs of care in hospital, rapid advances in communication and diagnostic technology and the wider availability of low‐cost, patient‐friendly telecare equipment [4,5]

The e-coach consists of a medication assistance part coupled to the electronic medical records (EMR) oncology prescription system, a diary part (to register disease burden and scores), e-learnings and it provides the possibility to connect directly with health care professionals (nurse specialist/physician assistant/medical oncologist/pharmacist) by e-consulting. Specific patient reported outcomes (PROs) are used in dashboards for shared decision making [19,20].

Patients are involved in the development and experiences of the e-coach. Patients are also involved in a value-based healthcare (VBHC) cycle in Isala, with a new clinical care pathway for MM as a result *(Figure 1)*.


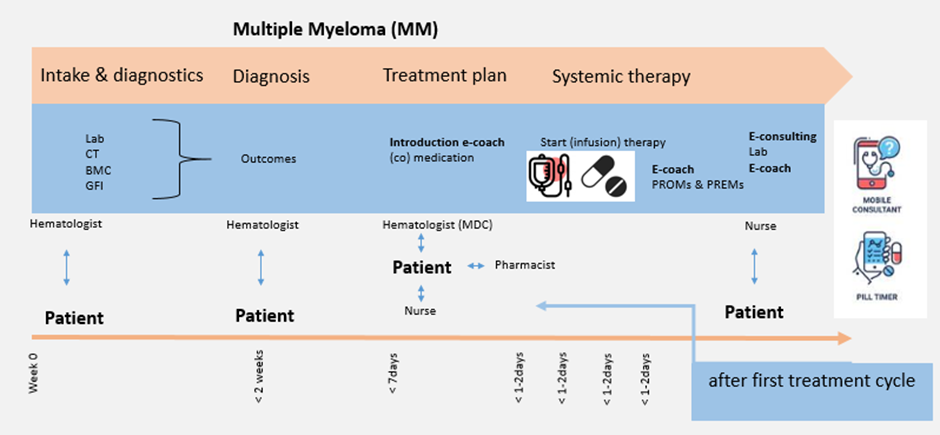


***Figure 1.*** *New clinical care pathway for multiple myeloma in Isala, the Netherlands. Designed with an innovative perspective, “care at home and if necessary in the hospital”. Lab: laboratory results: M-protein, hemoglobin, urine, kidney function, cytogenetics; CT: computed tomography; BMC: bone marrow centesis; GFI: Groninger frailty index; MDC: multidisciplinary consultation; PROMs: patient reported outcomes measures PREMs: patient reported experiences measures*

## From concept to implementation

The concept of value-based healthcare (VBHC) is from volume driven healthcare to patient value. Two aspects are important for VBHC, first outcomes and second patient participation. First, VBHC outcomes will have to be defined for a specific disease, with the approach align with patient-centered care, including the use of well-defined outcome measures. Patient-reported outcomes (PROs) expressed in patient-reported outcome measures (PROMs) and patient-reported experiences measures (PREMs) are leading in the concept, besides clinical outcomes. PROs are leading to shared decision making and patient-centered care. Second, patient participation is necessarily needed in the cycle of care to define PROs, to capture the patient’s perspective. Participation means an equal and proactive role in a multidisciplinary team of healthcare professionals. Not surprisingly, it is conceivable that PROs, but also the participation of patients is important to actually able to implement the concept of VBHC in practice.

A focus group consisting of health care professionals and patients has defined patient reported outcomes/experiences and clinical outcomes from the perspective of value-based healthcare for MM patients (5 sessions of 3 hours), relevant patient reported measurements (PROMs and PREMs in Appendix S4.) are defined as summarized below (Figure 3). [16-20]


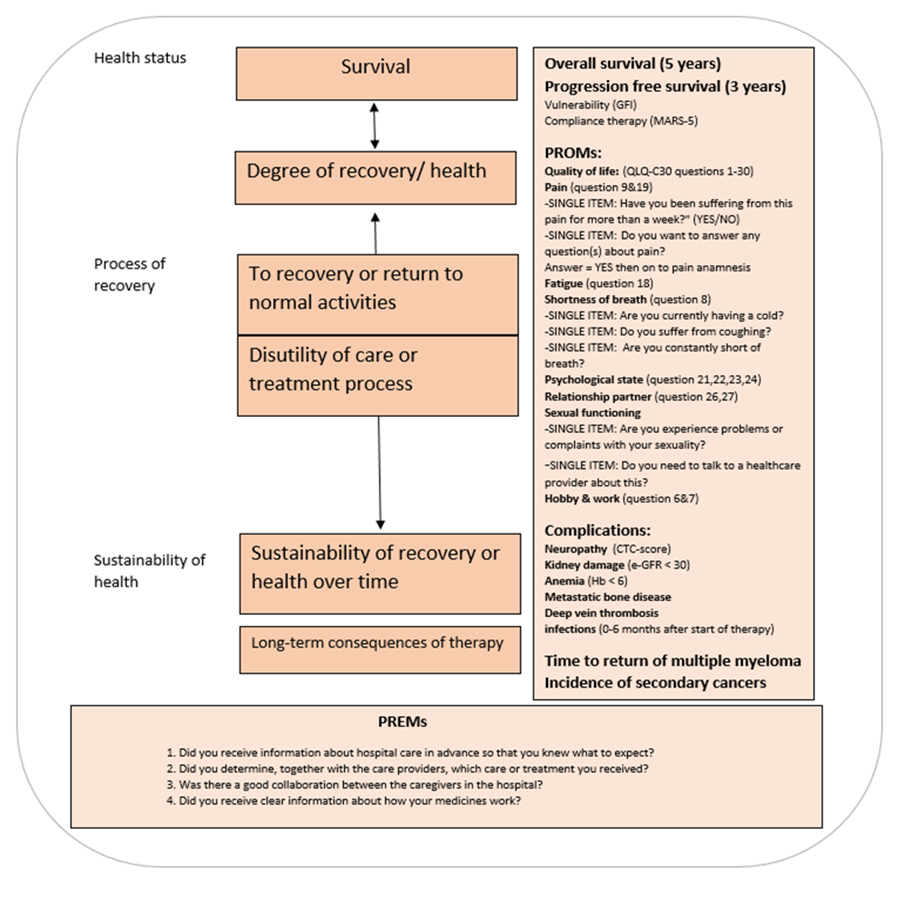


***Figure 2.*** *The results of the Patient Reported Outcome Measures (PROMs) and Patient Reported Experience Measures (PREMs) of the multiple myeloma Value-Based Health Care (VBHC) cycle in Isala, according to the concept of prof. Michael Porter. All PROMs and PREMs are integrated in the e-coach as questionnaires. The clinical outcomes, complications and survival data will be collect from the electronic patient files. :PFS is defined as the time from randomization in the ITUMM study to the date of death due to any cause, or to the date of censoring at the last time the subject was known to be alive in intention-to-treat populations. OS is “an unambiguous endpoint measure because it is evaluated on a continuous time scale, which gives precise accuracy for the time of the event.*

## Pilot study (F-ITUMM study)

After the implementation of the VBHC outcome set (*Figure 2)* in Isala and the development of the e-coach, a pilot study is performed, 3-4 months before start of the RCT study in collaboration with 20 patients in Isala (Appendix S1). In week 0 and week 8 all PROMs / PREMs were questionnaires asked via the e-coach from patients (n=20) and results are presented in a feasibility study. Results are also used for statistical analysis in the ITUMM protocol, for example, the SD for Quality of Life in the statistical analysis calculation. Further al functional outcomes (registrations) will be examined and evaluated.

An evaluation with the developer (Sananet), the hospital (Isala) and Amgen, covering all possible outcomes is scheduled in the last week of September 2020. There the stakeholders will determine if further development is needed or if the RCT can actually start in Q4 of 2020. The go-no-go criteria are that the e-coach is technically functional, patients and caregivers are able to use it, and that all data required for the RCT can be collected correctly. The F-ITUMM study will be presented at the ISPOR 2020 and ICHOM congress 2020.

# OBJECTIVES

The main objective of the project is to study difference between compliance by pill count after 3 months by recently diagnosed multiple myeloma (RDMM) patients with an innovative e-coach, in order to improve treatment compliance by the intervention group compared to the control group. Secondary objectives:compliance by pill count after 12 months, medication persistence (PDC), MARS-5, evaluate Quality of Life after 12 months, improved Shared Decision Making, PROMs and PREMs, Progression Free Survival, Overall Survival, adverse events, hospital admissions (hospitalization linked to oncology treatment) and hospital costs. 10% difference in compliance between intervention group (e-coach MM) versus control group (care as usual) is feasible and clinically meaningful.

**Hypotheses**

Primary hypothesis

We hypothesize that implementation of the e-coach MM will result in:

-Improved treatment compliance of minimum 10 percent by pill count after 3 months

**Secondary hypotheses**

-Improved compliance by registration pill count after 12 months in e-coach

-Medication persistence after 12 months

-Improved Quality of Life after 12 months

(time to 10 point change will also be estimated and compared.)

-Improved Shared Decision Making after 12 months

**Other explorative parameters**

-Reduced hospital costs related to the treatment of MM

-Reduced adverse events (grade III-IV)

-Improved PFS and OS

-Examine the consistency of the e-coach across the subgroups (FIT and NOT FIT).

Intervention

The intervention consists of intensified support on correct intake of medication by using an e-coach combined with e-consulting compared to care as usual. The e-coach consists of a medication assistance module coupled to the EMR/oncology prescription system, a diary (to register disease burden and scores), e-learnings and it provides the possibility to connect directly with health care professionals (nurse specialist/physician assistant/medical oncologist/pharmacist) by e-consulting. Feedback to the professionals is given based on e-coach alerts, medication adherence questionnaire outcomes and pill counts, with lower potentially avoidable hospital admissions. Specific patient reported outcomes are used in dashboards for Shared Decision Making.

# STUDY DESIGN

## Randomized Controlled Trial (N=150)

This is a multi-site RCT in 150 MM patients to study differences in compliance by pill count, and evaluate: compliance by pill count after 3 months and after 12 months of treatment, medication persistence (PDC), MARS-5, evaluate Quality of Life, improved Shared Decision Making, PROMs and PREMs, Progression Free Survival, Overall Survival, adverse events, hospital admissions (hospitalization linked to oncology treatment) and hospital costs *(Figure 2).*

Every RDMM patient qualifies for study participation. Multiple Myeloma patients scheduled for treatment with I.V., S.Q. -and oral-based (chemo) therapy will be recruited and selected on fit (18-70 years) or not fit (> 70 years) iDoc protocol 110769 and is conform the HOVON guideline. A FIT patient is eligible for stem cell transplantation, which affects the total treatment options and survival rate compared to a NOT FIT. FIT versus NOT FIT is mainly determined by age as determined in the Dutch HOVON Multiple Myeloma Guideline. This guidance is also used in treatment protocols in Dutch hospitals. The threshold to be eligible for autoHSCT is younger than 70 years of age according to this guideline. In addition, a frailty index score has been included in the RCT, which is planned in a multidisciplinary meeting shortly after a diagnosis. In the RCT the age limit of 70 years is used with a high frailty index as “not fit”, all patients who are younger and score well on the frailty index, are therefore eligible for a bone marrow transplant and more options with regard to medication. It is important to mention that a care provider and in particular the multidisciplinary meeting is important, in this case the team can decide whether a patient who is on the limit of the criteria can be treated in the fit group.

Every potential participant of the study is eligible for first-line or second-line treatment. Second-line treatment is treatment for a disease or condition after the initial treatment (first-line treatment) has failed, stopped working, or has side effects that are not tolerated. After the patients will be treated for MM according to local standard of care including supportive care (infection prophylaxis, thrombosis prophylaxis). Patients will be screened on minimal digital skills by the healthcare professional, to check if they are able/or can be trained to interact with an e-coach and whether they can read the Dutch language. Patients will enter the new care path to the study for 12 months. Based on the inclusion criteria we expect +/-100 recently diagnosed patients each year, assumed that 50% of this group is willing to participate in the study, we arrive at a conservative approach of +/-50 patients a year. After 12 months follow-up per patient, we will evaluate if the preliminary results contain the expectations and make a shift from the RCT to care as usual with integration of the e-coach in the care pathway.

***
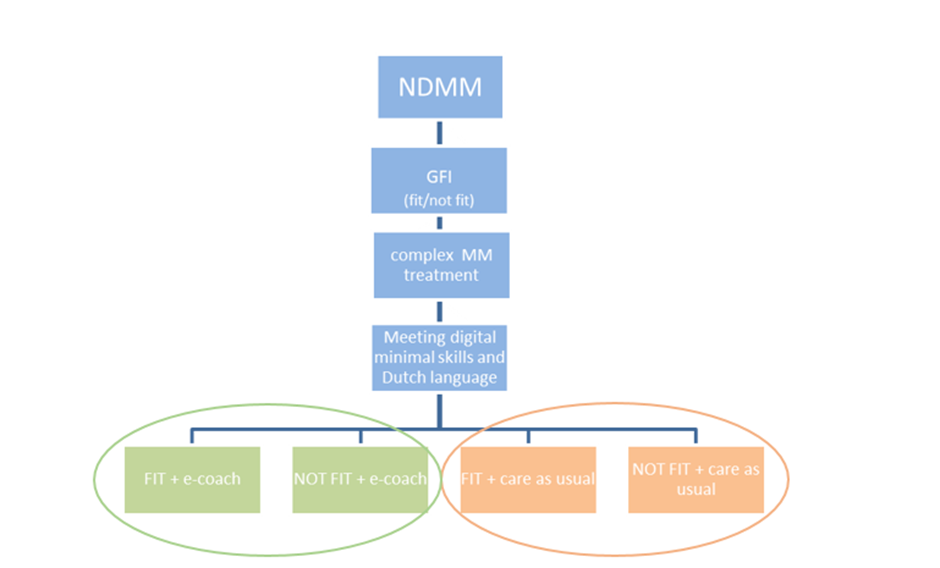
***

***Figure 3*.** *The flow of the ITUMM-study, with the intervention arm (green) and the control arm (orange). RDMM: Recently Diagnosed Multiple Myeloma patients. GFI: Groninger Frailty Index. FIT: patients who are eligible for stem cell transplantation according to protocol of the HOVON. NOT FIT: patients who are not eligible for stem cell transplantation according to the guidelines of the HOVON. FIT versus NOT FIT patients will be distributed equal over the two groups: intervention group versus control group. The primary analysis will compare the e-coach group versus the care as usual (non-e-coach) groups.*

**Study setting and participant selection**

This multi-site study will be conducted at a regional cancer center (referred to as ‘het oncologisch centrum Isala’), which provides non-surgical specialist cancer services to a diverse population of 1.5 million people in the east of the Netherlands.

# STUDY POPULATION

## Inclusion criteria

In order to be eligible to participate in this study, with informed consent, a subject (patient) must meet all of the following criteria:

Patients meeting any of the following criteria may be included in the study:

- Females and males > 18 years;
- Diagnosis of multiple myeloma (RDMM patients);
- Patients with a first-line or second-line MM treatment, with oral (co)medication;
- Able to complete outcome measures;
- Patients will be screened on minimal digital skills to check if they are able to interact with an e-coach and whether they can read and understand the Dutch language; estimated by the patient’s haematologist/oncologist
- Patients have their own mobile (smart)phone, computer or tablet (20 iPads are available for exceptions)
- Every recently diagnosed multiple myeloma patient qualifies for study participation.

**During inclusion an overview will be made of**

• Percentage yes/no participation

• Patient characteristics (social economic status/ age/weight/fit/not fit)

• Reason not to participate

## Exclusion criteria

A potential subject who meets any of the following criteria will be excluded from participation in this study:

- Not prescribed oral medication for a MM treatment regime;
- A third-line treatment or follow-up MM treatment;
- Psychiatric illness requiring secondary care intervention;
- Too ill to engage with the intervention in the opinion of the clinical care team, no perspective of 12 months or longer to survive.

## Sample Size Calculation

**Primary endpoint: compliance by pill count at 3 months**

The sample size of this study is based on the primary aim of this study, i.e. demonstrating superiority of “the coach” concerning pill count at t=3 months after randomization. Pill count is a continuous measure expressed in percentages and has a normal distribution. Duration of the trial is 2 years (50-70 recently diagnosed MM patients each year). We used a conservative approach for the sample size calculation, and this is based on an unpaired t-test, because of the unpredictability of the disease and moreover the vulnerability of this group of patients.

Furthermore, the number of elderly patients with MM will probably increase because of the improved survival rates that are associated with novel agents coupled with the increasing life expectancy of the general population. The age-related changes in physiology combined with comorbid conditions, disability, or frailty have important implications for the treatment of patients with cancer. Vulnerability, disability, comorbidity and frailty are factors who could have an impact on our inclusion rate, therefore we calculated a conservative sample size.

At t=3 months we hypothesized that there will be a minimum significant difference of 10% between the control group and intervention group, in compliance by pill count.

**Sample size calculation** was performed using SPSS SamplePower3.0. Calculation for pill count was based on the unpaired t-test, minimal clinically relevant difference of 10% [26,27], standard deviation 20% [29], power 80%, and 2-tailed alpha 5%. This calculation results in n=64 patients in each arm. Taking into account an attrition rate of 15%, yields n=75 patients to be included in each arm. Therefore, in total n=150 patients will be included in this study.

**Secondary endpoint: Quality of Life (EORTC-QLQ-30) at 12 months**

The F-ITUMM study showed at t=0 a mean of 69,7 +/- SD of 16,5 on Quality of Life, we assumed that this is a very comparable group of patients [32], to NDMM patients in Isala. As suggested before we will prove a minimal clinical difference of 10 points between the control and intervention group. [32]

**Sample size calculation** based on unpaired t-test, for the given effect size (population means of 69,7 vs. 79.7), SD (16,5), alpha (0,050, 2-tailed), power is 80% , resulting in n=44 patients per research arm.

# NON-INVESTIGATIONAL PRODUCTS

## The e-coach MM

SananetOnline is an ISO9001/ ISO27001 / NEN7510 certified, patient-centered-care support recognized, medical self-care platform on which e-coaches are developed and deployed for (chronically) ill patients as part of the treatment together with care professionals and can be integrated with Hospital / GP Information Systems. SananetOnline has a CE marking class 1 from the European Union.

The e-coach MM is an eHealth application that is intended to provide remote support for patients with multiple myeloma to take their medication on time and according to prescription, thus preventing medication errors such as over- and under-dosing as much as possible. Early detection of increasing complaints will enable caregivers to apply the correct intervention in a timely manner. These factors are expected to optimize the outcome of the treatments.

## Name and description of non-investigational product(s)

Appendix S2

# METHODS

## General

Firstly, the e-coach was developed, from September 2019 to June 2020. Secondly, a feasibility study (F-ITUMM trial) was conducted. From June 2020 to Augustus 2020. Followed by the randomized controlled trial (RCT), from November 2020 to November 2022. Further optimization of the e-coach after the study is scheduled. After 1 year an interim analyses is scheduled. After 1 year an interim analyses is scheduled. The interim analysis is conducted before data collection has been completed. If the e-coach can be proven to be clearly beneficial or harmful compared to the control arm, the investigators may stop the study early

**Patient reported results** are expressed in the PROMs for MM: (EORTC-QLQ-C30 and single items): *quality of life, pain, fatigue, shortness of breath, psychological state, relationship partner, sexual function, hobby and work*. PREMs expressed in *information and expectation about hospital care, shared decision making, collaboration and information about medication* are defined in standardized questions from the patient satisfaction survey in the hospital. Clinical outcomes, *like mortality (progression free survival: PFS and overall survival: OS), neuropathy, kidney damage, anemia, metastatic bone disease, deep vein thrombosis and infections.* *The vulnerability (Groninger Frailty Index: GFI) and compliance (Medication Adherence Rating Scale: MARS-5)* are also important for the patient and the care pathway they will run through.

## Primary outcome

**Medication compliance by pill count after 3 months therapy**

In this study we defined the primary outcome, translated to compliance of the MM oral therapy (backbone therapy plus co-medication) in the first-line or second-line. We hypothesized that a minimum clinical significant difference of 10 percent between de intervention arm and care as usual arm is plausible after three months, from diagnosis [9-14]. Moon et al. and Mauro et al. showed results that support this assumption in a hematological group of patients with oral treatment. They showed a compliance difference between 13% and 17% in compliance in a intervention group versus a care as usual group in medication guidance [26,27]. Lee et al. showed that pill count is an accurate method compared to twenty-four hour recall and refill history [28]. Claxton et al. showed general compliance outcomes around 70% (+/- 17%), and declined as the number of daily doses increased [29]. Specific compliance outcomes for oral oncolytics are around 30-50% incorrect use of medication [7,8]. The World Health Organization (WHO) defines adherence as the extent to which a person’s behavior in taking medication or making lifestyle changes agrees with recommendations from a healthcare provider. Medication compliance (synonym: adherence) refers to the act of confirming to the recommendations made by the provider with respect to timing, dosage and frequency of medication taking. Compliance is measured in this study over a time period of 3 months and reported as a percentage *(Figure 4)*.

Patient awareness that compliance is being measured may impact the degree of compliance because patients may demonstrate improved behavior. Self-report questionnaires could result in over-reporting rates of compliance because of a desire to please providers. Pill counts requiring patients to return unused pills at each visit so that the number of missed doses can be calculated, could potentially lead to throw away missed doses to avoid being viewed as non-complaint. Pill counts provide no information concerning the timing of doses, in this study the coach records the timing of doses. We do not choose for the microelectronic monitoring system (MEMS), because a pill may not be ingested every time the pill bottle is opened. Even with these more objective measurements, data may still be influenced by the effect of improved behavior. The MEMS technique is quite expensive and not feasible in our study, with many complex medication schedules for multiple myeloma. Other studies suggest a preference for pill count, instead of MEMS [36].

The pill count methodology avoids the effect of improved behavior, because patients would generally not be aware that their refill rates would eventually be reviewed at the pharmacy. Patients will be informed by a information letter about pill count. Besides,compliance to therapeutic MM regimens will be increasingly important in medical oncology as additional oral treatments are adopted for use in MM care.


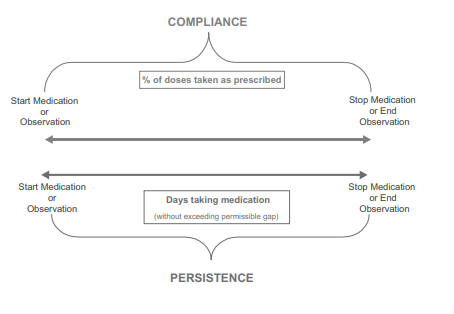


***Figure 4.*** *Difference in compliance and persistence*

**Hypothesis:** 10% improvement in compliance by intervention of an MM e-coach compared to standard of care, which have effect on:

- behavior of patients, consult in the hospital
- education about medication
- reminder/monitoring of medication intake
- personal factors (emotional state, health beliefs, social support, socioeconomic status)

**Compliance as a direct measurement:**

- Direct observation of the behavior (in daily practice, could not be measured)
- Monitoring electronic records, (12 months in the coach for the intervention group)
- Delivery / prescription / insurance information; all based on routinely collected care records, (for intervention and control group, 12 months PDC)
- Pill counts (3 months for both groups)
- Self-report: interviews / questionnaires / diaries. (PROMs = MARS-5 for both groups)

Poor compliance can result in: drug resistance, which is a reduction in how well a drug will work in curing or controlling a disease or condition, poor response to therapy or progression of the disease, which lead to increased doctor visits, lab tests, and hospitalizations.

After start of the study, we will measure the compliance by pill count at 30-60-90 days. Participants of the ITUMM study will get a “medication bag”, after every hospital visit, with the oral therapies and a first dispensing instruction form the pharmacist [37]. After that, the patient will go to the research nurse for a consultation hour and introduction for the e-coach (a login on the e-coach) and all medication is predefined in the coach with all different timeslots of medication intake. After a month the patient get a new medication prescription and he/she will come to the hospital (pharmacy) for the 2^nd^ dispensing of medication. At every prescription time the patient get a medication back and the other back will be counted with a pill count format Appendix S7. We will measure the differences in percentage of compliance, between 30 days, almost immediately after diagnosis and a few cycles of therapy, after 3 months. Compliance will be defined as the percentage of correct dose intakes.

**Compliance per patient**

Month 1: 100%- ((missed doses / divided total count of pills) *100) = compliance month 1 (30 days)

Month 2: 100%- ((missed doses / divided total count of pills) *100) = compliance month 2 (60 days)

Month 3: 100%- ((missed doses / divided total count of pills) *100 = compliance month 3 (90 days)

**Overall compliance** (%) **= (month 1 + 2 + 3) / divided 3))**

**Compliance difference**

Month 1 = +/- 30 days (intervention group) vs. Month 3= +/- 90 days (intervention group)

**= difference intervention group**

Month 1 = +/- 30 days (control group) vs. Month 3 = +/- 90 days (control group)

**= difference control group**

**Difference intervention group – difference control group = compliance difference in 3 months**

## Secondary outcomes: (Appendix S3)

Overview of secondary outcomes:

- Compliance by pill count after 12 months. Medication Persistence (PDC)
- Medication Adherence Rating Scale (MARS-5)
- Quality of life (EQ-5D-5L), for a CEA in the future
- Quality of life after 12 months (EORTC-QLQ-C30 version 3), comorbidities that influence quality of life for MM patients are e.g. bone pain, anaemia, fatigue, infections, impaired renal function and spinal cord damage
- Shared decision-making (SDM-Q-9 Dutch), during the study period.
- MM specific Patient Reported Experiences, Figure 6. (PREMS)
- MM specific Patient Reported Outcomes, Figure 6. (PROMS)

-Pain

-Neuropathy

-Fatigue

-Shortness of breath

-Psychological state

-Relationship partner

-Sexual functioning

-Hobby & work

Other explorative parameters at baseline and during the study (Appendix S5)

- PFS, Progression free survival (36 months)
- Mortality (after 60 months)
- Incidence adverse events (grade III and IV)
- Hospital admissions and costs (hospitalization linked to oncology treatment)
- Anaemia (hemoglobin)
- Kidney damage (eGFR)
- Metastatic bone disease
- Deep vein thrombosis
- Infections

**Persistence (Proportion of Days Covered)**

PDC calculates the ratio of number of days the patient is covered by the medication in a period to the total number of days in the period. Therefore, PDC can also calculate the number of days the patient is covered by multiple medications in a period by the total number of days in the period. The calculation of the PDC is used in multi-therapies.

In a study from Mian et al. compliance was measured as medication possession ratio (MPR), which was defined as the ratio of the number of days the patient had pills in their possession to the number of days in the observation period in the first year after myeloma diagnosis [30]. MPR of < 90% was considered poor adherence. Over a third of older adults with recently diagnosed multiple myeloma were considered to have poor compliance to lenalidomide, using the MPR as a surrogate for adherence.

Persistence may be defined as “the duration of time from initiation to discontinuation of therapy”. Persistence is reported as a continuous variable in terms of number of days for which therapy was available *(Figure 5).* Persistence may also be reported as a dichotomous variable measured at the end of a predefined time period (12 months), considering patients as being persistent or non-persistent. Addressing both compliance and persistence provides a richer understanding of medication-taking behavior.

For this study we could expect a high outcome on non-persistence, because of the prescription changes over time for each individual. Non-persistence is defined as discontinuation of the MM therapy following an allowed gap between refills – 30, 60, 90 days. For this study we include NDMM patients with oral treatment. The proportion of days in the measurement period "covered" by prescription claims for the same medication or another in its therapeutic category, we therefore distinguish two groups of medication, backbone therapy (effects on survival rate) and co-medication (assisted therapy). The persistence can be calculated with the extraction of retail pharmacy data in Isala. PDC is recommended for assessing the medication compliance of patients on multiple therapies at the same time. PDC calculates the ratio of number of days the patient is covered by the medication in a period to the total number of days in the period. Therefore, PDC can also calculate the number of days the patient is covered by multiple medications in a period by the total number of days in the period.

**Medication persistence is measured by an indirect method**

Proportion of Days Covered (PDC) per patient **(backbone therapy)**

Month 1: (N days in period covered / N days in the month) * 100%

Month 2: (N days in period covered / N days in the month) * 100%

Month 3: (N days in period covered / N days in the month) * 100%

Etcetera

**Total mean PDC: Month (1+2+3+ 4+5+6+7+8+9+10+11+12) / divided 12 months))**

Proportion of Days Covered (PDC) per patient **(co-medication)**

Month 1: (N days in period covered / N days in the month) * 100%

Month 2: (N days in period covered / N days in the month) * 100%

Month 3: (N days in period covered / N days in the month) * 100%

Etcetera

**Total mean PDC: Month (1+2+3+ 4+5+6+7+8+9+10+11+12) / divided 12 months))**


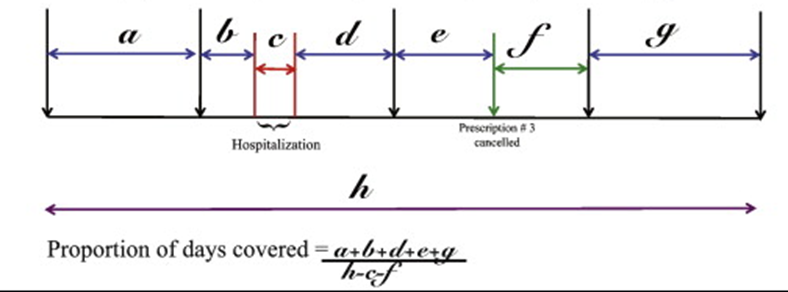


***Figure 5. Proportion of Days Covered***

**Medication Adherence Rating Scale (MARS-5)**

A secondary outcome is the patient reported MARS-5 score. For compliance, reported by patients themselves resulted in a higher percentage by Immunomodulatory Drugs, also used by MM treatment. Another systematic review about adherence in patients with haematological malignancies reports adherence rates between 20 and 53% in patients with chronic myeloid leukaemia (CML) and non-adherence rates of 6–35% in patients with acute lymphoid leukaemia (ALL) [7]. A score of >23 by the MARS-5 is therapy compliance.

The MARS-5 consists of 5 items describing non-adherent behaviours (“I forget to take the medicine / I alter the dose of medicine / I stop taking the medicine for a while / I decided to miss out a dose / I take less than instructed”): patients are asked to evaluate how often they adopt each behaviour with a 5 point scale, ranging from “always” to “never” (1–5 points). The scale total score ranges from 5 (lowest adherence) to 25 points (maximal adherence).

Medication non-compliance is associated with poor health outcomes and increased health care costs. In malignancy, non-compliance reduces chances of achievement of treatment response and may thereby lead to progression or even relapse.

Besides, we take into account the effect of participation in the trial and its effect on compliance. The HOMERUS study describes, participation in the study significantly increased adherence from 90.6 to 95.6% (p <0.001). After the study period, treatment adherence to the medication of the HOMERUS study decreased to 91.8% (p <0.001), which was no different from adherence before the start of the clinical study (p = 0.45). Adherence to other non-study-related medications also increased from 77.6 to 89.6% (p <0.001) as a result of study participation [31].

We assumed that patients report better than they actually do (primary endpoint), that they also score high, therefore we formulate the hypothesis: Patients do not score worse on self-reported compliance (from t= 0) with an e-coach compared to care as usual after 3 months of treatment (other data points continue t = 6,9,12 months).

**Quality of Life (EQ-5D-5L and EORTC-QLQC30)**

In this study we use two patient reported Quality of Life scores. Quality of life (QOL) is important in patients with advanced cancer. The European Organisation for Research and Treatment of Cancer (EORTC) QLQ-C30 is a general QOL tool used in cancer patients. The EORTC QLQ-C30 comprises 30 items (i.e. single questions), 24 of which are aggregated into nine multi-item scales, that is, five functioning scales (physical, role, cognitive, emotional and social), three symptom scales (fatigue, pain and nausea/vomiting) and one global health status scale. The remaining six single-item (dyspnoea, appetite loss, sleep disturbance, constipation, diarrhoea and the financial impact) scales assess symptoms. All of the scales and single-item measures range in score from 0 to 100. Higher score for the functioning scales and global health status denote a better level of functioning (i.e. a better state of the patient), while higher scores on the symptom and single-item scales indicate a higher level of symptoms (i.e. a worse state of the patient).

Timilshna et al. reported a minimum significant difference of 10 points per item of the QLQ-C30 at t = 0 and t = 12 months (other data points for our study continue at t = 3, 6, 9 months) [32]

We therefore assumed a minimal difference of 10 points on each domain score of the EORTC-30 at t= 0 and t = 12 months between control and intervention group. (time to 10 point change will also be estimated and compared.)

The EORTC QLQ-C30 is a more general QOL instrument designed for patients with cancer [8]. It contains five functional scales (physical, role, cognitive, emotional, and social), three symptom scales (fatigue, pain, and nausea and vomiting), a global health status/QOL scale, and a number of single items assessing additional symptoms commonly reported by cancer patients (dyspnea, loss of appetite, insomnia, constipation, and diarrhea) and perceived financial impact of the disease. In total there are 30 items on the questionnaire. Each item in the EORTC QLQ-C30 is rated from 1 (not at all) to 4 (very much) in severity, except for the overall QOL scale, which is rated from 1 (very poor) to 7 (excellent).

The EQ5D-5L comprises five dimensions: mobility, self-care, usual activities, pain/discomfort and anxiety/depression. Each dimension has 5 levels: no problems, slight problems, moderate problems, severe problems and extreme problems. The EQ5D-5L is asked at t=0 and 12 months from the e-coach. We will use the EQ5D-5L results, for a cost-effectiveness analysis, after the ITUMM-study.

S**hared Decision Making (SDM-Q-9)**

(SDM) has been defined as: ‘an approach where clinicians and patients share the best available evidence when faced with the task of making decisions, and where patients are supported to consider options, to achieve informed preferences’. The nine-item SDM Questionnaire (SDM-Q-9) is one of the most frequently applied instruments for assessing patients involvement in medical decision-making. We suggest that there possibly could be a difference in SDM between the intervention group and control group. In this trial the healthcare provider informs the patient that a decision is to be made and that the patient’s opinion is important. Here emphasis is on the patient and healthcare provider working together as a team. At the option talk step, the healthcare provider explains the options, amongst whom to option to do nothing, and the pros and cons of each relevant option. Subsequently these options can be compared to each other. The final step, decision talk, includes patient preference elicitation and making the decision. Stiggelbout et al. prefer to split the decision talk in two steps: first, the professional and patient discuss the patient’s preferences and the professional supports the patient in deliberation. Second, the professional and patient discuss the patient’s decisional role preference, make or defer the decision, and discuss possible follow-up [23].

The SDM-Q-9 is a self-reported questionnaire designed to assess patients’ views on SDM occurred in a consultation with a healthcare provider. It contains two open-ended questions [‘Please indicate which health complaint/problem/illness the consultation was about’ and ‘Please indicate which decision was made’] followed by nine closed questions. Each closed question is represented by a statement featuring various aspects of SDM, rated on a 6-point balanced scale ranging from 0 (= ‘completely disagree’) to 5 (= ‘completely agree’). The total score, calculated by summing the score of the nine items, is expressed on a scale ranging between 0 and 45, where a higher score represents a greater level of perceived SDM. The SDM-Q-9 is asked at t=0, 6 and 12 months form the e-coach. We will use the SDM-Q-9 as an explorative outcome in this study.

Further, we will study explorative parameters: Progression Free Survival (36 months), Mortality (after 60 months), number of adverse events (grade III and IV) and number of hospital admissions (hospitalization linked to oncology treatment). Anaemia (hemoglobin), kidney damage (eGFR), metastatic bone disease and deep vein thrombosis.

Repeated measures mixed modelling will be used for questionnaires that are repeatedly administered, with study arm as primary determinant. The measures start at t=0 (diagnosis of MM).

**MM specific Patient Reported Experience (PREMs)**

PREM questionnaires are patient reported experiences and are asked at t=0, 6, 12 months from the e-coach. The questionnaire included 4 items concerned with experience. PREMs are: Information from the hospital, shared decision-making, Collaboration between hospital practitioners, Information about the use of medication (*Figure 6.)*

**MM specific Patient Reported Outcomes (PROMs)**

PROM questionnaires are patient reported outcomes and are asked at different times over the year, dependent on the possible side effects of MM treatment and psychological/social aspects. *(Figure 6.)*


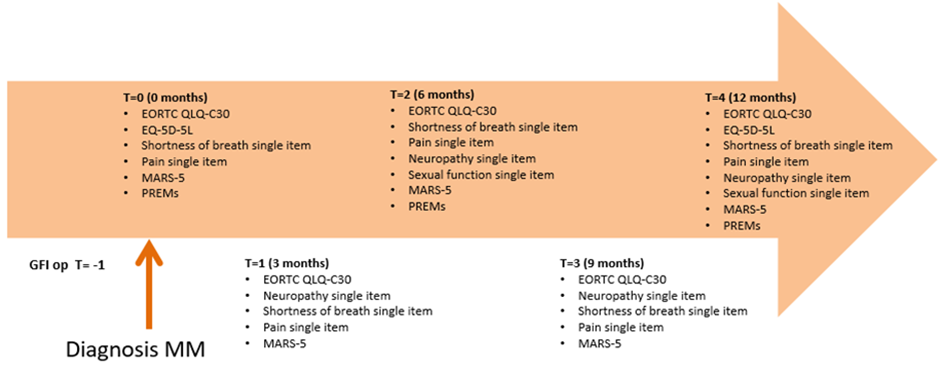


*Figure 6. GFI: Groninger Frailty Index; EQ-5D-5L: The EQ-5D-5L questionnaire has two components: health state description and evaluation. In the description part, health status is measured in terms of five dimensions (5D); mobility, self-care, usual activities, pain/discomfort, and anxiety/depression; EORTC-QLQ-C30: The EORTC QLQ-C30 was developed in 1988 and is one of the most widely used health-related quality of life (HRQOL) questionnaires in cancer research; MARS-5: The Medication Adherence Report Scale is a measurement aimed to develop a questionnaire measure of patients 'adherence to medication’; PREMs: patient reported experiences measurements.*

------------------------------------------------------------------------------------------------------

**t0 is diagnosis (EQ-5D-5L, EORTC-QLQ-C30, SDM-Q-9 and MARS-5)**

**t1 (3 months) follow up (EORTC-QLQ-C30 and MARS-5)**

**t2 (6 months) follow up (EORTC-QLQ-C30, SDM-Q-9 and MARS-5)**

**t3 (9 months) follow up (EORTC-QLQ-C30 and MARS-5)**

**t4 (12 months) follow up (EQ-5D-5L, EORTC-QLQ-C30, SDM-Q-9 and MARS-5)**

**t5 (36 months progression free survival)**

The EQ-5D-5L and SDM-Q-9 are specific questionnaires for this study, The EQ-5D-5L will be used for a CEA and the SDM-Q-9 for a publication around the topic: “Shared Decision Making”. The other questionnaires or single items are potentially care as usual in the future.

------------------------------------------------------------------------------------------------------

## Randomisation, blinding and treatment allocation

- First every recently diagnosed patient (potential subject) will be screened on minimal digital skills, by the research nurse to check if they are able to interact with an e-coach and whether they can read and understand the Dutch language.
- If a patient is admitted to the study (inclusion criteria), he or she will get a randomized sealed envelope at the secretary of the oncology centre with information about the study arm (intervention or control) and FIT or NOT FIT. After this selection the patient will get an e-coach with all supportive modules or a patient (control) will get only questionnaires at the same time as predefined in the e-coach, digital. Patients are asked to install the app on their own device (with the help of the research nurse) and in exceptional cases, there are certified iPads available for patients, that do not have a computer / iPad or smartphone. This study is not double-blind because every participant, health-care professional, and staff member knows in which arm the patient entered.
- Participants, health-care professionals, and staff who assessed outcome measures will not masked to treatment allocation.
- Enrolment in the intervention/care as usual (CAU) group is enriched for a preplannend subgroup with low baseline computer experience as part of a feasibility study (F-ITUMM study). Also the enrolment of patients in two subgroups < 70 years (fit) and > 70 years (not fit) is randomized 1:1 in the intervention group and CAU group.
- Every recently diagnosed multiple myeloma patient qualifies for study participation. Patients older than 70 years and/or not fit patient and patients younger than 70 years and/or fit patient (eligible for HSCT) are distributed equally in the two arms by stratified randomization. Not fit patients and younger than 70 years according to the clinical oncologist.
- A recently diagnosed patient is eligible for a first-line or second-line MM treatment; both groups are distributed equally in the two arms.

# Study procedures

## Plan

**Phase 1. Preparation**

- Introduction at research bureau
- Introduction to healthcare professionals at Isala
- Introduction to hospital pharmacy
- Introduction to oncologists and nurses (haematologists)
- Introduction to patients
- Target population (recently diagnosed MM patients, treatment with oral MM medication)
- Selection of potential study participants (recently diagnosed MM patients, during routine clinical care, Appendix S6)
- Approaching of study participants

Information letter with information on (Appendix S9):

-current practice

-use of the e-coach

-informed consent (Appendix S8 and S9) Subjects will be informed that their participation is voluntary. Subjects will then be required to sign a statement of informed consent that meets the requirements of 21 CFR 50, local regulations, ICH guidelines, Health Insurance Portability and Accountability Act requirements, where applicable, and the IRB/IEC or study site.

The subject must personally sign and date the IRB/IEC informed consent before commencement of study-specific procedures

- **Recruitment and inclusion**

The haematologist will be asked to contact an eligible patient and inform them about this study. When a patients considers participation, he/she will then be provided with an information letter (Appendix S9). Patients will be contact by phone (by nurse) within 1 week from their diagnosis to discuss whether they want to participate.

**Phase 2: Execution phase**

Before a patient can start, a number of essential preparations will be met.

**After start of the diagnosis multiple myeloma**

- e-coach login and start of the first session (t=0)
- information about the disease
- information about the medication
- medication bag for the patient after every visit (all medication for a patient during treatment for 30 days)
- pill count (Appendix S7)

**From diagnosis to 3 months**

- clinical visits and preparation in the e-coach for the clinical visit (patient)
- preparation of the clinical visit from the e-coach (haematologist)
- preparation of the clinical visit from the e-coach (nurse)
- (actively) follow up of signals (red flags) and monitoring
- medication bag for the patient after every visit
- pill count

**At 3 months (t=1)**

- clinical visits and preparation in the e-coach for the clinical visit (patient)
- preparation of the clinical visit from the e-coach (haematologist)
- preparation of the clinical visit from the e-coach (nurse)
- (actively) follow up of signals (red flags) and monitoring
- medication bag for the patient after every visit
- all questionnaires and singe item questions and clinical outcomes are measured
- pill count

**From 3 months to 6 months (t=2),** same as at 3 months, with exclusion of the pill count.

**From 6 months to 9 months (t=3),** same as at 3 months, with exclusion of the pill count.

**From 9 months to 12 months (t=4),** same as at 3 months, with inclusion of the pill count.

**Follow up of PFS 12 months -36 months and OS at 60 months**

**Withdrawal of individual subjects**

Patients can leave the study at any time for any reason if they wish to do so without any consequences. The investigator can decide to withdraw a subject from the study for urgent medical reasons.

## Premature termination of the study

In case the study is ended prematurely, the investigator will notify the accredited METC within 15 days, including the reasons for the premature termination. Within one year after the end of the study, the investigator will submit a final study report with the results of the study, including any publications.

# Data analysis

Data will be recorded on data collection forms and will be entered after validation in a computer system for subsequent statistical analyses (SPSS). The data will be handled confidentially and if possible anonymously. Data will not be transparent at patient level (name, date of birth, interventions) for both the company (Amgen) and the developer of the e-coach (SanaNet). Results are first shared internally with the research group and then discussed /shared with both stakeholders.

## Primary study parameter:

Recently Diagnosed Multiple Myeloma patients (RDMM)

An unpaired t-test (also known as an independent t-test) is a statistical procedure that compares the averages/means of two independent or unrelated groups to determine if there is a significant difference between the two. The dependent variable is normally distributed. The variance of data is the same between groups, meaning that they have the same standard deviation and the independent variables must consist of two independent groups.

A goodness-of-fit test will be performed to analyze if the data are normally distributed. If the data are normally distributed the primary endpoint will be analyzed using the independent T-test. In case of non-normality in the data the nonparametric Mann-Whitney U test or Wilcoxon rank test will be used.

The one-way analysis of variance (ANOVA) will be used to determine whether there are any statistically significant differences between the means of the four groups while adjusting for multiple testing. The Kruskal-Wallis test will be used as a nonparametric alternative for the one-way ANOVA in case of non-normality of the data.

- Minimum difference of 10% in compliance by pill count at 3 months after enrolment (appendix S7) between the intervention group (n=75) vs. control group (n=75) - Improved compliance: number of missed doses at baseline (month 1) versus number of missed doses at month 3.

- From NDMM to 3 months) = 15% attribution rate

- Statistical analysis: unpaired t-test (if the data are normally distributed)

- Overall compliance (%) = (month 1 + 2 + 3) / divided 3)

## Secondary study parameters and explorative other study parameters

We will compare patient characteristics and clinical outcomes (compliance by pill count at 12 months, PDC, MARS-5, Quality of Life etc.) between intervention group and control group.

Normally distributed continuous variables will be analyzed using the independent T-test. In case of non-normality in the data the nonparametric Mann-Whitney U test or Wilcoxon rank test will be used. The one-way analysis of variance (ANOVA) will be used to determine whether there are any statistically significant differences between the means of the four groups while adjusting for multiple testing. The Kruskal-Wallis test will be used as a nonparametric alternative for the one-way ANOVA in case of non-normality of the data.

When there is an overall difference between the groups, we will perform post-hoc analysis to further explore this result, taking into account multiple comparisons.

For all analyses, p-value less than 0.05 will be considered significant.

# ETHICAL CONSIDERATIONS

## Regulation statement

The study will be conducted according to the principles of the Declaration of Helsinki (version 10-2013) in accordance with the Medical Research Involving Human Subjects Act (WMO) and with the ICH-GCP, GMP and GLP guidelines. The protocol, protocol amendments, ICF, and other relevant documents (eg, subject recruitment advertisements) will be submitted to the Institutional ReviewBoard/Independent Ethics Committee (IRB/IEC), Isala METC, by the investigator for review and approval

## Recruitment and consent

Patients usual care involves visiting the cancer centre for diagnosis and outpatient visits to discuss the treatment plan. Timings vary, but this typically happens 3 weeks before starting treatment. After their diagnosis, a clinical member of the planning team will briefly outline the study to patients. Eligible and willing patients will be introduced to the researcher (JFHE) and have the opportunity to discuss the study. If the researcher is not available, then a briefed pre-treatment nurse can give the patient a study pack. Patients can indicate their interest by posting the reply slip. All patients expressing interest in participating in the study, via face-to-face invitation or the reply slip, will be contacted by telephone to discuss the study. All patients will be given a minimum of 24 hours after the initial invitation before being phoned and will have the opportunity to have any questions answered. Study eligibility will be checked. After signing of informed consent and handing in the form to the primary investigator, patients will be included in the study.

## Incentives (if applicable)

Besides the e-coach, patients (subjects) will not receive a reimbursement for participating in this study.

# ADMINISTRATIVE ASPECTS, MONITORING AND PUBLICATION

## Handling and storage of data and documents

Primary outcomes are noted per vised to the pharmacy in the hospital. A special pill count form is developed for the health care providers (Appendix S7). Questionnaires for secondary outcomes asked by Sananet, a company that developes e-coaches (section 6.1) and partner of Isala. Questionnaires for explorative parameters asked by the multiple myeloma SanaCoach. Clinical outcomes are collected from the electronic patient files.

The investigator shall ensure that the subject’s confidentiality is maintained during the study.

Subject will be assigned a unique identifier by XXX. Any subject records or datasets that are transferred to XXX will contain the identifier only; subject names or any information which would make the subject identifiable will not be transferred.

In compliance with governmental regulations/ICH GCP Guidelines, it is required that the investigator and institution permit authorized representatives of the regulatory agency(s), and the IRB/IEC direct access to review the subject’s original medical records for verification of study-related procedures and data. Direct access includes examining, analyzing, verifying, and reproducing any records and reports that are important to the evaluation of the study. The investigator is obligated to inform and obtain the consent of the subject to permit such individuals to have access to his/her study-related records, including personal information.

## Amendments

Amendments are changes made to the research after a favourable opinion by the accredited METC has been given. All amendments will be notified to the METC that gave a favourable opinion.

[A ‘substantial amendment’ is defined as an amendment to the terms of the METC application, or to

the protocol or any other supporting documentation, that is likely to affect to a significant degree:

• The safety or physical or mental integrity of the subjects of the study;

• The scientific value of the study;

• The conduct or management of study; or

• the quality or safety of any intervention used in the study.

[All substantial amendments will be notified to the METC that gave a favourable opinion.]

[Non-substantial amendments will not be notified to the accredited METC, but will be recorded and filed by the sponsor.]

## Safety reporting for Amgen products:

Reporting of adverse events, unanticipated serious adverse device effects and product complaints after awareness of the investigator (sponsor), report within 1 business day to Amgen Safety.

**Unanticipated Serious Adverse Device Effects**, (USADEs), Serious Adverse Device Effects (SADEs) and Non-serious Adverse Device Effect (Non-serious ADEs). Adverse device effect (ADE) is: any adverse effect caused by or associated with the use of a device constituent of a combination product or medical device. Adverse device effects include, but are not limited to, adverse effects resulting from insufficient or inadequate instructions for use, any malfunction of the device, or use error or intentional misuse of the device.

**Product Complaint** is: Any written, electronic or oral communication that alleges deficiencies related to the identity, quality, durability, reliability, safety, effectiveness, or performance of a drug, combination product, or device after it is released for distribution to market or clinic by either: (1) Amgen or (2) distributors or partners for whom Amgen manufactures the material. This includes all components distributed with the drug, such as packaging, drug containers, delivery system, labelling, and inserts. Examples include:

• Device that is damaged or broken

• Bent or blunt needles

• Missing or illegible labeling

• Inability of customer to administer the product

• Product with an unexpected color, appearance, or particles

• Use error (i.e, an act or omission of an act that results in a different combination product or medical device response than intended by the manufacturer or expected by the user, where the user attempted to use the combination product or medical device in good faith and experienced difficulty or deficiency administering the product).

Reports of misuse of a combination product or medical device (i.e, the intentional and improper use of a combination product or medical device not in accordance with the authorized product information) are not considered Product complaints.

**Aggregate reports** (as applicable) listing for Safety data reconciliation are once per year and at the end of the study. Specific requirements are to be outlined in the Research Agreement. Listing for reconciliation should include all ICSRs submitted to Amgen Safety per contract (ADRs, SADRs, Other Safety Findings, USADEs, SADEs and non-serious ADEs)

## Annual progress report

The investigator will submit a summary of the progress of the study to METC Isala once a year. Information will be provided on the date of inclusion of the first subject, numbers of subjects included and numbers of subjects that have completed the trial, serious adverse events/ serious adverse reactions, othersafety findings (for example lactation and/or pregnancy problems) , and amendments.

Notifying the IRB/IEC of serious adverse events occurring at the site, deviations from the protocol or other adverse event reports, in accordance with local procedures. Every healthcare provider who notices a treatment / medication-related averse event, will report this to the Lareb according to Dutch guidelines. All adverse events are registered in the study by means of measurements in the e-coach or in the electronic patient file.

## Temporary halt and (prematurely) end of study report

The investigator/sponsor will notify METC Isala and Amgen of the end of the study within a period of 8 weeks. The end of the study is defined as the last (subject) patients last visit.

- The sponsor will notify the METC and Amgen immediately of a temporary halt of the study, including the reason of such an action.
- In case the study is ended prematurely, the sponsor will notify the accredited METC and Amgen within 15 days, including the reasons for the premature termination.
  Within one year after the end of the study, the investigator/sponsor will submit a final study report with the results of the study, including any publications/abstracts of the study, to the accredited METC and Amgen.

## Public disclosure and publication policy

This study investigational plan, documentation, data and all other information generated will be held in strict confidence by the investigator and their representatives. No information concerning the study or the data will be released to any unauthorized third party without prior written approval by the Steering Committee. All results obtained by this study will be disclosed unreservedly according the basic principles of the CCMO [24]. We will register this clinical study in a public trial register according the requirement of the International Committee of Medical Journal Editors[25].

# APPENDICES

## S1 F-ITUMM Study (abstract)


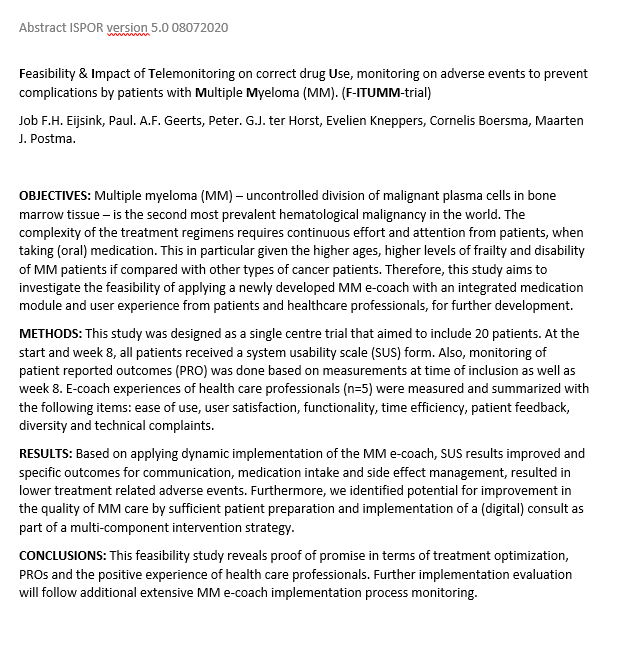


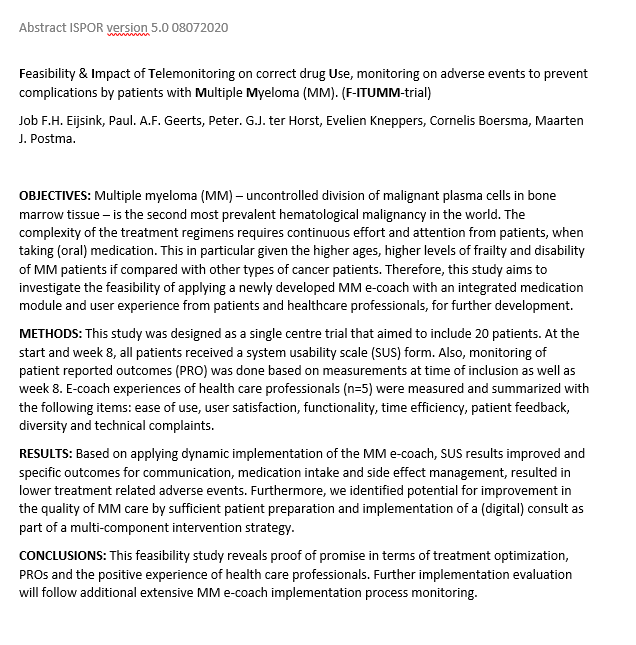


## S2 Medication

| nummer | Kuur |
| --- | --- |
| 1 | Dara-vmp |
| 2 | Daratumumab VTD |
| 3 | VMP (>75 jaar) |
| 4 | VMP (18 tm 64 jaar) |
| 5 | VMP (65 tm 74 jaar) |
| 6 | len/dex |
| 7 | VD |
| 8 | VRD |
| 9 | VTD |
| 10 | Dara-len-dex |
| 11 | Lenalidomide onderhoud |
| 12 | KRd |
| 13 | Erd |
| 14 | Ird |
| 15 | REP |
| 16 | Kd (Car-dex) |
| 17 | Epd |
| 18 | Pomalidomide +cyclofosfamide ("PEP") |
| 19 | Pomalidomide +carfilzomib, dexamethason |
| 20 | Cyclofosfamide mobilisatie |

| **Kuur** | **Stofnaam** | **Meestvoorkomende merknamen** | **dosering: sterkte+eenheid  (bij alle medicijnen nodig, ook al is het niet exacte dosering (bv 16mg/kg)** | **toedien vorm** | **inname informatie** | **Door patiënt zelf in te nemen (ja, nee)?** | | **Innametijdstippen (tijdstip(pen), anders)** | **Zo Nodig** |
| --- | --- | --- | --- | --- | --- | --- | --- | --- | --- |
| Dara-vmp | *Daratumumab* |  | *16mg/kg* | *via het infuus* | *Cyclus 1: Dag 1,8,15,22,29,36 Cyclus 2 t/m 9: Dag 1,22 Vervolg cycli; dag 1 (a 4 weken)* | | *nee* |  |  |
|  | *Bortezomib* | *Velcade* | *1,3mg/m2* | *via een injectie* | *Cyclus 1:1,4,8,11,22,25,29,32 Cyclus 2 t/m 9: 1,8,22,29 vervolgcycli stop* | | *nee* |  |  |
|  | *Melfalan* | *Alkeran, Melfalan* | *9mg/m2* | *oraal* | *Dag 1 tot en met dag 4 cyclus 1 tot en met 9. vervolgecycli stop* | | *ja* | *8 uur* |  |
|  | *Prednisolon* | *Prednison* | *60mg/m2* | *oraal* | *Dag 2 tot en met 4, bij het ontbijt innemen kuur 1 tm 9. kuur 10 stop* | | *ja* | *8.00 uur* |  |
|  | *Paracetamol* |  | *1000 mg* | *oraal* | *1 uur voor het infuus 2 tabletten van 500 mg Cyclus 1: Dag 1,8,15,22,29,36 Cyclus 2 t/m 9: Dag 1,22 Vervolg cycli; dag 1 (a 4 weken)* | | *ja* | *1 uur voor de kuur* |  |
|  | *Prednisolon* | *Prednison* | *100 mg* | *via het infuus* | *Cyclus 1: Dag 1,8,15,22,29,36 Cyclus 2 t/m 9: Dag 1,22 Vervolg cycli; dag 1 (a 4 weken)* | | *nee* |  |  |
|  | *Clemastine* | *Tavegyl* | *2 mg* | *via het infuus* | *1 uur voor het infuus Cyclus 1: Dag 1,8,15,22,29,36 Cyclus 2 t/m 9: Dag 1,22 Vervolg cycli; dag 1 (a 4 weken)* | | *nee* |  |  |
|  | *Valaciclovir* | *Zelitrex* | *500 mg* | *oraal* | *2 keer per dag, continu* | | *ja* | *8 uur en 18 uur* |  |
|  | *Co-trimoxazol* |  | *480 mg* | *oraal* | *1 keer per dag, continu* | | *ja* | *8 uur* |  |
|  | *Prednisolon* |  | *20 mg* | *oraal* | *cyclus 1: dag 9,10,16,17,23,24,30,31,37,38 cyclus 2 t/m9 dag 23+24* | | *ja* | *8 uur* |  |
|  | *Desloratadine* | *Aerius* | *5 mg* | *oraal* | *1x per dag cyclus 1 dag 2,3,9,10,16,17,23,24,30,31,37,38 cyclus 2-9 dag 2,3,23,23 bij kuur 9 ev dag 2 en 3?* | | *ja* | *8 uur* |  |
|  | *Salmeterol/Fluticason* | *Seretide* | *25/125 microgr* | *inhalatie* | *Puffje; 2x per dag 1;* ***zonodig*** *bij longproblemen* | | *ja* | *8 uur en 18 uur* | *X* |
|  | *Ondansetron* | *Zofran* | *8 mg* | *oraal* | *2x daags, op dag 1 tm 4 van de cyclus* | | *ja* | *1 uur voor de kuur en 18 uur op dag 1 van de kuur* |  |
|  | *Metoclopramide* | *primperan* | *10 mg* | *oraal/zetpil* | *zo nodig bij misselijkheid* | | *ja* | *zo nodig, 3x per dag maximaal (start bij misselijkheid, 8 uur later een volgende tablet of zetpil)* | *X* |
| Kuur | *Stofnaam* | *Meestvorkomende merknamen* | |  | *inname informatie* | |  |  |  |
| Daratumumab VTD | *Daratumumab* |  | *16 mg/kg* | *via het infuus* | *Cyclus 1: Dag 1,8,15,22,29,36 Cyclus 2 t/m 9: Dag 1,22 Vervolg cycli; dag 1 (a 4 weken)* | | *nee* |  |  |
|  | *Bortezomib* | *Velcade* | *1,3mg/kg* | *via een injectie* | *dag 1,4,8,11* | | *nee* |  |  |
| geen ipova document | *Thalidomide* | *Celgene* | *100 mg* | *oraal* | *Dagelijks innemen voor de nacht* | | *ja* | *22 uur* |  |
|  | *Dexamethason* |  | *zie F* | *oraal* | *kuur 1 en 2 40 mg. 1,2,8,9,15 en 16. Kuur 3 en 4 40 mg dag 1,2 en 20 mg dag 8,9,15 en 16 dag. Innemen bij het ontbijt* | | *ja* | *8 uur* |  |
|  | *Valaciclovir* | *Zelitrex* | *500 mg* | *oraal* | *2x per dag continu* | | *ja* | *8 uur en 18 uur* |  |
|  | *Co-trimoxazol* |  | *480 mg* | *oraal* | *1 x per dag continu* | | *ja* | *8 uur* |  |
|  | *Fluconazol* | *Diflucan* | *50 mg* | *oraal* | *1 x per dag continu* | | *ja* | *8 uur* |  |
|  | *Nadroparine* | *Fraxiparine* | *0,3 ml* | *onderhuidse injectie* | *1 x per dag; luchtbel niet verwijderen.* | | *ja, of thuiszorg* | *vast tijdstip op de dag* |  |
|  | *Metoclopramide* | *primperan* | *10 mg* | *oraal/zetpil* | *zo nodig bij misselijkheid* | | *ja* | *zo nodig, 3x per dag maximaal (start bij misselijkheid, 8 uur later een volgende tablet of zetpil)* | *X* |
|  |  |  |  |  |  | |  |  |  |
| Kuur | *Stofnaam* | *Meestvorkomende merknamen* | |  | *inname informatie* | |  |  |  |
| VMP |  |  |  |  |  | |  |  |  |
| >75 jaar | *Bortezomib* | *Velcade* | *1,3 mg/m2* | *via het infuus* | *dag 1, 8, 15, 22* | | *nee* |  |  |
|  | *Melfalan* | *Alkeran, Melfalan* | *9mg/m2* | *oraal* | *Dag 1 tot en met dag 4* | | *ja* | *8 uur* |  |
|  | *Prednisolon* | *Prednison* | *1 mg/kg* | *oraal* | *Dag 1 tot en met 4* | | *ja* | *8 uur* |  |
|  | *Valaciclovir* | *Zelitrex* | *500 mg* | *oraal* | *2x per dag continu* | | *ja* | *8 en 18 uur* |  |
|  | *Co-trimoxazol* |  | *480 mg* | *oraal* | *1 x per dag continu* | | *ja* | *8 uur* |  |
|  | *Ondansetron* | *Zofran* | *8 mg* | *oraal* | *dag 1 tot en met 4: 2x daags* | | *ja* | *7 uur (1 uur voor Melfalan) en 18 uur* |  |
|  | *Metoclopramide* | *Primperan* | *10 mg* | *oraal/zetpil* | *zo nodig bij misselijkheid* | | *ja* | *zo nodig, 3x per dag maximaal (start bij misselijkheid, 8 uur later een volgende tablet of zetpil)* | *X* |
|  |  |  |  |  |  | |  |  |  |
| VMP |  |  |  |  |  | |  |  |  |
| 18 tm 64 jaar | *Bortezomib* | *Velcade* | *1,3 mg/m2* | *via een injectie* | *cyclus 1 tot en met 4:dag 1, 4, 8, 11, 22,25, 29, 32 cyclus 5 en verder dag 1, 8, 22, 29* | | *nee* |  |  |
|  | *Melfalan* | *Alkeran, Melfalan* | *9 mg/m2* | *oraal* | *Dag 1 tot en met dag 4* | | *ja* | *dag 1 bij start kuur, dag 2,3,4 bij ontbijt* |  |
|  | *Prednisolon* | *Prednison* | *2 mg/kg* | *oraal* | *Dag 1 tot en met 4, bij het ontbijt innemen* | | *ja* | *8 uur* |  |
|  | *Valaciclovir* | *Zelitrex* | *500 mg* | *oraal* | *2x per dag continu* | | *ja* | *8 en 18 uur* |  |
|  | *Co-trimoxazol* |  | *480 mg* | *oraal* | *1 x per dag continu* | | *ja* | *8 uur* |  |
|  | *Zoledroninezuur* | *Zometa* | *4 mg* | *via het infuus* | *1x per 4 weken via het infuus* | | *nee* |  |  |
|  | *Ondansetron* | *Zofran* | *8 mg* | *oraal* | *dag1 tot en met 4 2x daags* | | *ja* | *8 en 18 uur* |  |
|  | *Metoclopramide* | *Primperan* | *10 mg* | *oraal/zetpil* | *zo nodig bij misselijkheid* | | *ja* | *zo nodig, 3x per dag maximaal (start bij misselijkheid, 8 uur later een volgende tablet of zetpil)* | *X* |
|  |  |  |  |  |  | |  |  |  |
| VMP | *Bortezomib* | *Velcade* | *1,3 mg/m2* | *via een injectie* | *cyclus 1 tot en met 4:dag 1, 4, 8, 11, 22,25, 29, 32 cyclus 5 en verder dag 1, 8, 22, 29* | | *nee* |  |  |
| 65 tm 74 jaar | *Melfalan* | *Alkeran, Melfalan* | *9 mg/m2* | *oraal* | *Dag 1 tot en met dag 4* | | *ja* | *dag 1 bij start kuur, dag 2,3,4 bij ontbijt* |  |
|  | *Prednisolon* | *Prednison* | *1 mg/kg* | *oraal* | *Dag 1 tot en met 4, bij het ontbijt innemen* | | *ja* | *8 uur* |  |
|  | *Valaciclovir* | *Zelitrex* | *500 mg* | *oraal* | *2x per dag continu* | | *ja* | *8 en 18 uur* |  |
|  | *Co-trimoxazol* |  | *480 mg* | *oraal* | *1 x per dag continu* | | *ja* | *8 uur* |  |
|  | *Zoledroninezuur* | *Zometa* | *4 mg* | *via het infuus* | *1x per 4 weken via het infuus* | | *nee* |  |  |
|  | *Ondansetron* | *Zofran* | *8 mg* | *oraal* | *dag1 tot en met 4 2x daags* | | *ja* | *8 en 18 uur* |  |
|  | *Metoclopramide* | *Primperan* | *10 mg* | *oraal/zetpil* | *zo nodig bij misselijkheid* | | *ja* | *zo nodig, 3x per dag maximaal (start bij misselijkheid, 8 uur later een volgende tablet of zetpil)* | *X* |
|  |  |  |  |  |  | |  |  |  |
| Kuur | *Stofnaam* | *Meestvorkomende merknamen* | |  | *inname informatie* | |  |  |  |
| len/dex |  |  |  |  |  | |  |  |  |
|  | *Lenalidomide* | *Revlimid* | *25 mg* | *oraal* | *dag 1 tot en met 21; voor de nacht innemen* | | *ja* | *22 uur* |  |
|  | *Dexamethason* |  | *40 mg* | *oraal* | *innemen bij het ontbijt, dag 1,8,15 en 22* | | *ja* | *8 uur* |  |
|  | *Nadroparine* | *Fraxiparine* | *0.3 ml* | *onderhuidse injectie* | *1 x per dag; luchtbel niet verwijderen. Continu* | | *ja, of thuiszorg* | *vast tijdstip op de dag* |  |
|  | *Co-trimoxazol* |  | *480 mg* | *oraal* | *1 keer per dag, continu* | | *ja* | *8 uur* |  |
| Kuur | *Stofnaam* | *Meestvorkomende merknamen* | |  | *inname informatie* | |  |  |  |
| VD | *Bortezomib* | *Velcade* | *1,3 mg/m2* | *via een injectie* | *dag 1, 4, 8 en 11* | | *nee* |  |  |
|  | *Dexamethason* |  | *40 mg* | *oraal* | *innemen bij het ontbijt, dag 1,8 en 15* | | *ja* | *8 uur* |  |
|  | *Omeprazol* |  | *40 mg* | *oraal* | *1x daags, continu* | | *ja* | *8 uur* |  |
|  | *Acetylsalicylzuur* |  | *80 mg* | *oraal* | *1x daags, continu* | | *ja* | *8 uur* |  |
|  | *Fluconazol* | *Diflucan* | *50 mg* | *oraal* | *1 x per dag continu* | | *ja* | *8 uur* |  |
|  | *Co-trimoxazol* |  | *480 mg* | *oraal* | *1 keer per dag, continu* | | *ja* | *8 uur* |  |
|  | *Valaciclovir* | *Zelitrex* | *500 mg* | *oraal* | *2x per dag continu* | | *ja* | *8 en 18 uur* |  |
|  | *Metoclopramide* | *Primperan* | *10 mg* | *oraal/zetpil* | *zo nodig bij misselijkheid* | | *ja* | *zo nodig, 3x per dag maximaal (start bij misselijkheid, 8 uur later een volgende tablet of zetpil)* | *X* |
| Kuur | *Stofnaam* | *Meestvorkomende merknamen* | |  | *inname informatie* | |  |  |  |
| VRD | *Bortezomib* | *Velcade* | *1,3 mg/m2* | *via een injectie* | *dag 1, 4, 8 en 11* | | *nee* |  |  |
|  | *Lenalidomide* | *Revlimid* | *25 mg* | *oraal* | *dag 1 tot en met 21 voor de nacht innemen* | | *ja* | *22 uur* |  |
|  | *Dexamethason* |  | *20 mg* | *oraal* | *innemen bij het ontbijt, dag 1,2,4,5,8,9,11 en 12* | | *ja* | *8 uur* |  |
|  | *Omeprazol* |  | *40 mg* | *oraal* | *1x daags, continu* | | *ja* | *8 uur* |  |
|  | *Nadroparine* | *Fraxiparine* | *0.3 ml* | *onderhuidse injectie* | *1 x per dag; luchtbel niet verwijderen. Continu* | | *ja, of thuiszorg* | *vast tijdstip op de dag* |  |
|  | *Acetylsalicylzuur* |  | *80 mg* | *oraal* | *1x daags, continu* | | *ja* | *8 uur* |  |
|  | *Fluconazol* | *Diflucan* | *50 mg* | *oraal* | *1 x per dag continu* | | *ja* | *8 uur* |  |
|  | *Co-trimoxazol* |  | *480 mg* | *oraal* | *1 keer per dag, continu* | | *ja* | *8 uur* |  |
|  | *Valaciclovir* | *Zelitrex* | *500 mg* | *oraal* | *2x per dag continu* | | *ja* | *8 en 18 uur* |  |
|  | *Metoclopramide* | *Primperan* | *10 mg* | *oraal/zetpil* | *zo nodig bij misselijkheid* | | *ja* | *zo nodig, 3x per dag maximaal (start bij misselijkheid, 8 uur later een volgende tablet of zetpil)* | *X* |
| Kuur | *Stofnaam* | *Meestvorkomende merknamen* | |  | *inname informatie* | |  |  |  |
|  | *Bortezomib* | *Velcade* | *1,3 mg/m2* | *via een injectie* | *dag 1, 4, 8 en 11* | | *nee* |  |  |
| VTD | *Thalidomide* | *Celgene* | *100 mg* | *oraal* | *Dagelijks innemen voor de nacht* | | *ja* | *22 uur* |  |
|  | *Dexamethason* |  | *zie F* | *oraal* | *kuur 1 en 2 40 mg. 1,2,8,9,15 en 16.Kuur 3 en 4 40 mg dag 1,2 en 20 mg dag 8,9,15 en 16 dag. Innemen bij het ontbijt* | | *ja* | *8 uur* |  |
|  | *Valaciclovir* | *Zelitrex* | *500 mg* | *oraal* | *2x per dag continu* | | *ja* | *8 en 18 uur* |  |
|  | *Co-trimoxazol* |  | *480 mg* | *oraal* | *1 keer per dag, continu* | | *ja* | *8 uur* |  |
|  | *Fluconazol* | *Diflucan* | *50 mg* | *oraal* | *1 x per dag continu* | | *ja* | *8 uur* |  |
|  | *Nadroparine* | *Fraxiparine* | *0.3ml* | *onderhuidse injectie* | *1 x per dag; luchtbel niet verwijderen.* | | *ja of thuiszorg* | *vast tijdstip op de dag* |  |
|  | *Metoclopramide* | *Primperan* | *10 mg* | *oraal/zetpil* | *zo nodig bij misselijkheid* | | *ja* | *zo nodig, 3x per dag maximaal (start bij misselijkheid, 8 uur later een volgende tablet of zetpil)* | *X* |
|  |  |  |  |  |  | |  |  |  |
| Kuur | *Stofnaam* | *Meestvorkomende merknamen* | |  | *inname informatie* | |  |  |  |
| Dara-len-dex | *Daratumumab* |  | *16 mg/kg* | *via het infuus* | *Cyclus 1 & 2 : Dag 1,8,15,22 Cyclus 3 t/m 6: Dag 1,15 Vervolg cycli : Dag 1* | | *nee* |  |  |
|  | *Lenalidomide* | *Revlimid* | *25 mg* | *oraal* | *dag 1 tot en met 21; voor de nacht innemen* | | *ja* | *22 uur* |  |
|  | *Dexamethason* |  | *zie F* | *oraal* | *kuur 1 en 2 dag 1,2,8,9,15,16,22 en 23 20 mg. Kuur 3 tot 6 dag 1,2,15 en 16 20mg dag 8 en 22 40mg vervolg cycli dag 1,2 20 mg dag 8,15,22 40mg innemen bij het ontbijt* | | *ja* | *8 uur* |  |
|  | *Paracetamol* |  | *1000 mg* | *oraal* | *1 uur voor het infuus 2 tabletten van 500 mg* | | *ja* | *1 uur voor de kuur* |  |
|  | *Clemastine* | *Tavegyl* | *2 mg* | *intraveneus* | *1 uur voor het infuus* | | *nee* |  |  |
|  | *Nadroparine* | *Fraxiparine* | *0.3 ml* | *onderhuidse injectie* | *1 x per dag; luchtbel niet verwijderen. Continu* | | *ja of thuiszorg* | *vast tijdstip op de dag* |  |
|  | *Valaciclovir* | *Zelitrex* | *500 mg* | *oraal* | *2x per dag continu* | | *ja* | *8 uur en 18 uur* |  |
|  | *Co-trimoxazol* |  | *480 mg* | *oraal* | *1 keer per dag, continu* | | *ja* | *8 uur* |  |
|  | *Fluconazol* | *Diflucan* | *50 mg* | *oraal* | *1 x per dag continu* | | *ja* | *8 uur* |  |
|  | *Omeprazol* |  | *20 mg* | *oraal* | *1x daags, continu* | | *ja* | *8 uur* |  |
|  | *Desloratadine* |  | *5 mg* | *oraal* | *zo nodig kuur 1 dag 1,2,8,9,15,16, 22 en 23. kuur 2 dag 1,2 en 15 en 16* | | *ja* | *8 uur* | *X* |
|  | *Salmeterol/Fluticason* | *Seretide* | *25/125 microgr* | *inhalatie* | *Puffje; 2x per dag 1; zonodig bij longproblemen* | | *ja* | *8 en 18 uur* | *X* |
| Kuur | *Stofnaam* | *Meestvorkomende merknamen* | |  | *inname informatie* | |  |  |  |
| Lenalidomide onderhoud | |  |  |  |  | |  |  |  |
|  | *Lenalidomide* | *Revlimid* | *10 mg* | *oraal* | *dag 1 tot en met 21 10 mg; voor de nacht innemen* | | *ja* | *22 uur* |  |
|  | *Nadroparine* | *Fraxiparine* | *0.3 ml* | *onderhuidse injectie* | *1 x per dag; luchtbel niet verwijderen.* | | *ja of thuiszorg* | *vast tijdstip op de dag* |  |
|  |  |  |  |  |  | |  |  |  |
|  |  |  |  |  |  | |  |  |  |
| Kuur | *Stofnaam* | *Meestvorkomende merknamen* | |  | *inname informatie* | |  |  |  |
| KRd | *Carfilzomib* |  | *Kuur 1 dag 1 en 2 20 mg/m2 (max 44mg) dag 8,9,15 en 16 27mg/m2 (max 60mg) kuur 2 en volgende 27mg/m2 (max 60mg)* | *via het infuus* | *dag 1,2,8,9,15 en 16* | | *nee* |  |  |
|  | *Lenalidomide* | *Revlimid* | *25 mg* | *oraal* | *dag 1 tot en met 21; voor de nacht innemen* | | *ja* | *22 uur* |  |
|  | *Dexamethason* |  | *20 mg* | *oraal* | *innemen bij het ontbijt; dag 1,2,8,9,15,16 en 22* | | *ja* | *8 uur* |  |
|  |  |  |  |  |  | |  |  |  |
|  | *Valaciclovir* | *Zelitrex* | *500 mg* | *oraal* | *2x per dag continu* | | *ja* | *8 en 22 uur* |  |
|  | *Ciprofloxacin* |  | *500 mg* | *oraal* | *2x per dag continu* | | *ja* | *8 en 22 uur* |  |
|  | *Fluconazol* | *Diflucan* | *50 mg* | *oraal* | *1 x per dag continu* | | *ja* | *8 uur* |  |
|  | *Co-trimoxazol* |  | *480 mg* | *oraal* | *1 keer per dag, continu* | | *ja* | *8 uur* |  |
|  |  |  |  |  |  | |  |  |  |
|  | *Allopurinol* |  | *200 mg* | *oraal* | *zo nodig 1x daags* | | *ja* | *8 uur* | *X* |
|  | *Rasburicase* |  | *3mg* | *via het infuus* | *zo nodig via infuus* | | *nee* | *30 min voor start van chemotherapie of prednisolon* | *X* |
|  | *Metoclopramide* | *Primperan* | *10 mg* | *oraal/zetpil* | *zo nodig bij misselijkheid* | | *ja* | *zo nodig, 3x per dag maximaal (start bij misselijkheid, 8 uur later een volgende tablet of zetpil)* | *X* |
| Kuur | *Stofnaam* | *Meestvorkomende merknamen* | |  | *inname informatie* | |  |  |  |
| Erd | *Elotuzumab* |  | *10 mg/kg* | *via het infuus* | *kuur 1 en 2 dag 1,8,15,22; kuur 3 en verder dag 1 en 15* | | *nee* |  |  |
|  | *Lenalidomide* | *Revlimid* | *25 mg* | *oraal* | *dag 1 tot en met 21; voor de nacht innemen* | | *ja* | *22 uur* |  |
|  | *Dexamethason* |  | *28 mg* | *oraal* | *kuur 1 en 2: 28 mg dag 1,8,15,22 kuur 3 en verder: dag 1 en 15 28mg en dag 8 en 22 40mg* | | *ja* | *8 uur* |  |
|  | *Dexamethason* |  | *8 mg* | *via het infuus* | *via het infuus* | | *nee* |  |  |
|  | *Clemastine* | *Tavegyl* | *2 mg* | *via het infuus* | *Via het infuus* | | *nee* |  |  |
|  | *Ranitidine* |  | *50 mg* | *via het infuus* | *via het infuus* | | *nee* |  |  |
|  | *paracetamol* |  | *1000 mg* | *oraal* | *45-90 min voor infuus 2 tabletten van 500 mg* | | *ja* | *45-90 min voor het infuus* |  |
|  | *Nadroparine* | *Fraxiparine* | *0.3 ml* | *onderhuidse injectie* | *1 x per dag; luchtbel niet verwijderen. Continu* | | *ja of thuiszorg* | *vast tijdstip op de dag* |  |
|  | *Co-trimoxazol* |  | *480 mg* | *oraal* | *1 keer per dag, continu* | | *ja* | *8 uur* |  |
|  |  |  |  |  |  | |  |  |  |
| Kuur | *Stofnaam* | *Meestvorkomende merknamen* | |  | *inname informatie* | |  |  |  |
| Ird | *Ixazomib* |  | *volgens voorschrift* |  | *dag 1,8,15* | | *ja* | *?* |  |
|  | *Lenalidomide* | *Revlimid* | *volgens voorschrift* | *oraal* | *dag 1 tot 28* | | *ja* | *22 uur* |  |
|  | *Dexamethason* |  | *volgens voorschrift* | *oraal* | *dag 1,8,15,22* | | *ja* | *8 uur* |  |
|  |  |  |  |  |  | |  |  |  |
| Kuur | *Stofnaam* | *Meestvorkomende merknamen* | |  | *inname informatie* | |  |  |  |
| REP | *Lenalidomide* | *Revlimid* | *10 mg* | *oraal* | *dag 1 tot en met 21 15mg; voor de nacht innemen* | | *ja* | *22 uur* |  |
|  | *Cyclofosfamide* | *Endoxan* | *50-100 mg afhankelijk van beenmerg reserve (REP 50 en de REP100 kuur aanmaken)* | *oraal* | *1x per dag, continu 'S ochtend innemen.* | | *ja* | *8 uur* |  |
|  | *Prednisolon* |  | *Kuur 1 en 2 20 mg, overige kuren 10 mg* | *oraal* | *1x per dag, continu 'S ochtend innemen* | | *ja* | *8 uur* |  |
|  | *Omeprazol* |  | *40 mg* | *oraal* | *1x daags, continu* | | *ja* | *8 uur* |  |
|  | *Acetylsalicylzuur* |  | *80 mg* | *oraal* | *1x daags, continu* | | *ja* | *8 uur* |  |
|  | *Co-trimoxazol* |  | *480 mg* | *oraal* | *1 keer per dag, continu* | | *ja* | *8 uur* |  |
|  | *Fluconazol* | *Diflucan* | *50 mg* | *oraal* | *1 x per dag continu* | | *ja* | *8 uur* |  |
|  | *Valaciclovir* | *Zelitrex* | *500 mg* | *oraal* | *2x per dag continu* | | *ja* | *8 en 18 uur* |  |
|  | *Metoclopramide* | *Primperan* | *10 mg* | *oraal/zetpil* | *zo nodig bij misselijkheid* | | *ja* | *zo nodig, 3x per dag maximaal (start bij misselijkheid, 8 uur later een volgende tablet of zetpil)* | *X* |
|  |  |  |  |  |  | |  |  |  |
| Kuur | *Stofnaam* | *Meestvorkomende merknamen* | |  | *inname informatie* | |  |  |  |
| Kd (Car-dex) | *Carfilzomib* |  | *Kuur 1 dag 1 en 2 20 mg/m2 (max 44mg) dag 8,9,15 en 16 56mg/m2 (max 123,2mg) kuur 2 en volgende 56 mg/m2 (max 123,2mg)* | *via het infuus* | *Infuus; dag 1,2,8,9,15 en 16* | | *nee* |  |  |
|  | *Dexamethason* |  | *20 mg* | *oraal* | *Dag 1, 2, 8, 9, 15, 16, bij het ontbijt innemen* | | *ja* | *8 uur* |  |
|  | *Ciprofloxacin* |  | *500 mg* | *oraal* | *2x per dag continu* | | *ja* | *8 en18 uur* |  |
|  | *Fluconazol* | *Diflucan* | *50 mg* | *oraal* | *1 x per dag continu* | | *ja* | *8 uur* |  |
|  | *Valaciclovir* | *Zelitrex* | *500 mg* | *oraal* | *2x per dag continu* | | *ja* | *8 en 18 uur* |  |
|  | *Co-trimoxazol* |  | *480 mg* | *oraal* | *1 keer per dag, continu* | | *ja* | *8 uur* |  |
|  | *Metoclopramide* | *Primperan* | *10 mg* | *oraal/zetpil* | *zo nodig bij misselijkheid* | | *ja* | *zo nodig, 3x per dag maximaal (start bij misselijkheid, 8 uur later een volgende tablet of zetpil)* | *X* |
|  |  |  |  |  |  | |  |  |  |
|  |  |  |  |  |  | |  |  |  |
|  |  |  |  |  |  | |  |  |  |
| Kuur | *Stofnaam* | *Meestvorkomende merknamen* | |  | *inname informatie* | |  |  |  |
| Epd < 75 jaar | *Pomalidomide* | *Imnovid* | *4 mg* | *oraal* | *dag 1 tot en met 21* | | *ja* | *22 uur* |  |
| Epd > 75 jaar | *Dexamethason* |  | *1x daags 40 mg (boven de 75 jaar 20 mg)* | *oraal* | *dag 1,8,15,22 innemen bij ontbijt* | | *ja* | *8 uur* |  |
|  | *Acetylsalicylzuur* |  | *80 mg* | *oraal* | *1x daags, continu* | | *ja* | *8 uur* |  |
|  | *Co-trimoxazol* |  | *480 mg* | *oraal* | *1 keer per dag, continu* | | *ja* | *8 uur* |  |
|  | *Metoclopramide* | *Primperan* | *10 mg* | *oraal/zetpil* | *zo nodig bij misselijkheid* | | *ja* | *zo nodig, 3x per dag maximaal (start bij misselijkheid, 8 uur later een volgende tablet of zetpil)* | *X* |
|  |  |  |  |  |  | |  |  |  |
| Kuur | *Stofnaam* | *Meestvorkomende merknamen* | |  | *inname informatie* | |  |  |  |
| Pomalidomide +cyclofosfamide ("PEP") | *Pomalidomide* | *Imnovid* | *4 mg* | *oraal* | *1 x per dag; dag 1 tm 21* | | *ja* | *22 uur* |  |
| geen iprova document | *Cyclofosfamide* | *Endoxan* |  | *oraal* | *1x per dag, continu. 'S ochtend innemen* | | *ja* | *8 uur* |  |
|  | *Prednisolon* |  | *volgens voorschrift?* | *oraal* | *1x per dag, continu. 'S ochtend innemen* | | *ja* | *8 uur* |  |
|  | *Omeprazol* |  | *40 mg* | *oraal* | *1x daags, continu* | | *ja* | *8 uur* |  |
|  | *Acetylsalicylzuur* |  | *80 mg* | *oraal* | *1x daags, continu* | | *ja* | *8 uur* |  |
|  | *Co-trimoxazol* |  | *480 mg* | *oraal* | *1 keer per dag, continu* | | *ja* | *8 uur* |  |
|  | *Fluconazol* | *Diflucan* | *50 mg* | *oraal* | *1 x per dag continu* | | *ja* | *8 uur* |  |
|  | *Valaciclovir* | *Zelitrex* | *500 mg* | *oraal* | *2x per dag continu* | | *ja* | *8 en 18 uur* |  |
|  | *Metoclopramide* | *Primperan* | *10 mg* | *oraal/zetpil* | *zo nodig bij misselijkheid* | | *ja* | *zo nodig, 3x per dag maximaal (start bij misselijkheid, 8 uur later een volgende tablet of zetpil)* | *X* |
|  |  |  |  |  |  | |  |  |  |
| Kuur | *Stofnaam* | *Meestvorkomende merknamen* | |  | *inname informatie* | |  |  |  |
| Pomalidomide +carfilzomib, dexamethason | *Pomalidomide* | *Imnovid* | *4 mg* | *oraal* | *1 x per dag; dag 1 tm 21* | | *ja* | *22 uur* |  |
|  | *Carfilzomib* |  | *zie F* | *via het infuus* | *Kuur1 : 20 mg/m2 dag dag 1 en 2 36mg/m2 dag 8,9,15 en 16 overige kuren 36mg/m2 dag 1,2,8,9,15,en16* | | *nee* |  |  |
|  | *Dexamethason* |  | *20 mg* | *oraal* | *Dag 1, 2, 8, 9, 15, 16, bij het ontbijt innemen* | | *ja* | *8 uur* |  |
|  | *Nadroparine* | *Fraxiparine* | *0.3 ml* | *onderhuidse injectie* | *1 x per dag; luchtbel niet verwijderen. Continu* | | *ja of thuiszorg* | *vast tijdstip op de dag* |  |
|  | *Ciprofloxacin* |  | *500 mg* | *oraal* | *2x per dag continu* | | *ja* | *8 en 18 uur* |  |
|  | *Valaciclovir* | *Zelitrex* | *500 mg* | *oraal* | *2x per dag continu* | | *ja* | *8 en 18 uur* |  |
|  | *Fluconazol* | *Diflucan* | *50 mg* | *oraal* | *1 x per dag continu* | | *ja* | *8 uur* |  |
|  | *Metoclopramide* | *Primperan* | *10 mg* | *oraal/zetpil* | *zo nodig bij misselijkheid* | | *ja* | *zo nodig, 3x per dag maximaal (start bij misselijkheid, 8 uur later een volgende tablet of zetpil)* | *X* |
|  |  |  |  |  |  | |  |  |  |
| Kuur | *Stofnaam* | *Meestvorkomende merknamen* | |  | *inname informatie* | |  |  |  |
| Cyclofosfamide mobilisatie | *Cyclofosfamide* | *Endoxan* | *1000 mg/m2* | *via het infuus* | *dag 1, via het infuus* | | *nee* |  |  |
|  | *Neupogen* | *Filgrastim* | *volgens voorschrift 2x daags 5 Ug/kg/dag* | *onderhuidse injectie* | *dag 7,8,9,10,11 8 uur en 20 uur. Dag 12 6.30 evt ook 20 uur en dag 13 6.30 evt ook dag 14 6.30 injectie subcutaan* | | *ja of thuiszorg* | *8 uur en 20 uur (op de dag van stamcelafname 6.30 uur)* |  |
|  | *Ondansetron* |  | *8 mg* | *oraal* | *dag 1, 2x per dag. 1 uur voor infuus en bij avondeten* | | *ja* | *1 uur voor de kuur en 18 uur* |  |
|  | *Metoclopramide* | *Primperan* | *10 mg* | *oraal/zetpil* | *zo nodig bij misselijkheid* | | *ja* | *zo nodig, 3x per dag maximaal (start bij misselijkheid, 8 uur later een volgende tablet of zetpil)* |  |

| Kuur | **Stofnaam** | **Meestvoorkomende merknamen** | **dosering: sterkte+eenheid**  (bij alle medicijnen nodig, ook al is het niet exacte dosering (bv 16mg/kg) | **toedien vorm** | **inname informatie** | **Door patiënt zelf in te nemen** (ja, nee)**?** | **Innametijdstippen** (tijdstip(pen), anders) | **Doel: voorstel om 'onderdeel vd kuur' te vervangen door meer informatieve inhoud** (bv 'chemotherapeuticum' of 'ontstekingsremmer'**)** | belangrijkste bijwerkingen / klachten | Omtrent kuur | #Dagen per cyclus (per behandelkuur) | Max aantal vervolgcycli  (per behandelkuur): | Zo Nodig |
| --- | --- | --- | --- | --- | --- | --- | --- | --- | --- | --- | --- | --- | --- |
| **Dara-vmp** | Daratumumab |  | 16mg/kg | via het infuus | Cyclus 1: Dag 1,8,15,22,29,36 Cyclus 2 t/m 9: Dag 1,22 Vervolg cycli; dag 1 (a 4 weken) | nee |  | doelgerichte therapie |  |  | kuur 1 tot en met 9 42 dagen kuur10 en verder 28 dagen | 12 kuren |  |
|  | Bortezomib | Velcade | 1,3mg/m2 | via een injectie | Cyclus 1:1,4,8,11,22,25,29,32 Cyclus 2 t/m 9: 1,8,22,29 vervolgcycli stop | nee |  | proteasoomremmer |  |  |  |  |  |
|  | Melfalan | Alkeran, Melfalan | 9mg/m2 | oraal | Dag 1 tot en met dag 4 cyclus 1 tot en met 9. vervolgecycli stop | ja | 8 uur | chemotherapie | Misselijkheid; weerstandsvermindering | x |  |  |  |
|  | Prednisolon | Prednison | 60mg/m2 | oraal | Dag 2 tot en met 4, bij het ontbijt innemen kuur 1 tm 9. kuur 10 stop | ja | 8.00 uur | doelgerichte therapie | Gejaagdheid, slecht slapen; rood gelaat; bij bekende suikerziekte: onteregeling. |  |  |  |  |
|  | Paracetamol |  | 1000 mg | oraal | 1 uur voor het infuus 2 tabletten van 500 mg Cyclus 1: Dag 1,8,15,22,29,36 Cyclus 2 t/m 9: Dag 1,22 Vervolg cycli; dag 1 (a 4 weken) | ja | 1 uur voor de kuur | ter voorkoming koorts bij infuus | Geen duidelijke bijwerkingen te verwachten | x |  |  |  |
|  | Prednisolon | Prednison | 100 mg | via het infuus | Cyclus 1: Dag 1,8,15,22,29,36 Cyclus 2 t/m 9: Dag 1,22 Vervolg cycli; dag 1 (a 4 weken) | nee |  | doelgerichte therapie | Gejaagdheid, slecht slapen; rood gelaat; bij bekende suikerziekte: onteregeling. |  |  |  |  |
|  | Clemastine | Tavegyl | 2 mg | via het infuus | 1 uur voor het infuus Cyclus 1: Dag 1,8,15,22,29,36 Cyclus 2 t/m 9: Dag 1,22 Vervolg cycli; dag 1 (a 4 weken) | nee |  | Ter voorkoming allergische reactie | Slaperigheid |  |  |  |  |
|  | Valaciclovir | Zelitrex | 500 mg | oraal | 2 keer per dag, continu | ja | 8 uur en 18 uur | Ter voorkoming gordelroos | Geen duidelijke bijwerkingen te verwachten |  |  |  |  |
|  | Co-trimoxazol |  | 480 mg | oraal | 1 keer per dag, continu | ja | 8 uur | Ter voorkoming longontsteking | Huiduitslag |  |  |  |  |
|  | Prednisolon |  | 20 mg | oraal | cyclus 1: dag 9,10,16,17,23,24,30,31,37,38 cyclus 2 t/m9 dag 23+24 | ja | 8 uur | doelgerichte therapie | Gejaagdheid, slecht slapen; rood gelaat; bij bekende suikerziekte: onteregeling. |  |  |  |  |
|  | Desloratadine | Aerius | 5 mg | oraal | 1x per dag cyclus 1 dag 2,3,9,10,16,17,23,24,30,31,37,38 cyclus 2-9 dag 2,3,23,23 bij kuur 9 ev dag 2 en 3? | ja | 8 uur | Ter voorkoming allergische reactie | Geen duidelijke bijwerkingen te verwachten |  |  |  |  |
|  | Salmeterol/Fluticason | Seretide | 25/125 microgr | inhalatie | Puffje; 2x per dag 1; **zonodig** bij longproblemen | ja | 8 uur en 18 uur | Ter voorkoming van benauwdheid | hartkloppingen |  |  |  | X |
|  | Ondansetron | Zofran | 8 mg | oraal | 2x daags, op dag 1 tm 4 van de cyclus | ja | 1 uur voor de kuur en 18 uur op dag 1 van de kuur | Ter voorkoming van misselijkheid | Hoofdpijn; verstopping | x |  |  |  |
|  | Metoclopramide | primperan | 10 mg | oraal/zetpil | zo nodig bij misselijkheid | ja | zo nodig, 3x per dag maximaal (start bij misselijkheid, 8 uur later een volgende tablet of zetpil) | Ter voorkoming van misselijkheid | Slaperigheid; trillerigheid; diarree |  |  |  | X |
| Kuur | Stofnaam | Meestvorkomende merknamen | |  | inname informatie |  |  | Doel: |  |  |  |  |  |
| **Daratumumab VTD** | Daratumumab |  | 16 mg/kg | via het infuus | Cyclus 1: Dag 1,8,15,22,29,36 Cyclus 2 t/m 9: Dag 1,22 Vervolg cycli; dag 1 (a 4 weken) | nee |  | doelgerichte therapie |  |  | 28? | ? |  |
|  | Bortezomib | Velcade | 1,3mg/kg | via een injectie | dag 1,4,8,11 | nee |  | proteasoomremmer |  |  |  |  |  |
| geen ipova document | Thalidomide | Celgene | 100 mg | oraal | Dagelijks innemen voor de nacht | ja | 22 uur | chemotherapie | Slaperigheid; tinteling handen en voeten |  |  |  |  |
|  | Dexamethason |  | zie F | oraal | kuur 1 en 2 40 mg. 1,2,8,9,15 en 16. Kuur 3 en 4 40 mg dag 1,2 en 20 mg dag 8,9,15 en 16 dag. Innemen bij het ontbijt | ja | 8 uur | doelgerichte therapie | Gejaagdheid, slecht slapen; rood gelaat; bij bekende suikerziekte: onteregeling. | x |  |  |  |
|  | Valaciclovir | Zelitrex | 500 mg | oraal | 2x per dag continu | ja | 8 uur en 18 uur | Ter voorkoming gordelroos | Geen duidelijke bijwerkingen te verwachten |  |  |  |  |
|  | Co-trimoxazol |  | 480 mg | oraal | 1 x per dag continu | ja | 8 uur | Ter voorkoming longontsteking | Huiduitslag |  |  |  |  |
|  | Fluconazol | Diflucan | 50 mg | oraal | 1 x per dag continu | ja | 8 uur | Ter voorkoming van schimmelinfecties. | Geen duidelijke bijwerkingen te verwachten |  |  |  |  |
|  | Nadroparine | Fraxiparine | 0,3 ml | onderhuidse injectie | 1 x per dag; luchtbel niet verwijderen. | ja, of thuiszorg | vast tijdstip op de dag | Ter voorkoming van trombose. | Wat pijn bij injectie; blauwe plekken |  |  |  |  |
|  | Metoclopramide | primperan | 10 mg | oraal/zetpil | zo nodig bij misselijkheid | ja | zo nodig, 3x per dag maximaal (start bij misselijkheid, 8 uur later een volgende tablet of zetpil) | Ter voorkoming van misselijkheid | Slaperigheid; trillerigheid; diarree |  |  |  | X |
|  |  |  |  |  |  |  |  |  |  |  |  |  |  |
| Kuur | Stofnaam | Meestvorkomende merknamen | |  | inname informatie |  |  | helpt bij |  |  |  |  |  |
| **VMP** |  |  |  |  |  |  |  |  |  |  | cyclus duurt 35 dagen | maximaal 9 kuren |  |
| **>75 jaar** | Bortezomib | Velcade | 1,3 mg/m2 | via het infuus | dag 1, 8, 15, 22 | nee |  | proteasoomremmer |  |  |  |  |  |
|  | Melfalan | Alkeran, Melfalan | 9mg/m2 | oraal | Dag 1 tot en met dag 4 | ja | 8 uur | chemotherapie | Misselijkheid; weerstandsvermindering |  |  |  |  |
|  | Prednisolon | Prednison | 1 mg/kg | oraal | Dag 1 tot en met 4 | ja | 8 uur | doelgerichte therapie | Gejaagdheid, slecht slapen; rood gelaat; bij bekende suikerziekte: onteregeling. |  |  |  |  |
|  | Valaciclovir | Zelitrex | 500 mg | oraal | 2x per dag continu | ja | 8 en 18 uur | Ter voorkoming gordelroos. | Geen duidelijke bijwerkingen te verwachten |  |  |  |  |
|  | Co-trimoxazol |  | 480 mg | oraal | 1 x per dag continu | ja | 8 uur | Ter voorkoming longontsteking | Huiduitslag |  |  |  |  |
|  | Ondansetron | Zofran | 8 mg | oraal | dag 1 tot en met 4: 2x daags | ja | 7 uur (1 uur voor Melfalan) en 18 uur | Ter voorkoming van misselijkheid | Hoofdpijn; verstopping | x |  |  |  |
|  | Metoclopramide | Primperan | 10 mg | oraal/zetpil | zo nodig bij misselijkheid | ja | zo nodig, 3x per dag maximaal (start bij misselijkheid, 8 uur later een volgende tablet of zetpil) | Ter voorkoming van misselijkheid | Slaperigheid; trillerigheid; diarree |  |  |  | X |
|  |  |  |  |  |  |  |  |  |  |  |  |  |  |
| **VMP** |  |  |  |  |  |  |  |  |  |  |  |  |  |
| **18 tm 64 jaar** | Bortezomib | Velcade | 1,3 mg/m2 | via een injectie | cyclus 1 tot en met 4:dag 1, 4, 8, 11, 22,25, 29, 32 cyclus 5 en verder dag 1, 8, 22, 29 | nee |  | proteasoomremmer |  |  | 42 dagen | max 9 kuren |  |
|  | Melfalan | Alkeran, Melfalan | 9 mg/m2 | oraal | Dag 1 tot en met dag 4 | ja | dag 1 bij start kuur, dag 2,3,4 bij ontbijt | chemotherapie | Misselijkheid; weerstandsvermindering |  |  |  |  |
|  | Prednisolon | Prednison | 2 mg/kg | oraal | Dag 1 tot en met 4, bij het ontbijt innemen | ja | 8 uur | doelgerichte therapie | Gejaagdheid, slecht slapen; rood gelaat; bij bekende suikerziekte: onteregeling. |  |  |  |  |
|  | Valaciclovir | Zelitrex | 500 mg | oraal | 2x per dag continu | ja | 8 en 18 uur | Ter voorkoming gordelroos. | Geen duidelijke bijwerkingen te verwachten |  |  |  |  |
|  | Co-trimoxazol |  | 480 mg | oraal | 1 x per dag continu | ja | 8 uur | Ter voorkoming longontsteking | Huiduitslag |  |  |  |  |
|  | Zoledroninezuur | Zometa | 4 mg | via het infuus | 1x per 4 weken via het infuus | nee |  | Bescherming van de botten | Griepverschijnselen direct na het infuus |  |  |  |  |
|  | Ondansetron | Zofran | 8 mg | oraal | dag1 tot en met 4 2x daags | ja | 8 en 18 uur | Ter voorkoming van misselijkheid | Hoofdpijn; verstopping | x |  |  |  |
|  | Metoclopramide | Primperan | 10 mg | oraal/zetpil | zo nodig bij misselijkheid | ja | zo nodig, 3x per dag maximaal (start bij misselijkheid, 8 uur later een volgende tablet of zetpil) | Ter voorkoming van misselijkheid | Slaperigheid; trillerigheid; diarree |  |  |  | X |
|  |  |  |  |  |  |  |  |  |  |  |  |  |  |
| **VMP** | Bortezomib | Velcade | 1,3 mg/m2 | via een injectie | cyclus 1 tot en met 4:dag 1, 4, 8, 11, 22,25, 29, 32 cyclus 5 en verder dag 1, 8, 22, 29 | nee |  | proteasoomremmer |  |  | 42 dagen | max 9 kuren |  |
| **65 tm 74 jaar** | Melfalan | Alkeran, Melfalan | 9 mg/m2 | oraal | Dag 1 tot en met dag 4 | ja | dag 1 bij start kuur, dag 2,3,4 bij ontbijt | chemotherapie | Misselijkheid; weerstandsvermindering |  |  |  |  |
|  | Prednisolon | Prednison | 1 mg/kg | oraal | Dag 1 tot en met 4, bij het ontbijt innemen | ja | 8 uur | doelgericht therapie | Gejaagdheid, slecht slapen; rood gelaat; bij bekende suikerziekte: onteregeling. |  |  |  |  |
|  | Valaciclovir | Zelitrex | 500 mg | oraal | 2x per dag continu | ja | 8 en 18 uur | Ter voorkoming gordelroos. | Geen duidelijke bijwerkingen te verwachten |  |  |  |  |
|  | Co-trimoxazol |  | 480 mg | oraal | 1 x per dag continu | ja | 8 uur | Ter voorkoming longontsteking | Huiduitslag |  |  |  |  |
|  | Zoledroninezuur | Zometa | 4 mg | via het infuus | 1x per 4 weken via het infuus | nee |  | Bescherming van de botten | Griepverschijnselen direct na het infuus |  |  |  |  |
|  | Ondansetron | Zofran | 8 mg | oraal | dag1 tot en met 4 2x daags | ja | 8 en 18 uur | Ter voorkoming van misselijkheid | Hoofdpijn; verstopping | x |  |  |  |
|  | Metoclopramide | Primperan | 10 mg | oraal/zetpil | zo nodig bij misselijkheid | ja | zo nodig, 3x per dag maximaal (start bij misselijkheid, 8 uur later een volgende tablet of zetpil) | Ter voorkoming van misselijkheid | Slaperigheid; trillerigheid; diarree |  |  |  | X |
|  |  |  |  |  |  |  |  |  |  |  |  |  |  |
| Kuur | Stofnaam | Meestvorkomende merknamen | |  | inname informatie |  |  | helpt bij |  |  |  |  |  |
| **len/dex** |  |  |  |  |  |  |  |  |  |  | Kuur is 28 dagen | 18 kuren (daar ligt een beslismoment |  |
|  | Lenalidomide | Revlimid | 25 mg | oraal | dag 1 tot en met 21; voor de nacht innemen | ja | 22 uur | doelgerichte therapie | Slaperigheid |  |  |  |  |
|  | Dexamethason |  | 40 mg | oraal | innemen bij het ontbijt, dag 1,8,15 en 22 | ja | 8 uur | doelgerichte therapie |  |  |  |  |  |
|  | Nadroparine | Fraxiparine | 0.3 ml | onderhuidse injectie | 1 x per dag; luchtbel niet verwijderen. Continu | ja, of thuiszorg | vast tijdstip op de dag | Ter voorkoming van trombose. | Pijn bij injectie; blauwe plekken |  |  |  |  |
|  | Co-trimoxazol |  | 480 mg | oraal | 1 keer per dag, continu | ja | 8 uur | Ter voorkoming longontsteking | Huiduitslag |  |  |  |  |
| Kuur | Stofnaam | Meestvorkomende merknamen | |  | inname informatie |  |  | helpt bij |  |  |  |  |  |
| **VD** | Bortezomib | Velcade | 1,3 mg/m2 | via een injectie | dag 1, 4, 8 en 11 | nee |  | proteasoomremmer |  |  | cyclusduur 21 dagen | 8 kuren |  |
|  | Dexamethason |  | 40 mg | oraal | innemen bij het ontbijt, dag 1,8 en 15 | ja | 8 uur | doelgerichte therapie | Gejaagdheid, slecht slapen; rood gelaat; bij bekende suikerziekte: onteregeling. |  |  |  |  |
|  | Omeprazol |  | 40 mg | oraal | 1x daags, continu | ja | 8 uur | Ter voorkoming van maagirritatie |  |  |  |  |  |
|  | Acetylsalicylzuur |  | 80 mg | oraal | 1x daags, continu | ja | 8 uur | Ter voorkoming van trombose | Maagirritatie |  |  |  |  |
|  | Fluconazol | Diflucan | 50 mg | oraal | 1 x per dag continu | ja | 8 uur | Ter voorkoming van schimmelinfecties. | Geen duidelijke bijwerkingen te verwachten |  |  |  |  |
|  | Co-trimoxazol |  | 480 mg | oraal | 1 keer per dag, continu | ja | 8 uur | Ter voorkoming longontsteking | Huiduitslag |  |  |  |  |
|  | Valaciclovir | Zelitrex | 500 mg | oraal | 2x per dag continu | ja | 8 en 18 uur | Ter voorkoming gordelroos. | Geen duidelijke bijwerkingen te verwachten |  |  |  |  |
|  | Metoclopramide | Primperan | 10 mg | oraal/zetpil | zo nodig bij misselijkheid | ja | zo nodig, 3x per dag maximaal (start bij misselijkheid, 8 uur later een volgende tablet of zetpil) | Ter voorkoming van misselijkheid | Slaperigheid; trillerigheid; diarree |  |  |  | X |
| Kuur | Stofnaam | Meestvorkomende merknamen | |  | inname informatie |  |  | helpt bij |  |  |  |  |  |
| **VRD** | Bortezomib | Velcade | 1,3 mg/m2 | via een injectie | dag 1, 4, 8 en 11 | nee |  | proteasoomremmer |  |  | Cyclusduur 28 dagen | 2 kuren |  |
|  | Lenalidomide | Revlimid | 25 mg | oraal | dag 1 tot en met 21 voor de nacht innemen | ja | 22 uur | doelgerichte therapie | Slaperigheid |  |  |  |  |
|  | Dexamethason |  | 20 mg | oraal | innemen bij het ontbijt, dag 1,2,4,5,8,9,11 en 12 | ja | 8 uur | doelgerichte therapie | Gejaagdheid, slecht slapen; rood gelaat; bij bekende suikerziekte: onteregeling. |  |  |  |  |
|  | Omeprazol |  | 40 mg | oraal | 1x daags, continu | ja | 8 uur | Ter voorkoming van maagirritatie |  |  |  |  |  |
|  | Nadroparine | Fraxiparine | 0.3 ml | onderhuidse injectie | 1 x per dag; luchtbel niet verwijderen. Continu | ja, of thuiszorg | vast tijdstip op de dag | Ter voorkoming van trombose. | Pijn bij injectie; blauwe plekken |  |  |  |  |
|  | Acetylsalicylzuur |  | 80 mg | oraal | 1x daags, continu | ja | 8 uur | Ter voorkoming van trombose | Maagirritatie |  |  |  |  |
|  | Fluconazol | Diflucan | 50 mg | oraal | 1 x per dag continu | ja | 8 uur | Ter voorkoming van schimmelinfecties. | Geen duidelijke bijwerkingen te verwachten |  |  |  |  |
|  | Co-trimoxazol |  | 480 mg | oraal | 1 keer per dag, continu | ja | 8 uur | Ter voorkoming longontsteking | Huiduitslag |  |  |  |  |
|  | Valaciclovir | Zelitrex | 500 mg | oraal | 2x per dag continu | ja | 8 en 18 uur | Ter voorkoming gordelroos. | Geen duidelijke bijwerkingen te verwachten |  |  |  |  |
|  | Metoclopramide | Primperan | 10 mg | oraal/zetpil | zo nodig bij misselijkheid | ja | zo nodig, 3x per dag maximaal (start bij misselijkheid, 8 uur later een volgende tablet of zetpil) | Ter voorkoming van misselijkheid | Slaperigheid; trillerigheid; diarree |  |  |  | X |
| Kuur | Stofnaam | Meestvorkomende merknamen | |  | inname informatie |  |  | helpt bij |  |  |  |  |  |
|  | Bortezomib | Velcade | 1,3 mg/m2 | via een injectie | dag 1, 4, 8 en 11 | nee |  | proteasoomremmer |  |  |  |  |  |
| **VTD** | Thalidomide | Celgene | 100 mg | oraal | Dagelijks innemen voor de nacht | ja | 22 uur | chemotherapie | Slaperigheid; tinteling handen en voeten |  | Cyclusduur 28 dagen | 10 kuren |  |
|  | Dexamethason |  | zie F | oraal | kuur 1 en 2 40 mg. 1,2,8,9,15 en 16.Kuur 3 en 4 40 mg dag 1,2 en 20 mg dag 8,9,15 en 16 dag. Innemen bij het ontbijt | ja | 8 uur | doelgerichte therapie | Gejaagdheid, slecht slapen; rood gelaat; bij bekende suikerziekte: onteregeling. |  |  |  |  |
|  | Valaciclovir | Zelitrex | 500 mg | oraal | 2x per dag continu | ja | 8 en 18 uur | Ter voorkoming gordelroos | Geen duidelijke bijwerkingen te verwachten |  |  |  |  |
|  | Co-trimoxazol |  | 480 mg | oraal | 1 keer per dag, continu | ja | 8 uur | Ter voorkoming longontsteking | Huiduitslag |  |  |  |  |
|  | Fluconazol | Diflucan | 50 mg | oraal | 1 x per dag continu | ja | 8 uur | Ter voorkoming van schimmelinfecties. | Geen duidelijke bijwerkingen te verwachten |  |  |  |  |
|  | Nadroparine | Fraxiparine | 0.3ml | onderhuidse injectie | 1 x per dag; luchtbel niet verwijderen. | ja of thuiszorg | vast tijdstip op de dag | Ter voorkoming van trombose. | Wat pijn bij injectie; blauwe plekken |  |  |  |  |
|  | Metoclopramide | Primperan | 10 mg | oraal/zetpil | zo nodig bij misselijkheid | ja | zo nodig, 3x per dag maximaal (start bij misselijkheid, 8 uur later een volgende tablet of zetpil) | Ter voorkoming van misselijkheid | Slaperigheid; trillerigheid; diarree |  |  |  | X |
|  |  |  |  |  |  |  |  |  |  |  |  |  |  |
| Kuur | Stofnaam | Meestvorkomende merknamen | |  | inname informatie |  |  | helpt bij |  |  |  |  |  |
| **Dara-len-dex** | Daratumumab |  | 16 mg/kg | via het infuus | Cyclus 1 & 2 : Dag 1,8,15,22 Cyclus 3 t/m 6: Dag 1,15 Vervolg cycli : Dag 1 | nee |  | doelgerichte therapie |  |  | Cyclusduur 28 dagen | 6 kuren |  |
|  | Lenalidomide | Revlimid | 25 mg | oraal | dag 1 tot en met 21; voor de nacht innemen | ja | 22 uur | doelgerichte therapie | Slaperigheid |  |  |  |  |
|  | Dexamethason |  | zie F | oraal | kuur 1 en 2 dag 1,2,8,9,15,16,22 en 23 20 mg. Kuur 3 tot 6 dag 1,2,15 en 16 20mg dag 8 en 22 40mg vervolg cycli dag 1,2 20 mg dag 8,15,22 40mg innemen bij het ontbijt | ja | 8 uur | doelgerichte therapie | Gejaagdheid, slecht slapen; rood gelaat; bij bekende suikerziekte: onteregeling. |  |  |  |  |
|  | Paracetamol |  | 1000 mg | oraal | 1 uur voor het infuus 2 tabletten van 500 mg | ja | 1 uur voor de kuur | ter voorkoming koorts bij infuus | Geen duidelijke bijwerkingen te verwachten |  |  |  |  |
|  | Clemastine | Tavegyl | 2 mg | intraveneus | 1 uur voor het infuus | nee |  | Ter voorkoming allergische reactie | Slaperigheid |  |  |  |  |
|  | Nadroparine | Fraxiparine | 0.3 ml | onderhuidse injectie | 1 x per dag; luchtbel niet verwijderen. Continu | ja of thuiszorg | vast tijdstip op de dag | Ter voorkoming van trombose. | Pijn bij injectie; blauwe plekken |  |  |  |  |
|  | Valaciclovir | Zelitrex | 500 mg | oraal | 2x per dag continu | ja | 8 uur en 18 uur | Ter voorkoming gordelroos. | Geen duidelijke bijwerkingen te verwachten |  |  |  |  |
|  | Co-trimoxazol |  | 480 mg | oraal | 1 keer per dag, continu | ja | 8 uur | Ter voorkoming longontsteking | Huiduitslag |  |  |  |  |
|  | Fluconazol | Diflucan | 50 mg | oraal | 1 x per dag continu | ja | 8 uur | Ter voorkoming van schimmelinfecties. | Geen duidelijke bijwerkingen te verwachten |  |  |  |  |
|  | Omeprazol |  | 20 mg | oraal | 1x daags, continu | ja | 8 uur | Ter voorkoming van maagirritatie |  |  |  |  |  |
|  | Desloratadine |  | 5 mg | oraal | zo nodig kuur 1 dag 1,2,8,9,15,16, 22 en 23. kuur 2 dag 1,2 en 15 en 16 | ja | 8 uur | Ter voorkoming allergische reactie | Geen duidelijke bijwerkingen te verwachten |  |  |  | X |
|  | Salmeterol/Fluticason | Seretide | 25/125 microgr | inhalatie | Puffje; 2x per dag 1; zonodig bij longproblemen | ja | 8 en 18 uur | Ter voorkoming van benauwdheid | hartkloppingen |  |  |  | X |
| Kuur | Stofnaam | Meestvorkomende merknamen | |  | inname informatie |  |  | helpt bij |  |  |  |  |  |
| **Lenalidomide onderhoud** | |  |  |  |  |  |  |  |  |  |  |  |  |
|  | Lenalidomide | Revlimid | 10 mg | oraal | dag 1 tot en met 21 10 mg; voor de nacht innemen | ja | 22 uur | doelgerichte therapie | Slaperigheid |  | Cyclusduur 28 dagen | tot progressie 24 kuren |  |
|  | Nadroparine | Fraxiparine | 0.3 ml | onderhuidse injectie | 1 x per dag; luchtbel niet verwijderen. | ja of thuiszorg | vast tijdstip op de dag | Ter voorkoming van trombose. | Wat pijn bij injectie; blauwe plekken |  |  |  |  |
|  |  |  |  |  |  |  |  |  |  |  |  |  |  |
|  |  |  |  |  |  |  |  |  |  |  |  |  |  |
| Kuur | Stofnaam | Meestvorkomende merknamen | |  | inname informatie |  |  | helpt bij |  |  |  |  |  |
| **KRd** | Carfilzomib |  | Kuur 1 dag 1 en 2 20 mg/m2 (max 44mg) dag 8,9,15 en 16 27mg/m2 (max 60mg) kuur 2 en volgende 27mg/m2 (max 60mg) | via het infuus | dag 1,2,8,9,15 en 16 | nee |  | proteasoomremmer |  |  | Cyclusduur 28 dagen | max 12 kuren |  |
|  | Lenalidomide | Revlimid | 25 mg | oraal | dag 1 tot en met 21; voor de nacht innemen | ja | 22 uur | doelgerichte therapie | Slaperigheid |  |  |  |  |
|  | Dexamethason |  | 20 mg | oraal | innemen bij het ontbijt; dag 1,2,8,9,15,16 en 22 | ja | 8 uur | doelgerichte therapie |  |  |  |  |  |
|  |  |  |  |  |  |  |  |  |  |  |  |  |  |
|  | Valaciclovir | Zelitrex | 500 mg | oraal | 2x per dag continu | ja | 8 en 22 uur | Ter voorkoming gordelroos. | Geen duidelijke bijwerkingen te verwachten |  |  |  |  |
|  | Ciprofloxacin |  | 500 mg | oraal | 2x per dag continu | ja | 8 en 22 uur | Ter voorkoming van infecties |  |  |  |  |  |
|  | Fluconazol | Diflucan | 50 mg | oraal | 1 x per dag continu | ja | 8 uur | Ter voorkoming van schimmelinfecties. | Geen duidelijke bijwerkingen te verwachten |  |  |  |  |
|  | Co-trimoxazol |  | 480 mg | oraal | 1 keer per dag, continu | ja | 8 uur | Ter voorkoming longontsteking | Huiduitslag |  |  |  |  |
|  |  |  |  |  |  |  |  |  |  |  |  |  |  |
|  | Allopurinol |  | 200 mg | oraal | zo nodig 1x daags | ja | 8 uur | Bevordering uitscheiding afbraakproducten | Huiduitslag |  |  |  | X |
|  | Rasburicase |  | 3mg | via het infuus | zo nodig via infuus | nee | 30 min voor start van chemotherapie of prednisolon | Bevordering uitscheiding afbraakproducten |  |  |  |  | X |
|  | Metoclopramide | Primperan | 10 mg | oraal/zetpil | zo nodig bij misselijkheid | ja | zo nodig, 3x per dag maximaal (start bij misselijkheid, 8 uur later een volgende tablet of zetpil) | Ter voorkoming van misselijkheid | Slaperigheid; trillerigheid; diarree |  |  |  | X |
| Kuur | Stofnaam | Meestvorkomende merknamen | |  | inname informatie |  |  | helpt bij |  |  |  |  |  |
| **Erd** | Elotuzumab |  | 10 mg/kg | via het infuus | kuur 1 en 2 dag 1,8,15,22; kuur 3 en verder dag 1 en 15 | nee |  | doelgerichte therapie |  |  | Cyclusduur 28 dagen | tot progressie 24 kuren |  |
|  | Lenalidomide | Revlimid | 25 mg | oraal | dag 1 tot en met 21; voor de nacht innemen | ja | 22 uur | doelgerichte therapie | Slaperigheid |  |  |  |  |
|  | Dexamethason |  | 28 mg | oraal | kuur 1 en 2: 28 mg dag 1,8,15,22 kuur 3 en verder: dag 1 en 15 28mg en dag 8 en 22 40mg | ja | 8 uur | doelgerichte therapie |  |  |  |  |  |
|  | Dexamethason |  | 8 mg | via het infuus | via het infuus | nee |  | doelgerichte therapie |  |  |  |  |  |
|  | Clemastine | Tavegyl | 2 mg | via het infuus | Via het infuus | nee |  | Ter voorkoming allergische reactie | Slaperigheid |  |  |  |  |
|  | Ranitidine |  | 50 mg | via het infuus | via het infuus | nee |  | Ter voorkoming allergische reactie |  |  |  |  |  |
|  | paracetamol |  | 1000 mg | oraal | 45-90 min voor infuus 2 tabletten van 500 mg | ja | 45-90 min voor het infuus | ter voorkoming koorts bij infuus | Geen duidelijke bijwerkingen te verwachten | x |  |  |  |
|  | Nadroparine | Fraxiparine | 0.3 ml | onderhuidse injectie | 1 x per dag; luchtbel niet verwijderen. Continu | ja of thuiszorg | vast tijdstip op de dag | Ter voorkoming van trombose. | Pijn bij injectie; blauwe plekken |  |  |  |  |
|  | Co-trimoxazol |  | 480 mg | oraal | 1 keer per dag, continu | ja | 8 uur | Ter voorkoming longontsteking | Huiduitslag |  |  |  |  |
|  |  |  |  |  |  |  |  |  |  |  |  |  |  |
| Kuur | Stofnaam | Meestvorkomende merknamen | |  | inname informatie |  |  | helpt bij |  |  |  |  |  |
| **Ird** | Ixazomib |  | volgens voorschrift |  | dag 1,8,15 | ja | ? | doelgerichte therapie? | Nog geen schema in i-Prova in studie verband ixazomib of placebo. |  | Cyclusduur 28 dagen | max 9 kuren |  |
|  | Lenalidomide | Revlimid | volgens voorschrift | oraal | dag 1 tot 28 | ja | 22 uur | doelgerichte behandeling |  |  |  |  |  |
|  | Dexamethason |  | volgens voorschrift | oraal | dag 1,8,15,22 | ja | 8 uur | doelgerichte therapie |  |  |  |  |  |
|  |  |  |  |  |  |  |  |  |  |  |  |  |  |
| Kuur | Stofnaam | Meestvorkomende merknamen | |  | inname informatie |  |  | helpt bij |  |  |  |  |  |
| **REP** | Lenalidomide | Revlimid | 10 mg | oraal | dag 1 tot en met 21 15mg; voor de nacht innemen | ja | 22 uur | doelgerichte therapie | Slaperigheid |  | Cyclusduur 28 dagen | tot progressie 24 kuren |  |
|  | Cyclofosfamide | Endoxan | 50-100 mg afhankelijk van beenmerg reserve (REP 50 en de REP100 kuur aanmaken) | oraal | 1x per dag, continu 'S ochtend innemen. | ja | 8 uur | chemotherapie | Evt misselijkheid of wat haaruitval |  |  |  |  |
|  | Prednisolon |  | Kuur 1 en 2 20 mg, overige kuren 10 mg | oraal | 1x per dag, continu 'S ochtend innemen | ja | 8 uur | doelgerichte therapie | Gejaagdheid, slecht slapen; rood gelaat; bij bekende suikerziekte: onteregeling. |  |  |  |  |
|  | Omeprazol |  | 40 mg | oraal | 1x daags, continu | ja | 8 uur | Ter voorkoming van maagirritatie |  |  |  |  |  |
|  | Acetylsalicylzuur |  | 80 mg | oraal | 1x daags, continu | ja | 8 uur | Ter voorkoming van trombose | Maagirritatie |  |  |  |  |
|  | Co-trimoxazol |  | 480 mg | oraal | 1 keer per dag, continu | ja | 8 uur | Ter voorkoming longontsteking | Huiduitslag |  |  |  |  |
|  | Fluconazol | Diflucan | 50 mg | oraal | 1 x per dag continu | ja | 8 uur | Ter voorkoming van schimmelinfecties. | Geen duidelijke bijwerkingen te verwachten |  |  |  |  |
|  | Valaciclovir | Zelitrex | 500 mg | oraal | 2x per dag continu | ja | 8 en 18 uur | Ter voorkoming gordelroos. | Geen duidelijke bijwerkingen te verwachten |  |  |  |  |
|  | Metoclopramide | Primperan | 10 mg | oraal/zetpil | zo nodig bij misselijkheid | ja | zo nodig, 3x per dag maximaal (start bij misselijkheid, 8 uur later een volgende tablet of zetpil) | Ter voorkoming van misselijkheid | Slaperigheid; trillerigheid; diarree |  |  |  | X |
|  |  |  |  |  |  |  |  |  |  |  |  |  |  |
| Kuur | Stofnaam | Meestvorkomende merknamen | |  | inname informatie |  |  | helpt bij |  |  |  |  |  |
| **Kd (Car-dex)** | Carfilzomib |  | Kuur 1 dag 1 en 2 20 mg/m2 (max 44mg) dag 8,9,15 en 16 56mg/m2 (max 123,2mg) kuur 2 en volgende 56 mg/m2 (max 123,2mg) | via het infuus | Infuus; dag 1,2,8,9,15 en 16 | nee |  | proteasoomremmer |  |  | Cyclusduur 28 dagen | tot progressie 24 kuren |  |
|  | Dexamethason |  | 20 mg | oraal | Dag 1, 2, 8, 9, 15, 16, bij het ontbijt innemen | ja | 8 uur | doelgerichte therapie | Gejaagdheid, slecht slapen; rood gelaat; bij bekende suikerziekte: onteregeling. |  |  |  |  |
|  | Ciprofloxacin |  | 500 mg | oraal | 2x per dag continu | ja | 8 en18 uur | Ter voorkoming van infecties |  |  |  |  |  |
|  | Fluconazol | Diflucan | 50 mg | oraal | 1 x per dag continu | ja | 8 uur | Ter voorkoming van schimmelinfecties. | Geen duidelijke bijwerkingen te verwachten |  |  |  |  |
|  | Valaciclovir | Zelitrex | 500 mg | oraal | 2x per dag continu | ja | 8 en 18 uur | Ter voorkoming gordelroos. | Geen duidelijke bijwerkingen te verwachten |  |  |  |  |
|  | Co-trimoxazol |  | 480 mg | oraal | 1 keer per dag, continu | ja | 8 uur | Ter voorkoming longontsteking | Huiduitslag |  |  |  |  |
|  | Metoclopramide | Primperan | 10 mg | oraal/zetpil | zo nodig bij misselijkheid | ja | zo nodig, 3x per dag maximaal (start bij misselijkheid, 8 uur later een volgende tablet of zetpil) | Ter voorkoming van misselijkheid | Slaperigheid; trillerigheid; diarree |  |  |  | X |
|  |  |  |  |  |  |  |  |  |  |  |  |  |  |
|  |  |  |  |  |  |  |  |  |  |  |  |  |  |
|  |  |  |  |  |  |  |  |  |  |  |  |  |  |
| Kuur | Stofnaam | Meestvorkomende merknamen | |  | inname informatie |  |  | helpt bij |  |  |  |  |  |
| **Epd < 75 jaar** | Pomalidomide | Imnovid | 4 mg | oraal | dag 1 tot en met 21 | ja | 22 uur | doelgerichte therapie |  |  | 28 dagen | tot progressie 24 kuren |  |
| **Epd > 75 jaar** | Dexamethason |  | 1x daags 40 mg (boven de 75 jaar 20 mg) | oraal | dag 1,8,15,22 innemen bij ontbijt | ja | 8 uur | doelgerichte therapie |  |  |  |  |  |
|  | Acetylsalicylzuur |  | 80 mg | oraal | 1x daags, continu | ja | 8 uur | Ter voorkoming van trombose | Maagirritatie |  |  |  |  |
|  | Co-trimoxazol |  | 480 mg | oraal | 1 keer per dag, continu | ja | 8 uur | Ter voorkoming longontsteking | Huiduitslag |  |  |  |  |
|  | Metoclopramide | Primperan | 10 mg | oraal/zetpil | zo nodig bij misselijkheid | ja | zo nodig, 3x per dag maximaal (start bij misselijkheid, 8 uur later een volgende tablet of zetpil) | Ter voorkoming van misselijkheid | Slaperigheid; trillerigheid; diarree |  |  |  | X |
|  |  |  |  |  |  |  |  |  |  |  |  |  |  |
| Kuur | Stofnaam | Meestvorkomende merknamen | |  | inname informatie |  |  | helpt bij |  |  |  |  |  |
| **Pomalidomide +cyclofosfamide ("PEP")** | Pomalidomide | Imnovid | 4 mg | oraal | 1 x per dag; dag 1 tm 21 | ja | 22 uur | doelgerichte therapie | Slaperigheid |  |  |  |  |
| geen iprova document | Cyclofosfamide | Endoxan |  | oraal | 1x per dag, continu. 'S ochtend innemen | ja | 8 uur | Chemotherapie | Evt misselijkheid of wat haaruitval |  |  |  |  |
|  | Prednisolon |  | volgens voorschrift? | oraal | 1x per dag, continu. 'S ochtend innemen | ja | 8 uur | doelgerichte therapie | Gejaagdheid, slecht slapen; rood gelaat; bij bekende suikerziekte: onteregeling. |  |  |  |  |
|  | Omeprazol |  | 40 mg | oraal | 1x daags, continu | ja | 8 uur | Ter voorkoming van maagirritatie |  |  |  |  |  |
|  | Acetylsalicylzuur |  | 80 mg | oraal | 1x daags, continu | ja | 8 uur | Ter voorkoming van trombose | Maagirritatie |  |  |  |  |
|  | Co-trimoxazol |  | 480 mg | oraal | 1 keer per dag, continu | ja | 8 uur | Ter voorkoming longontsteking | Huiduitslag |  |  |  |  |
|  | Fluconazol | Diflucan | 50 mg | oraal | 1 x per dag continu | ja | 8 uur | Ter voorkoming van schimmelinfecties. | Geen duidelijke bijwerkingen te verwachten |  |  |  |  |
|  | Valaciclovir | Zelitrex | 500 mg | oraal | 2x per dag continu | ja | 8 en 18 uur | Ter voorkoming gordelroos. | Geen duidelijke bijwerkingen te verwachten |  |  |  |  |
|  | Metoclopramide | Primperan | 10 mg | oraal/zetpil | zo nodig bij misselijkheid | ja | zo nodig, 3x per dag maximaal (start bij misselijkheid, 8 uur later een volgende tablet of zetpil) | Ter voorkoming van misselijkheid | Slaperigheid; trillerigheid; diarree |  |  |  | X |
|  |  |  |  |  |  |  |  |  |  |  |  |  |  |
| Kuur | Stofnaam | Meestvorkomende merknamen | |  | inname informatie |  |  | helpt bij |  |  |  |  |  |
| **Pomalidomide +carfilzomib, dexamethason** | Pomalidomide | Imnovid | 4 mg | oraal | 1 x per dag; dag 1 tm 21 | ja | 22 uur | doelgerichte therapie | Slaperigheid |  | Cylclusduur 28 dagen | tot progressie of stamceltransplantatie 24 kuren |  |
|  | Carfilzomib |  | zie F | via het infuus | Kuur1 : 20 mg/m2 dag dag 1 en 2 36mg/m2 dag 8,9,15 en 16 overige kuren 36mg/m2 dag 1,2,8,9,15,en16 | nee |  | proteasoomremmer |  |  |  |  |  |
|  | Dexamethason |  | 20 mg | oraal | Dag 1, 2, 8, 9, 15, 16, bij het ontbijt innemen | ja | 8 uur | doelgerichte therapie | Gejaagdheid, slecht slapen; rood gelaat; bij bekende suikerziekte: onteregeling. |  |  |  |  |
|  | Nadroparine | Fraxiparine | 0.3 ml | onderhuidse injectie | 1 x per dag; luchtbel niet verwijderen. Continu | ja of thuiszorg | vast tijdstip op de dag | Ter voorkoming van trombose. | Pijn bij injectie; blauwe plekken |  |  |  |  |
|  | Ciprofloxacin |  | 500 mg | oraal | 2x per dag continu | ja | 8 en 18 uur | ter voorkoming van ontsteking | misselijkheid/ diaree klachten |  |  |  |  |
|  | Valaciclovir | Zelitrex | 500 mg | oraal | 2x per dag continu | ja | 8 en 18 uur | Ter voorkoming gordelroos. | Geen duidelijke bijwerkingen te verwachten |  |  |  |  |
|  | Fluconazol | Diflucan | 50 mg | oraal | 1 x per dag continu | ja | 8 uur | Ter voorkoming van schimmelinfecties. | Geen duidelijke bijwerkingen te verwachten |  |  |  |  |
|  | Metoclopramide | Primperan | 10 mg | oraal/zetpil | zo nodig bij misselijkheid | ja | zo nodig, 3x per dag maximaal (start bij misselijkheid, 8 uur later een volgende tablet of zetpil) | Ter voorkoming van misselijkheid | Slaperigheid; trillerigheid; diarree |  |  |  | X |
|  |  |  |  |  |  |  |  |  |  |  |  |  |  |
| Kuur | Stofnaam | Meestvorkomende merknamen | |  | inname informatie |  |  | helpt bij |  |  |  |  |  |
| **Cyclofosfamide mobilisatie** | Cyclofosfamide | Endoxan | 1000 mg/m2 | via het infuus | dag 1, via het infuus | nee |  | chemotherapie |  |  | Cyclusduur 12 dagen met mogelijkheid om dag 13 en 14 op te kunnen plussen | 1 kuur |  |
|  | Neupogen | Filgrastim | volgens voorschrift 2x daags 5 Ug/kg/dag | onderhuidse injectie | dag 7,8,9,10,11 8 uur en 20 uur. Dag 12 6.30 evt ook 20 uur en dag 13 6.30 evt ook dag 14 6.30 injectie subcutaan | ja of thuiszorg | 8 uur en 20 uur (op de dag van stamcelafname 6.30 uur) | mobiliseren van de stamcellen |  |  |  |  |  |
|  | Ondansetron |  | 8 mg | oraal | dag 1, 2x per dag. 1 uur voor infuus en bij avondeten | ja | 1 uur voor de kuur en 18 uur | Ter voorkoming van misselijkheid |  | x |  |  |  |
|  | Metoclopramide | Primperan | 10 mg | oraal/zetpil | zo nodig bij misselijkheid | ja | zo nodig, 3x per dag maximaal (start bij misselijkheid, 8 uur later een volgende tablet of zetpil) | Ter voorkoming van misselijkheid | Slaperigheid; trillerigheid; diarree |  |  |  |  |

## S3 Questionnaires

**EQ-5D-5L**

Zet bij iedere groep in de lijst hieronder een kruisje in het hokje achter de zin die het best past bij uw eigen gezondheidstoestand vandaag.

**Mobiliteit**

Ik heb geen problemen met lopen 

Ik heb een beetje problemen met lopen 

Ik heb matige problemen met lopen 

Ik heb ernstige problemen met lopen 

Ik ben niet in staat niet om te lopen 

**Zelfzorg**

Ik heb geen problemen met mijzelf wassen of aankleden 

Ik heb een beetje problemen met mijzelf wassen of aankleden 

Ik heb matige problemen met mijzelf wassen of aankleden 

Ik heb ernstige problemen met mijzelf wassen of aankleden 

Ik ben niet in staat mijzelf te wassen of aan te kleden 

**Dagelijkse activiteiten** *(bijv. werk, studie, huishouden,*

*gezins- en vrijetijdsactiviteiten)*

Ik heb geen problemen met mijn dagelijkse activiteiten 

Ik heb een beetje problemen met mijn dagelijkse activiteiten 

Ik heb matige problemen met mijn dagelijkse activiteiten 

Ik heb ernstige problemen met mijn dagelijkse activiteiten 

Ik ben niet in staat mijn dagelijkse activiteiten uit te voeren 

**Pijn/ongemak**

Ik heb geen pijn of ongemak 

Ik heb een beetje pijn of ongemak 

Ik heb matige pijn of ongemak 

Ik heb ernstige pijn of ongemak 

Ik heb extreme pijn of ongemak 

**Angst/somberheid**

Ik ben niet angstig of somber 

Ik ben een beetje angstig of somber 

Ik ben matig angstig of somber 

Ik ben erg angstig of somber 

Ik ben extreem angstig of somber 

Om mensen te helpen bij het aangeven hoe goed of hoe slecht een gezondheidstoestand is, hebben we een meetschaal (te vergelijken met een thermometer) gemaakt. Op de meetschaal hiernaast betekent “100” de beste gezondheidstoestand die u zich kunt voorstellen, en “0” de slechtste gezondheidstoestand die u zich kunt voorstellen.

9 0

8 0

7 0

6 0

5 0

4 0

3 0

2 0

1 0

100

Slechtst

voorstelbare

gezondheidstoestand

0

Best

voorstelbare

gezondheidstoestand

We willen u vragen op deze meetschaal aan te geven hoe goed of hoe slecht volgens u uw eigen gezondheidstoestand vandaag is. Trek een lijn van het hokje hieronder naar het punt op de meetschaal dat volgens u aangeeft hoe goed of hoe slecht uw gezondheidstoestand vandaag is.

**Uw gezondheidstoestand**

**vandaag**

MARS-5


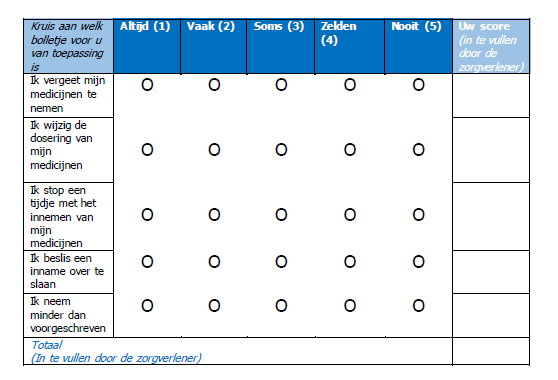


<https://www.hovumc.nl/w/images/f/ff/Sdm-q-9_dutch_version.pdf>

## S4 PROMs and PREMs


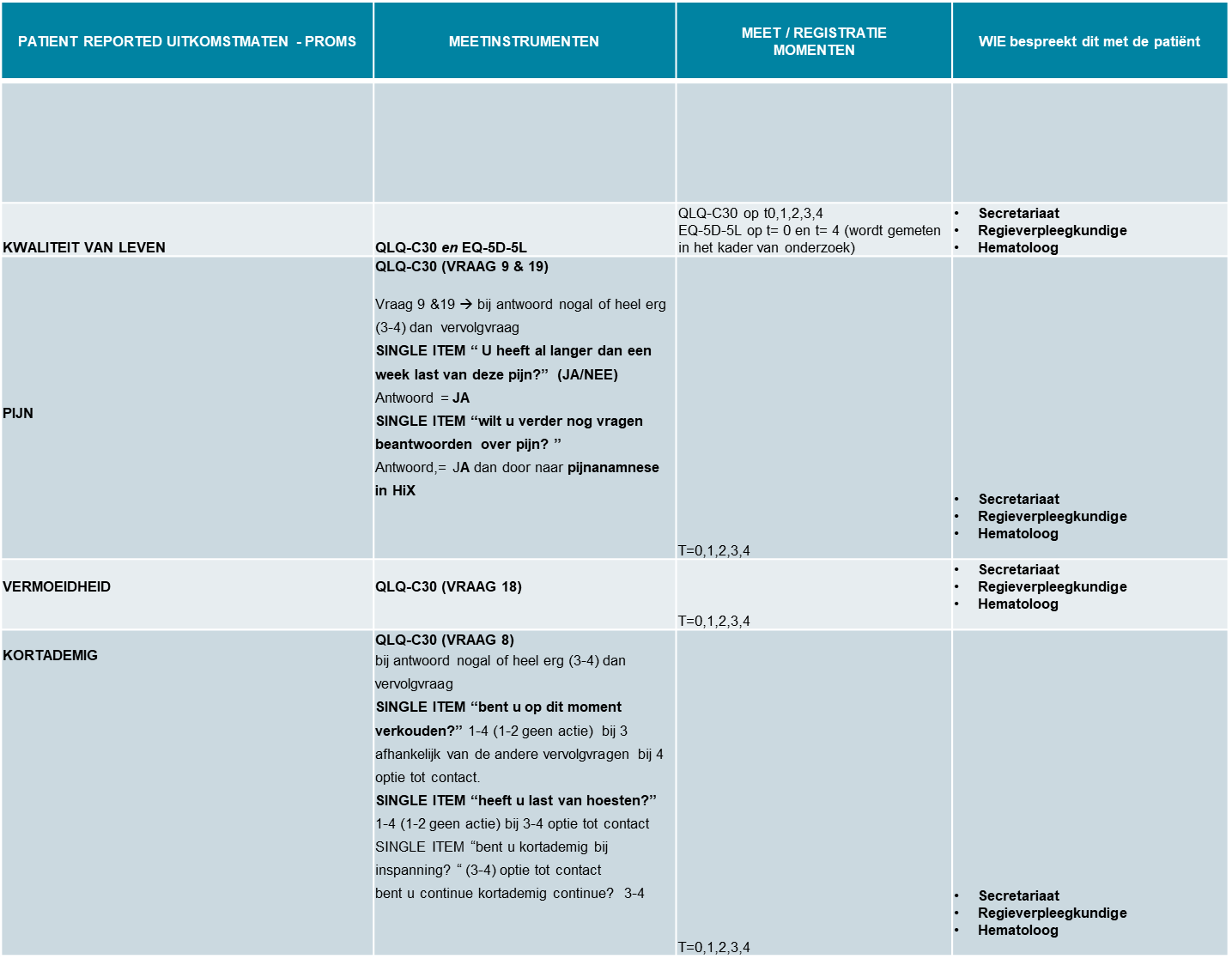


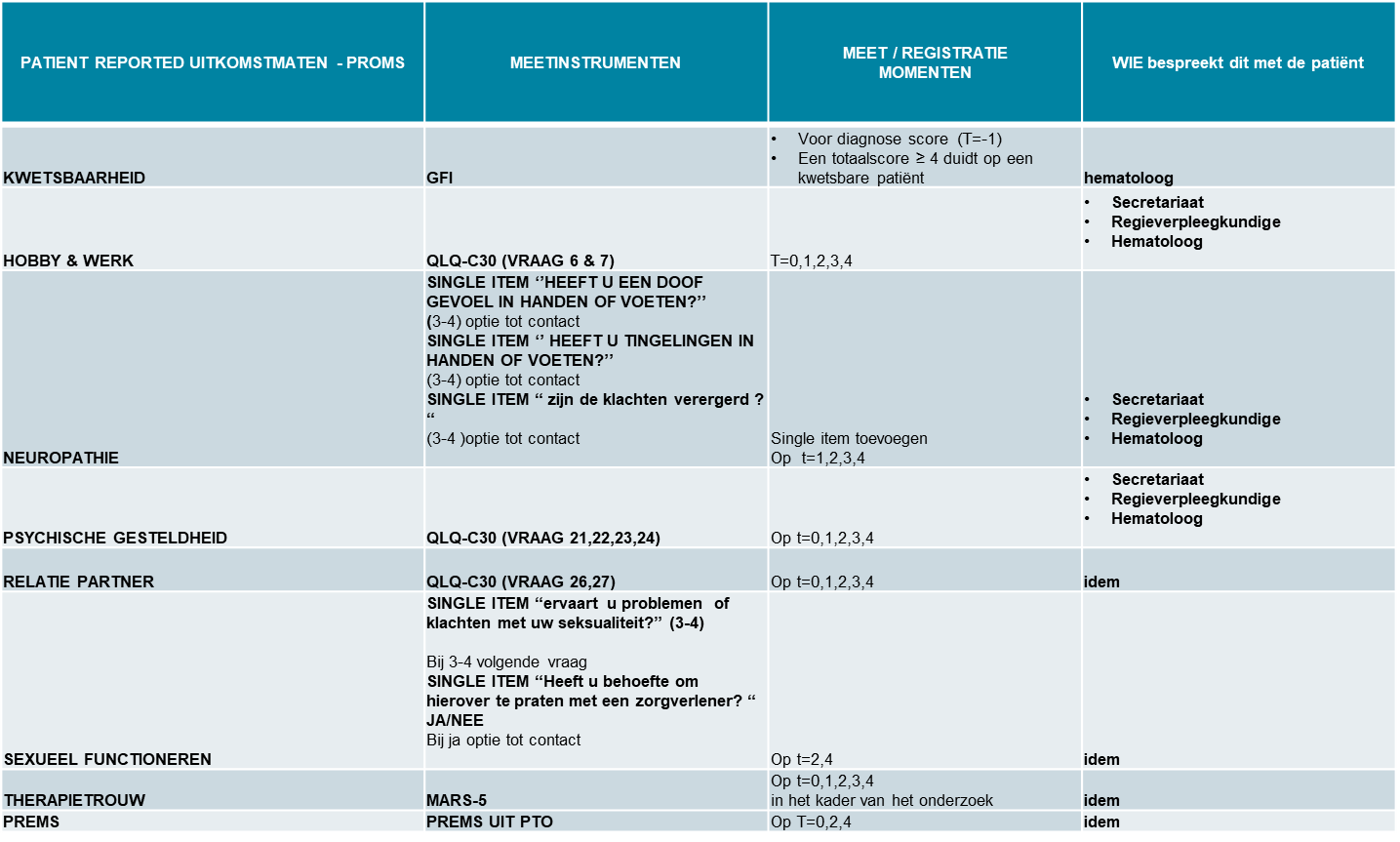


**PREMs**


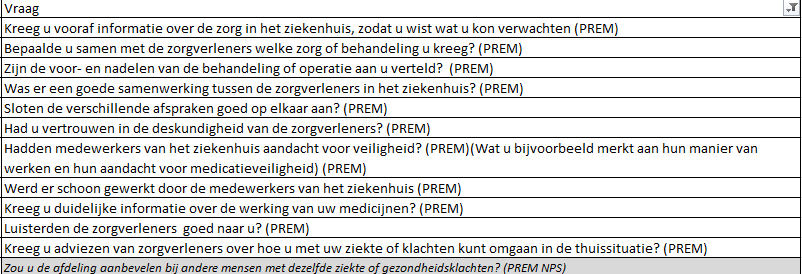


## S5 Clinical outcomes


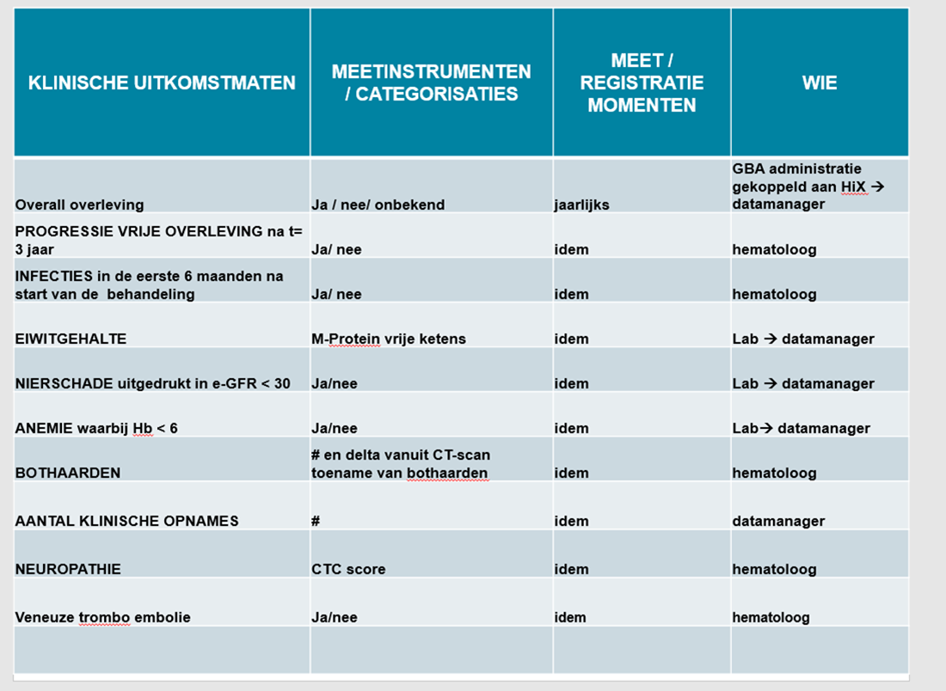


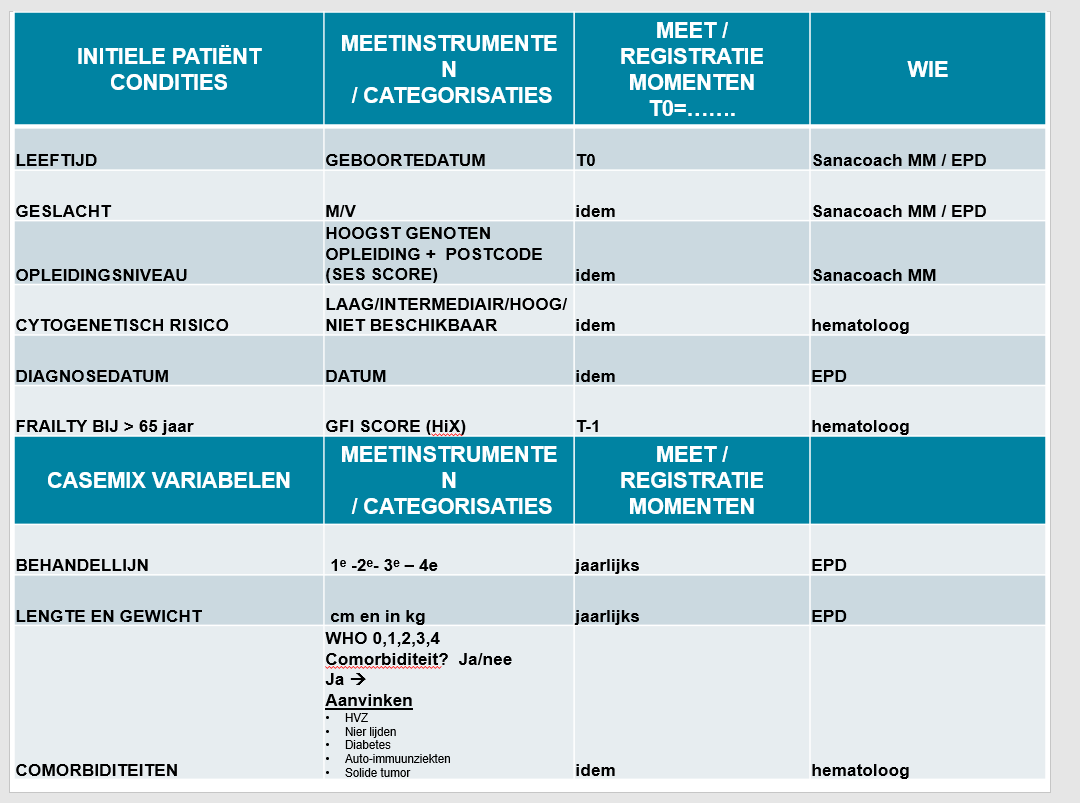


## S6 Procedures


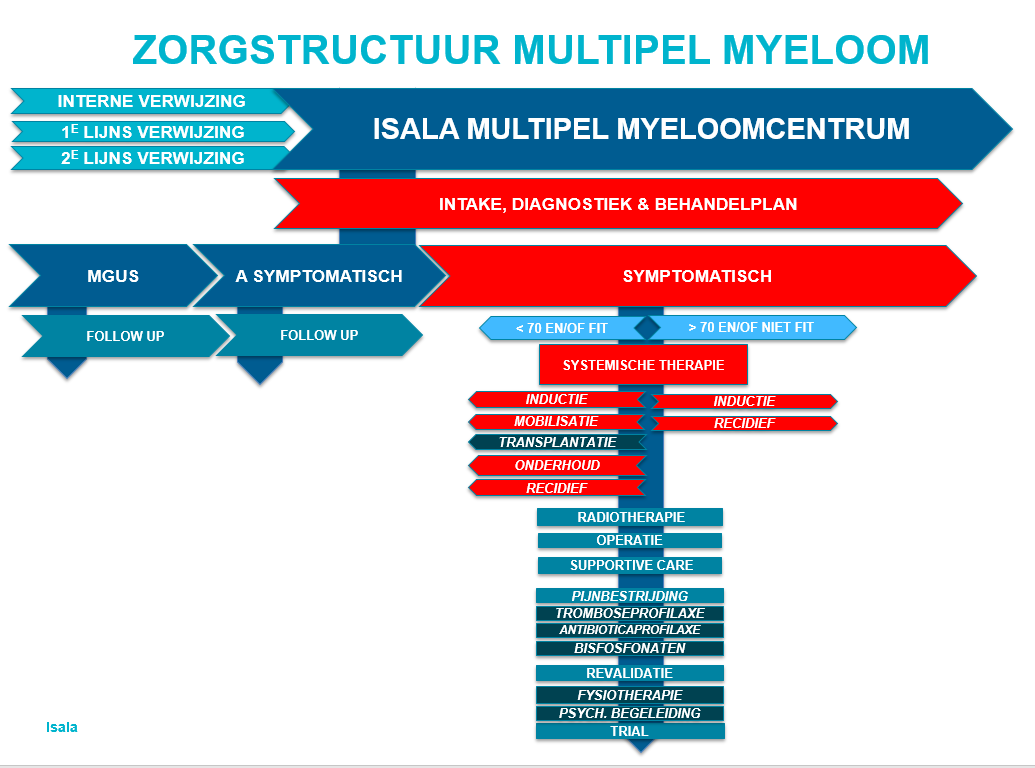


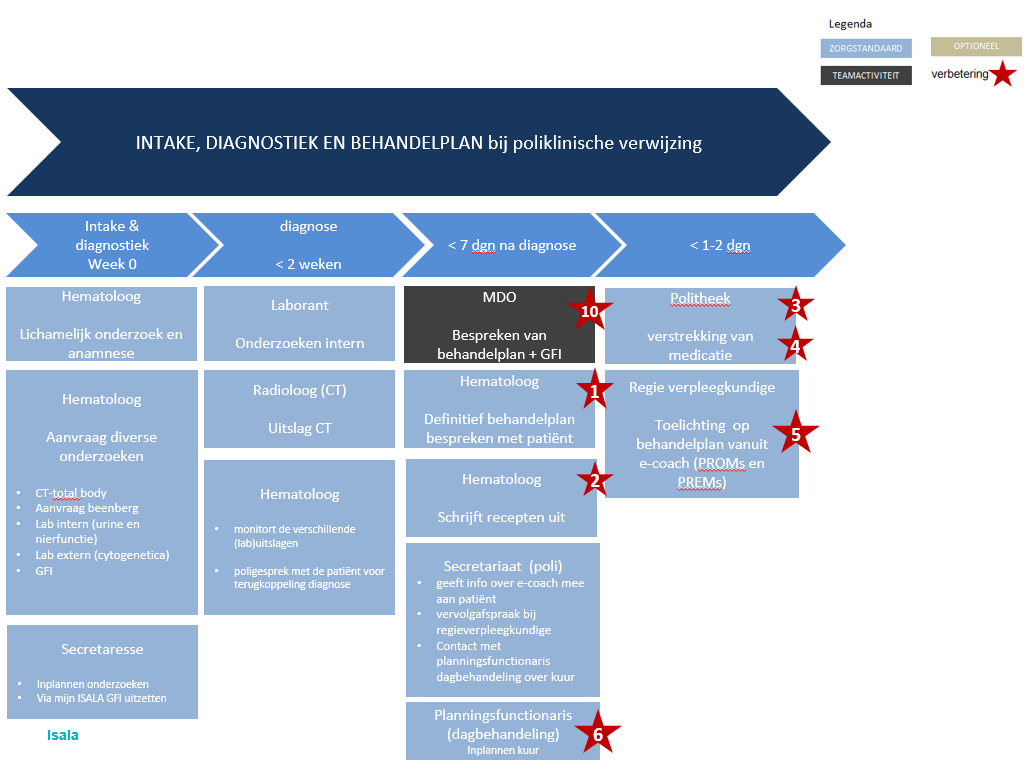


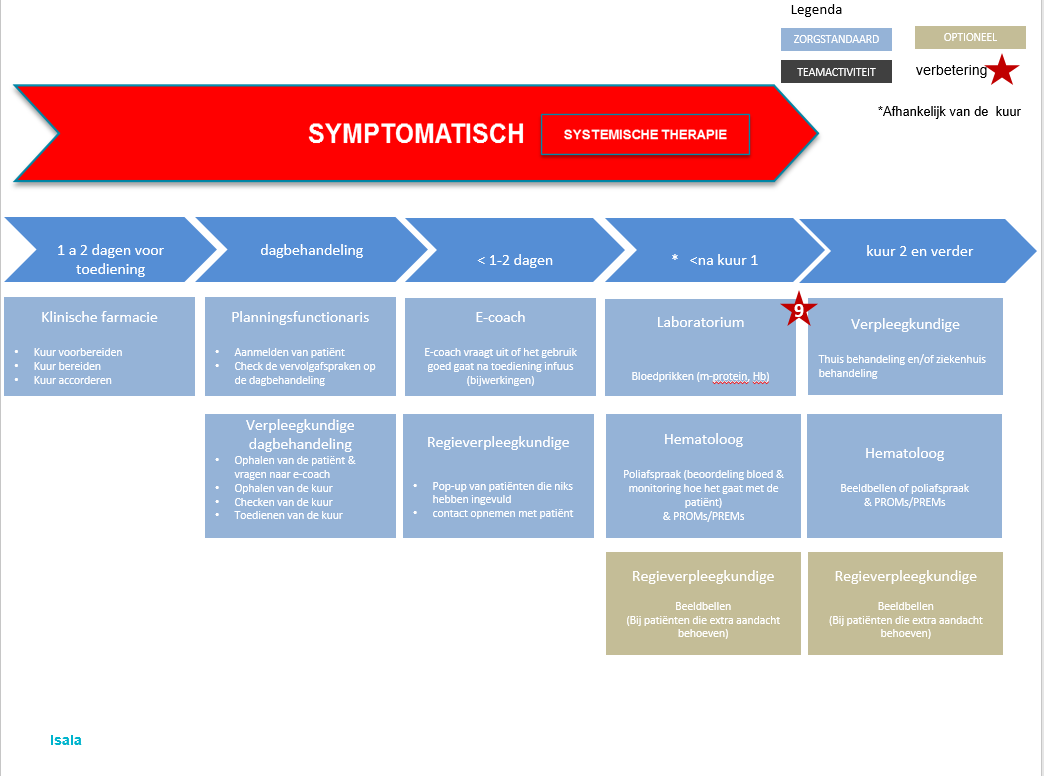


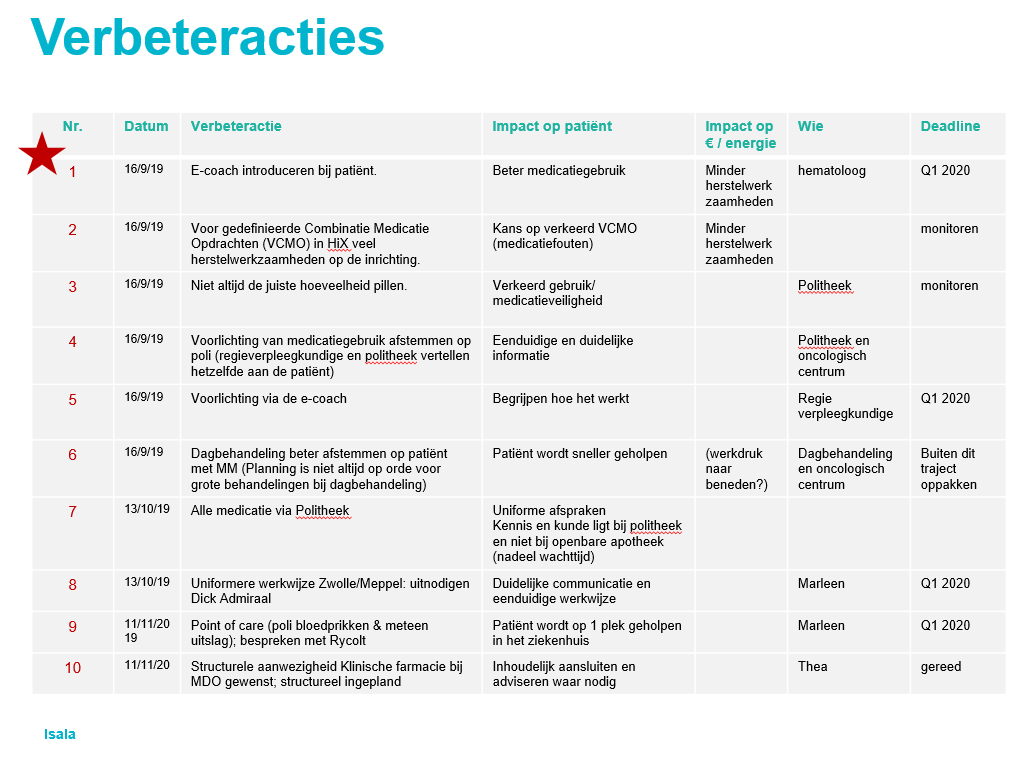


## S7 Pill count


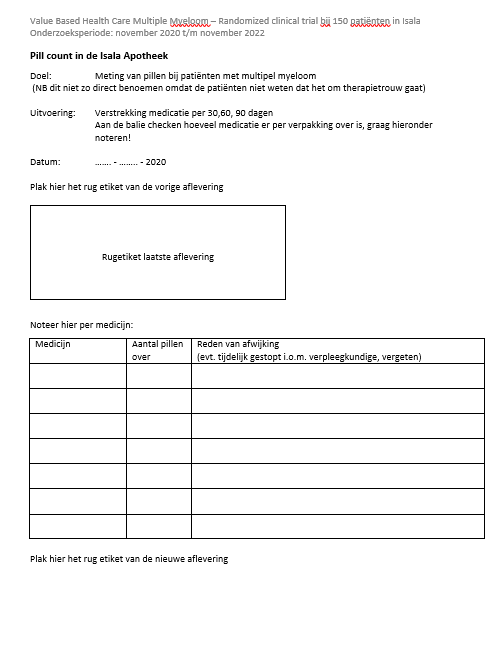


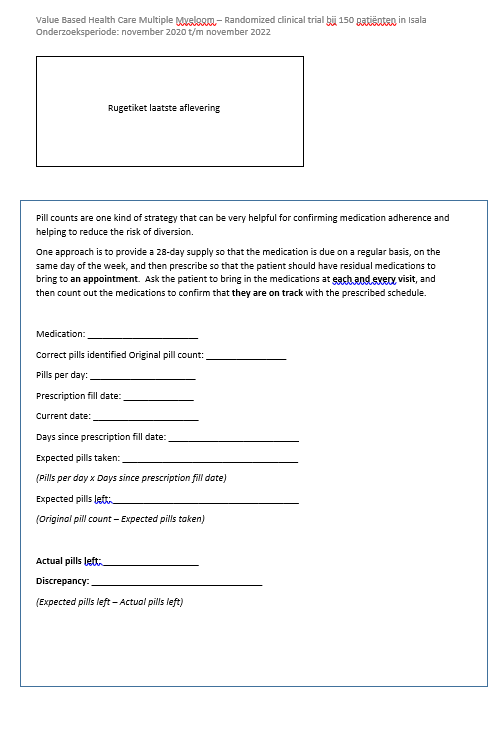


## S8 Informed consent ITUMM study

**Bijlage ITUMM study: Toestemmingsformulier proefpersoon WMO studies**

- Ik weet dat meedoen vrijwillig is. Ook weet ik dat ik op ieder moment kan beslissen om toch niet mee te doen of te stoppen met het onderzoek. Daarvoor hoef ik geen reden te geven.
- Ik weet dat sommige mensen mijn gegevens kunnen inzien.
- Ik geef toestemming om mijn gegevens op de onderzoekslocatie nog [5] jaar na dit onderzoek te bewaren.
- Ik wil meedoen aan dit onderzoek.

Naam proefpersoon/patiënt:

Handtekening: Datum : __ / __ / __

-----------------------------------------------------------------------------------------------------------------

Ik verklaar dat ik deze proefpersoon/patiënt volledig heb geïnformeerd over het genoemde onderzoek.

Als er tijdens het onderzoek informatie bekend wordt die de toestemming van de proefpersoon zou kunnen beïnvloeden, dan breng ik hem/haar daarvan tijdig op de hoogte.

Naam onderzoeker (of diens vertegenwoordiger):

Handtekening: Datum: __ / __ / __

-----------------------------------------------------------------------------------------------------------------

* Doorhalen wat niet van toepassing is.

*De patiënt krijgt indien van toepassing, een volledige informatiebrief mee, samen met een kopie van het getekende toestemmingsformulier.*

Versie: 1.0 datum 13-08-2020

## S9 Information letter ITUMM study for patients

Deelname aan medisch-wetenschappelijk onderzoek over de impact van een e-coach

*Geachte heer/mevrouw,*

*U ontvangt deze brief omdat wij u willen vragen om uw schriftelijke toestemming om mee te doen aan een medisch-wetenschappelijk onderzoek. Aan de telefoon heeft u al aangegeven deel te willen nemen aan het testen van de SanaCoach bij de ziekte van Kahler (Multiple Myeloma).
Of u mee wilt doen, beslist u zelf. Deelname is vrijwillig. Wilt u meedoen, dan hebben wij hier uw schriftelijk toestemming voor nodig. Deze toestemming geeft u door het plaatsen van uw handtekening onder deze brief.

Voordat u beslist of u mee wilt werken, vertellen wij u graag wat het onderzoek inhoudt. Lees deze informatie rustig door. Heeft u nog vragen? Dan kunt u deze per mail stellen aan Job Eijsink, apotheker en onderzoeker, via dit e-mailadres: j.f.h.eijsink@isala.nl. Natuurlijk kunt u er ook over praten met uw partner, vrienden of familie.*

De officiële naam van het onderzoek is:

**The Impact of Telemonitoring on correct drug Use, complications, and quality of life among patients with Multiple Myeloma (MM)’ : (ITUMM)

Informatie over het onderzoek**

1. **Wie voert dit onderzoek uit?**

Dit onderzoek wordt uitgevoerd door Stichting Isala klinieken (“Isala”). De medisch ethische commissie heeft verklaard dat dit onderzoek uitgevoerd mag worden. Wilt u hier meer over weten? Dan vindt u meer informatie in de brochure ‘Medisch-wetenschappelijk onderzoek’.

1. **Wat is het doel van het onderzoek?**

We willen met dit onderzoek meer te weten komen over het gebruik en de impact van de e-coach. We meten uitkomstmaten om te kijken of de coach doet wat wij van de e-coach verwachten.

1. **Waarom is dit onderzoek nodig?**

Isala ontwikkelt verschillende e-coaches. Nu hebben we met een heel team aan mensen ook voor uw ziekte een e-coach ontwikkelt. Het unieke aan deze e-coach is dat deze nog niet in de wereld bestaat voor uw ziekte. Daarnaast hebben we in een speciaal traject ook samen met multipel myeloom patiënten bepaalt welke vragen de e-coach op welk moment zou moeten stellen. We verwachten dat als de e-coach doet wat deze hoort te doen, we sneller signalen binnenkrijgen waardoor we u beter kunnen helpen bij bijvoorbeeld pijn of uw gemoedstoestand.

1. **Wat houdt meedoen aan dit onderzoek in?**

Als u besluit om mee te doen, dan duurt uw deelname aan deze studie minimaal 12 maanden en maximaal 18 maanden.
In de eerste week krijgt u uitleg over de e-coach. Daarna worden er gedurende de studieperiode aan u vragen gesteld vanuit de e-coach. Zo kunnen wij de impact meten van de e-coach in uw zorgtraject in Isala.

**Heeft het meedoen aan het onderzoek invloed op mijn behandeling?**U ondergaat de huidige therapie zoals deze normaal ook wordt gegeven en krijgt een aantal keren in het jaar een aantal digitale vragenlijsten (controle groep) of u krijgt er de extra modules vanuit de coach aan toegevoegd die u helpen bij uw medicatie inname momenten, voorbereiding op het spreekuur, een berichtenservice en informatie over de ziekte (interventie groep). U wordt hiervoor gerandomiseerd (op basis van toeval in de interventie groep of de controle groep geplaatst).

**Heeft het meedoen aan het onderzoek invloed op mijn afspraken en metingen?**We proberen u op de ‘normale’ afspraken te laten komen, zoals u van ons gewend bent. Mocht u contact willen, dan mag u via de coach contact opnemen met de specialistisch verpleegkundige, of via telefoonnummer: 038 - 424 60 40

1. **Wat verwachten jullie tijdens het onderzoek van mij?**

Voor uw veiligheid en het onderzoek is het belangrijk dat u zich aan de volgende afspraken houdt:

- U gaat gedurende de komende 12 maanden met de e-coach werken;
- U vult op de afgesproken tijden de vragen in;
- U kunt via de e-coach al met zorgverleners communiceren. Lukt dit niet? Neem dan contact op via de gebruikelijke manier.

We willen u hartelijk danken voor uw medewerking.

Met vriendelijke groet,

Namens het hele ontwikkelteam

Job Eijsink, apotheker en onderzoeker

Bij vragen over dit onderzoek te bereiken op (j.f.h.eijsink@isalal.nl)

# REFERENCES

1. https://iknl.nl/nieuws/2019/fysiek-actieve-overlevenden-multipel-myeloom. (Accessed 29 January 2020).
2. Verelst SGR, Blommestein HM, Karim-Kos HE, Huijgens PC, Sonneveld P. Trends in incidence and survival of multiple myeloma in the Netherlands in the last wo decades, Results from a national population based study. Proceedings of the 53rd Annual Meeting of the American Society of Hematology (ASH), San Diego, CA; USA. Blood 2011; 118: Abstract 5071.
3. Cransac A, Aho S,Chretien M, Giroud M, Caillot D, Boulin M. Adherence to immunomodulatory drugs in patients with multiple myeloma. PLoS One. 2019 Mar 27;14(3):e0214446. doi: 10.1371/journal.pone.0214446.
4. Jong de MJ, Meulen-de Jong AE, Romberg-Camps MJ, Becx MC, Maljaars JP, Cilissen M, Bodegraven AA, Mahmmod N, Markus T, Hameeteman WM, Dijkstra G, Masclee AA, Boonen A, Winkens B, van Tubergen A, Jonkers DM, Pierik MJ. Telemedicine for management of inflammatory bowel disease (myIBDcoach): a pragmatic, multicentre, randomized controlled trial. Lancet 2017; 390: 959–68
5. Ethan Basch, Allison M. Deal, Mark G. Kris, Howard I. Scher, Clifford A. Hudis, Paul Sabbatini, Lauren Rogak, Antonia V. Bennett, Amylou C. Dueck, Thomas M. Atkinson, Joanne F. Chou, Dorothy Dulko, Laura Sit,Allison Barz, Paul Novotny, Michael Fruscione, Jeff A. Sloan, and Deborah Schrag. Symptom Monitoring With Patient-Reported Outcomes During Routine Cancer Treatment: A Randomized Controlled Trial. J Clin Oncol 34:557-565. © 2015 by American Society of Clinical Oncology.
6. Jacobs JM, Pensak NA, Sporn NJ, MacDonald JJ, Lennes IT, Safren SA, Pirl WF, Temel JS, Greer JA. Treatment Satisfaction and Adherence to Oral Chemotherapy in Patients With Cancer. J Oncol Pract. 2017 May;13(5):e474-e485. doi: 10.1200/JOP.2016.019729. Epub 2017 Apr 11.
7. Puts MTE, Tu HA, Tourangeau A, Howell D, Fitch M, Springall E, Alibhai H. Factors influencing adherence to cancer treatment in older adults with cancer: a systematic review. Ann Oncol. 2014 Mar; 25(3): 564–577. Published online 2013 Nov 26. doi: 10.1093/annonc/mdt433
8. Bassan F, Peter F, Houbre B, Brennstuhl MJ, Costantini M, Speyer E, Tarquinio C. Adherence to oral antineoplastic agents by cancer patients: definition and literature review. Eur J Cancer Care (Engl). 2014 Jan;23(1):22-35. doi: 10.1111/ecc.12124. Epub 2013 Sep 15
9. https://www.who.int/chp/knowledge/publications/adherence_introduction.pdf?ua=1 . chapter VII – Cancer palliative care pg 57-61 (Accessed 12-09-2019)
10. Nkansah N, Mostovetsky O, Yu C, Chheng T, Beney J, Bond CM, Bero L. Effect of outpatient pharmacists' non-dispensing roles on patient outcomes and prescribing patterns. Cochrane Database Systematic Review 2010
11. Ruddy K, Mayer E, Partridge A. Patient adherence and persistence with oral anticancer treatment. CA Cancer J Clin. 2009 jan-feb;59(1):56-66.
12. Patel K, Foster NR, Farrell A, et al. Oral cancer chemotherapy adherence and adherence assessment tools: a report from North Central Cancer Group Trial N0747 and a systematic review of the literature. J Cancer Educ. 2013 dec;28(4):770-6.
13. Marin D, Bazeos A, Mahon FX. Adherence is the critical factor for achieving molecular responses in patients with chronic myeloid leukemia who achieve complete cytogenetic responses on imatinib. J ClinOncol. 2010 mei 10;28(14):2381-8.
14. Krolop L, Ko YD, Schwindt PF, SchumacherC, Fimmers R, Jaehde U. Adherence management for patients with cancer taking capecitabine: a prospective two-arm cohort study. BMJ Open. 2013 jul 19;3(7). pii:e003139.
15. Cowan AJ,Allen C,Barac A. Global Burden of Multiple Myeloma, A Systematic Analysis for the Global Burden of Disease Study 2016. JAMA Oncol. 2018;4(9):1221-1227. doi:10.1001/jamaoncol.2018.2128.
16. Porter ME, Teisberg EO. Redefining healthcare: creating value-based competition on results. Boston: Harvard Business School Press, 2006.
17. Porter ME. Value-based health care delivery. Ann Surg 2008;248:503-9.
18. Bowman EH, Flood KL. Care transitions intervention and other nonnursing home transitions Models. Geriatrics Models of Care: Bringing ‘Best Practice’ to an Aging America. Cham, Switzerland: Springer International Publishing; 2015:97–114.
19. Ahmed F, Burt J, Rowland M: Measuring patient experience: concepts and methods. In The Patient – Patient-Centered Outcomes Research. doi:10.1007/s40271-014-0060-5. Published online: 16 May 2014.
20. Johansen NJ , Saunders CM. Value-Based Care in the Worldwide Battle Against Cancer. DOI: 10.7759/cureus.103
21. Stacey D, Legare F, Lewis K, Barry MJ, Bennett CL, Eden KB, et al. Decision aids for people facing health treatment or screening decisions. Cochrane Database Syst Rev. 2017 Apr 12;4:CD001431.
22. McAlpine K, Lewis KB, Trevena L, Stacey D. What is the effectiveness of patient decision aids for cancer-related decisions? A systematic review subanalysis. JCO Clinical Cancer Informatics. 2018:1-13.
23. Stiggelbout AM, Pieterse AH, De Haes JC. Shared decision making: Concepts, evidence, and practice. Patient Educ Couns. 2015 Oct;98(10):1172-9.
24. CCMO. No Title [Internet]. 2016. Available from: http://www.ccmo.nl/
25. www.icmje.org. Recommendations for the Conduct, Reporting, Editing, and Publication of Scholarly Work in Medical Journals. Citeseer. 2013;(December):1–17.
26. Moon JH, Sohn SK, Kim SN, et al. Patient counseling program to improve the compliance to imatinib in chronic myeloid leukemia patients. Med Oncol. 2012;29:1179-1185.
27. Joseph Mauro, Kelly B Mathews, Eric S Sredzinski. Effect of a Smart Pill Bottle and Pharmacist Intervention on Medication Adherence in Patients with Multiple Myeloma New to Lenalidomide Therapy. J Manag Care Spec Pharm 2019 Nov;25(11):1244-1254. DOI: 10.18553/jmcp.2019.25.11.1244
28. Lee Jeannie K, Grace Karen A, Terri G, Crawley Monica J, Erowele Goldina I, Sun Hazel J. How should we measure medication adherence in clinical trials and practice? Therapeutics and Clinical Risk Management 2007:3(4) 685–690
29. Ami J.Claxton, Joyce Cramer, Courtney Pierce, A systematic review of the associations between dose regimens and medication compliance; Clinical Therapeutics. Volume 23, Issue 8, August 2001, Pages 1296-1310. <https://doi.org/10.1016/S0149-2918(01)80109-0>
30. Hira Mian, Mark Fiala, Tanya M Wildes. Adherence to Lenalidomide in Older Adults With Newly Diagnosed Multiple Myeloma. Clin Lymphoma Myeloma Leuk. 2020 Feb;20(2):98-104.e. DOI: 10.1016/j.clml.2019.09.618
31. Hein A.W. van Onzenoort, Frederique E. Menger, Cees Neef, Willem J. Verberk, Abraham A. Kroon, Peter W. de Leeuw, and Paul-Hugo M. van der Kuy. Participation in a Clinical Trial Enhances Adherence and Persistence to Treatment. 2011 Hypertension. 2011;58:573–578.
32. N. Timilshina, H. Breunis, G. A. Tomlinson, J. M. Brandwein, R. Buckstein, S. Durbano & S. M. H. Alibhai. Long-term recovery of quality of life and physical function over three years in adult survivors of acute myeloid leukemia after intensive chemotherapy. Leukemia volume 33, pages15–25(2019).
33. Srinivas Raman, Keyue Ding, Edward Chow, Ralph M Meyer, Yvette M van der Linden , Daniel Roos, William F Hartsell, Peter Hoskin, Jackson S Y Wu Abdenour Nabid, Rick Haas , Ruud Wiggenraad , Scott Babington, William F Demas, Carolyn F Wilson, Rebecca K S Wong , Liting Zhu, Michael Brundage. Minimal clinically important differences in the EORTC QLQ-C30 and brief pain inventory in patients undergoing re-irradiation for painful bone metastases. Qual Life Res 2018 Apr;27(4):1089-1098. doi: 10.1007/s11136-017-1745-8.
34. Palumbo A, Anderson K. Multiple myeloma. N Engl J Med. 2011;364:1046-1060. SEER Cancer Stat Facts: Myeloma. National Cancer Institute.
35. S. Vincent Rajkumar. Updated Diagnostic Criteria and Staging System for Multiple Myeloma. 2016 ASCO EDUCATIONAL BOOK.
36. Ruddy Kathryn, Mayer Erica, Patridge Ann. Patient Adherence and Persistence With Oral Anticancer Treatment. 2009 American Cancer Society. Doi: 10.3322/caac.20004.
37. KNMP. No Title [Internet]. 2020. Available from <https://www.knmp.nl/praktijkvoering/bekostiging/begeleidingsgesprek-nieuw-geneesmiddel>
38. <https://doi.org/10.18553/jmcp.2019.25.11.1244>
39. <https://doi.org/10.1007/s00520-019-05133-0>
40. https://doi.org/10.1016/j.clml.2018.04.007
41. Rifkin RM, Abonour R, Terebelo H, et al. Connect MM registry: the importance

of establishing baseline disease characteristics. Clin Lymphoma Myeloma Leuk 2015;

15:368-76.

1. Shah JJ, Abonour R, Gasparetto C, et al. Analysis of common eligibility criteria of

randomized controlled trials in newly diagnosed multiple myeloma patients andextrapolating outcomes. Clin Lymphoma Myeloma Leuk 2017; 17:575-83.e572.

1. Piechotta V, Jakob T, Langer P, Monsef I, Scheid C, Estcourt LJ, Ocheni S, Theurich S, Kuhr K, Scheckel B, Adams A, Skoetz N

Multiple drug combinations of bortezomib, lenalidomide, and thalidomide for first-line treatment in adults with transplant-ineligible multiple myeloma: a network meta-analysis.
